# Diversification of the C-TERMINALLY ENCODED PEPTIDE (CEP) gene family in angiosperms, and evolution of plant-family specific CEP genes

**DOI:** 10.1186/1471-2164-15-870

**Published:** 2014-10-06

**Authors:** Huw A Ogilvie, Nijat Imin, Michael A Djordjevic

**Affiliations:** Research School of Biology, The Australian National University, Canberra, ACT 0200 Australia

**Keywords:** C-terminally encoded peptide, Gene family, Signaling peptides, GC-biased gene conversion, Panicle development, Orthology detection, Angiosperm evolution

## Abstract

**Background:**

Small, secreted signaling peptides work in parallel with phytohormones to control important aspects of plant growth and development. Genes from the C-TERMINALLY ENCODED PEPTIDE (CEP) family produce such peptides which negatively regulate plant growth, especially under stress, and affect other important developmental processes. To illuminate how the CEP gene family has evolved within the plant kingdom, including its emergence, diversification and variation between lineages, a comprehensive survey was undertaken to identify and characterize CEP genes in 106 plant genomes.

**Results:**

Using a motif-based system developed for this study to identify canonical CEP peptide domains, a total of 916 CEP genes and 1,223 CEP domains were found in angiosperms and for the first time in gymnosperms. This defines a narrow band for the emergence of CEP genes in plants, from the divergence of lycophytes to the angiosperm/gymnosperm split. Both CEP genes and domains were found to have diversified in angiosperms, particularly in the Poaceae and Solanaceae plant families. Multispecies orthologous relationships were determined for 22% of identified CEP genes, and further analysis of those groups found selective constraints upon residues within the CEP peptide and within the previously little-characterized variable region. An examination of public *Oryza sativa* RNA-Seq datasets revealed an expression pattern that links *OsCEP5* and *OsCEP6* to panicle development and flowering, and CEP gene trees reveal these emerged from a duplication event associated with the Poaceae plant family.

**Conclusions:**

The characterization of the plant-family specific CEP genes *OsCEP5* and *OsCEP6*, the association of CEP genes with angiosperm-specific development processes like panicle development, and the diversification of CEP genes in angiosperms provides further support for the hypothesis that CEP genes have been integral to the evolution of novel traits within the angiosperm lineage. Beyond these findings, the comprehensive set of CEP genes and their properties reported here will be a resource for future research on CEP genes and peptides.

**Electronic supplementary material:**

The online version of this article (doi:10.1186/1471-2164-15-870) contains supplementary material, which is available to authorized users.

## Background

The past decade has seen a paradigm shift in our understanding of plant growth and development. An important revelation has been the discovery of over 10 different multigene families that generate small secreted peptide signals as their mature biologically active products (hereafter referred to as signaling peptides). It is now recognized that these peptides work in parallel with phytohormones to control important aspects of plant growth and development [1–4]. Expression studies show that genes coding for signaling peptides are expressed in discrete locations [5], where the resulting peptides non-cell autonomously regulate biological and physiological processes which enable plants to develop and adapt to environmental changes [1, 6, 7]. Signaling peptides regulate processes of fundamental importance including cell proliferation and expansion, meristem maintenance, gravitropism, pollen guidance, fertilization, abscission, and the development of stomata, vascular tissues, root hairs, lateral roots and root nodules [1, 2, 4, 6–10].

One of these families is the C-TERMINALLY ENCODED PEPTIDE (CEP) gene family. CEP genes encode an *N*-terminal secretion signal (NSS), a variable domain, one or more CEP domains and a short *C*-terminal extension [11]. The 162 CEP genes discovered so far are single exon genes [6], and structure-activity studies suggest that the CEP domain is excised from the CEP prepropeptide to become a 15 amino acid (AA), post-translationally modified peptide [7, 11].

Several studies indicate that CEP peptides regulate plant root and shoot growth, and affect lateral root and root nodule development [6, 7, 9, 11]. Recently, a knockout of *AtCEP3* confirmed its role as a negative regulator of root development in response to abiotic stresses [6]. The paucity of CEP mutants has hampered detailed analyses of function, but overexpression and reporter gene studies suggest CEP peptides play important roles in a wide variety of processes in plants beyond controlling root growth and nodule development [6, 7, 9, 11].

Preliminary phylogenetic studies [6, 7, 9] identified many CEP genes in angiosperms and root-knot nematodes (RKN) with CEP domains similar to those from the original five CEP genes discovered in *Arabidopsis thaliana* [11]. Unlike the CLAVATA3/EMBRYO SURROUNDING REGION (CLE) multigene family, CEP genes were absent in the earliest diverging lineages of plants or in other nematodes (including the closely related false-RKN and the more distantly related cyst nematodes) [6, 9, 12, 13]. Those early studies also identified genes in angiosperms with domains more distant from the original CEP domains, termed “group II CEPs”, and in gymnosperms, termed “CEP-likes” [6, 9]. We term CEP genes which are structurally similar the originally discovered CEP genes “canonical CEPs”. For a gene to be classified as a canonical CEP, it must have an NSS, variable region, and a CEP domain close in sequence to the originally discovered CEP domains (i.e., not group II or CEP-like).

Previous studies of CEP genes in angiosperms used BLAST to identify CEPs in a haphazard way using both genomic and expressed sequence tag resources [6, 9]. In addition, it was not clear if canonical CEP genes are present in gymnosperms or to what extent CEP genes have diverged between plant families. The lack of a comprehensive survey of CEP genes across the plant kingdom has precluded an analysis of sequence diversity, orthology and selection pressure within this gene family. Recently, new and better methods of detecting small peptide encoding genes have been developed, based on motif identification and the prediction of one and two exon gene models [14].

Here, we have developed a motif identification system to detect CEP genes. An iteratively generated position-specific probability matrix (PSPM) was used to comprehensively identify canonical CEP genes using high quality genomic information from a broad range of plants. The diversification of CEP gene and CEP domain sequences was characterized by calculating intra-organism pairwise distances (IOPDs). CEP gene orthology was determined using an extended Reciprocal Best Hit (RBH) method capable of detecting orthologous genes in multiple species. The comprehensive identification of CEP genes and orthologous groups enabled selection pressure on residues along the entire CEP prepropeptide to be identified and quantified using ratios of non-synonymous to synonymous substitutions. The influence of GC bias on CEP peptide AA distribution was determined. For the first time, canonical CEP genes were identified in gymnosperms; this defines a new limit on the latest possible emergence of CEP genes. Finally, CEP gene expression in *Oryza sativa* was examined using publicly available RNA-Seq datasets to reveal a developmental transition in expression of CEP genes from *OsCEP6* in the booting panicle to *OsCEP5* in the flowering panicle, strongly suggesting these genes play a role in panicle development.

## Results

### Canonical CEP genes identified in gymnosperms and angiosperms

To identify canonical CEP genes, 106 plant genome assemblies (Additional file 1: Table S1) spanning 80 genera and 39 families across the plant kingdom were scanned for open reading frames (ORF) with an NSS and one or more canonical CEP domains. Using a PSPM iteratively generated from previously identified CEP domains (Additional file 2), 916 CEP genes and 1,223 CEP domains were identified across seed plants (Table 1). Previously unknown CEP genes were identified using this method even in well-studied model organisms, including two new genes in *Medicago truncatula*, *MtCEP12* and *MtCEP13*, and two new genes in *O. sativa*, *OsCEP6* and *OsCEP7* (Additional file 1: Table S2).

**Table 1:** **Summary of canonical CEP sequences identified in gymnosperms, angiosperms, and selected families within those clades**

	CEP genes	CEP domains	Domains per gene
Seed plants*	**916**	**1,223**	**1.34**
Angiosperms**	**860**	**1,167**	**1.36**
Brassicaceae	102	166	1.63
Fabaceae	124	149	1.20
Poaceae	86	95	1.10
Rosaceae	61	72	1.18
Solanaceae	70	70	1.00
Gymnosperms***	**56**	**56**	**1.00**
Pinaceae	55	55	1.00

*Seed plants include all angiosperms and gymnosperms.

**Angiosperms include Brassicaceae, Fabaceae, Poaceae, Rosaceae, Solanaceae and unlisted angiosperm families.

***Gymnosperms include Pinaceae and unlisted gymnosperm families.

Out of the 12 canonical CEP genes and three “group II” CEP genes previously identified in *A. thaliana* [6, 9], all 12 canonical genes were re-identified and all three group II genes were rejected using this method (Additional file 1: Table S2). This result supports the sensitivity and specificity of our method for identifying CEP genes, and reinforces a previous finding [9] that group II CEP genes are phylogenetically distinct from canonical CEP genes.

Our data confirmed the absence of CEP genes [6, 9] from the genome assemblies of the bryophyte moss *Physcomitrella patens* [15] or the lycophyte *Selaginella moellendorffii* [16]. 860 CEP genes were identified in angiosperms (Table 1), including 13 in the genome assembly of the basal angiosperm *Amborella trichopoda* [17] (Additional file 1: Table S2). For the first time, 56 canonical CEP genes were identified in gymnosperm genomes. These included 55 CEP genes from the conifer family Pinaceae (Table 1) and one CEP gene from a gymnosperm outside the Pinaceae family, in the low coverage genome assembly [18] of *Gnetum gnemon* (Additional file 1: Table S2).

**Figure 1:**
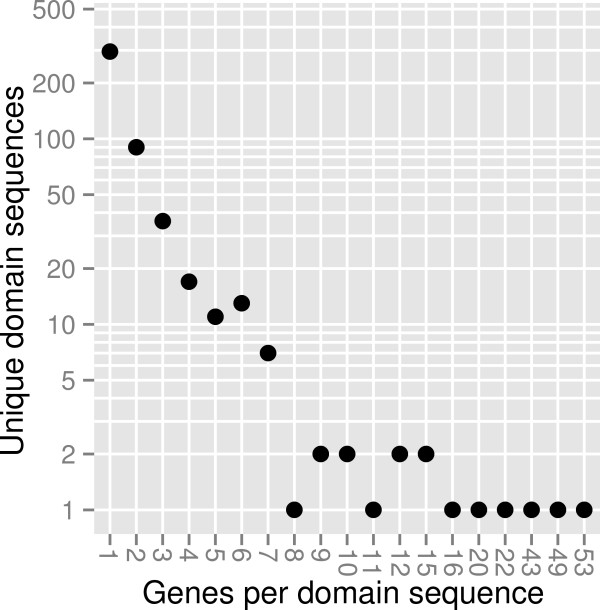
**The distribution of the number of genes associated with each unique domain sequence.** While 295 CEP domain sequences are present in only one CEP gene, 190 were present in multiple (sometimes many) CEP genes.

Many CEP domain sequences were shared by multiple genes across species; there were only 485 unique CEP domain sequences out of 1,223 total CEP domains identified. While the most common number of genes per domain sequence was one, 190 domain sequences were found in at least two genes and the most prevalent domain sequence (DFRPTAPGHSPGVGH) was identified in 53 different genes across the rosid clade of eudicots (Figure 1, Additional file 1: Table S3).

Notably, out of the CEP domains identified in the genome of the Pinaceae species *Pinus taeda* [19], two had an identical AA sequence (AFRPTSSGHSPGVGH) to a CEP domain identified in the genome of the angiosperm *Kalanchoe fedtschenkoi* [20]. The similarity of any given sequence to a PSPM can be quantified using a bit score, and therefore the bit score of a CEP domain compared to the canonical CEP PSPM is a measure of how CEP-like that domain is. By this measure, the domain sequence shared by *P. taeda* and *K. fedtschenkoi* was equal 19th most CEP-like out of the 485 unique CEP domain sequences (Additional file 1: Table S3). This analysis clearly demonstrates that canonical CEP genes are present in gymnosperms.

While all CEP genes identified in this study included a variable region, consistent with the five original CEP genes reported in *A. thaliana* [11], 53 CEP genes (representing 5.8% of all CEP genes identified in seed plants) were identified which lacked *C*-terminal extensions (Additional file 1: Table S2). These genes have a stop codon immediately after the *C*-terminal end of the 15 AA CEP domain, which defines the CEP domain’s right border.

### Diversification of CEP gene and domain sequences

To measure the diversity of CEP gene and CEP domain sequences, and the difference in sequence diversity between plant families, intra-organism pairwise distances (IOPD) were calculated. IOPDs are the genetic distances between all pairs of CEP genes and CEP domains identified in the genome assembly of an individual organism. For the families best represented among genome assemblies – the gymnosperm family Pinaceae, and the angiosperm families Brassicaceae, Fabaceae, Poaceae, Rosaceae and Solanaceae – IOPD distributions were generated by aggregating IOPDs by plant family.

**Figure 2:**
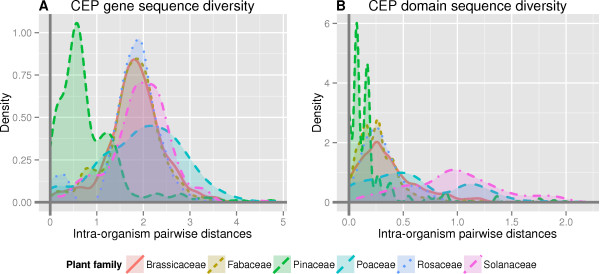
**A comparison of CEP gene (A) and CEP domain (B) sequence diversity between seed plant families.** These density plots of sequence diversity graph the distributions of pairwise genetic distances of CEP genes and domains within a single organism, aggregated by plant family. Genetic distances were calculated based on the AA sequences, using a maximum likelihood estimation. Tukey’s test reveals that both CEP gene and domain sequence diversity is significantly greater in all angiosperm families than in the gymnosperm family Pinaceae (*P* < 0.001). Additionally, the CEP gene and domain sequence diversity of Poaceae and Solanaceae are significantly (*P* < 0.05) greater than in other angiosperm families.

For both CEP genes and CEP domains, all five well-represented angiosperm families featured significantly (*P* < 0.001) more sequence diversity than Pinaceae. Intriguingly, the CEP gene and CEP domain sequence diversity of Solanaceae (within the eudicot and asterid clades) and Poaceae (within the monocot clade) were both significantly (*P* < 0.05) greater than Brassicaceae, Fabaceae or Rosaceae (Figure 2). Solanaceae and Poaceae do not share a common ancestor to the exclusion of the other well-represented families [21], so this pattern must require either independent increases in CEP sequence diversity in the Solanaceae and Poaceae lineages, or a loss of sequence diversity in the rosid clade which includes the other well-represented angiosperm plant families.

**Figure 3:**
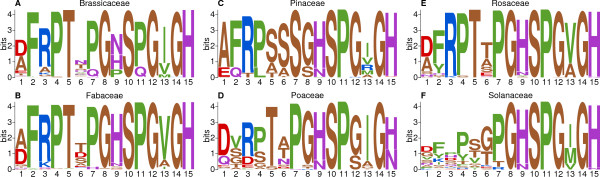
**Sequence logos of CEP domains by plant family.** These sequence logos, which visualize the distribution of AAs at each position of a short motif, are based on weighted CEP domain sequences identified in the genomes of the plant families Brassicaceae **(A)**, Fabaceae **(B)**, Pinaceae **(C)**, Poaceae **(D)**, Rosaceae **(E)** and Solanaceae **(F)**. AAs are colored by chemistry; small/non-polar (brown), hydrophobic (green), polar (purple), negatively charged (red), positively charged (blue). All AAs are represented as standard, single-letter abbreviations [22].

Patterns of conservation within the CEP domain are apparent when comparing PSPMs generated separately for each plant family – particularly the *C*-terminal residues at positions 7 through 15 (most commonly [PS]GHSPG[VI]GH) which are usually conserved within and between plant families (Figure 3). In contrast, the *N*-terminal residues from position 1 to 5 are notably more variable within Poaceae and Solanaceae CEP domains than in other families (Figure 3D and F), explaining the increased diversity seen in CEP domain IOPDs for Poaceae and Solanaceae. For example, in other families the residue at position 2 is usually phenylalanine, but can be other AAs in Solanaceae (Figure 3F), and is never phenylalanine in Poaceae (Figure 3D).

In addition, while position 6 shows a high degree of variability in all angiosperm plant families we analyzed, it is usually either serine or alanine in Pinaceae (Figure 3C). Another notable difference between angiosperm and gymnosperm CEP domains is at position 7, which is highly conserved as proline in angiosperm plant families (Figure 3A, B, D, E and F) but in Pinaceae (Figure 3C) is always serine. Finally, diversity in chemical properties is not necessarily correlated with diversity of AAs. For example, while the AA distribution at position 13 varies by plant family, three of the most common AAs at that position – isoleucine, valine and methionine – are all hydrophobic.

### Selective constraints on residues within and outside CEP domains

Orthologous groups of CEP genes were identified using the RBH method, extended to more than two organisms (see Methods). In angiosperms, 34 orthologous groups were identified. With one exception, all groups were restricted to species within a single plant family: either Brassicaceae, Cucurbitaceae, Fabaceae, Poaceae, Rosaceae or Solanaceae. Orthologous group 10 included genes from Brassicaceae as well as from the genome assembly of *Tarenaya hassleriana* [23], a species from the sister family to Brassicaceae, Cleomaceae. No orthologous groups were identified in gymnosperms. As only three high coverage gymnosperm genome assemblies – *P. taeda* [19], *Picea abies* [18] and *Picea glauca* [24] – are available, this may be due to the minimum requirement of four orthologs per group imposed in this study. In total, 202 CEP genes were classified into orthologous groups, 22% of all CEP genes identified in this study.

**Figure 4:**
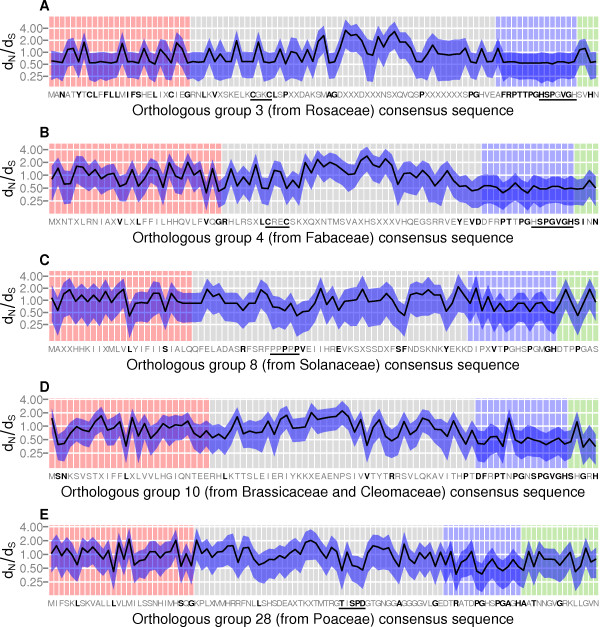
**d**_**N**_ **/d**_**S**_ **ratios of selection pressure across the amino acid sequences of exemplary orthologous groups of CEP genes.** d_N_/d_S_ means (black line) and 95% HPD intervals (blue ribbon) were calculated for orthologous groups of CEP genes. Orthologous groups were chosen from a diverse range of angiosperm families: group 3 from Rosaceae **(A)**, group 4 from Fabaceae **(B)**, group 8 from Solanaceae **(C)**, group 10 from the sister families Brassicaceae and Cleomaceae **(D)** and group 28 from Poaceae **(E)**. The entire coding region was analyzed, including the NSS (light red), variable region (grey), CEP domain (blue) and *C*-terminal extension (green). Significantly constrained residues (upper HPD limit < 1) are highlighted in bold. Conserved motifs within the variable region and related motifs in the CEP domain are underlined.

To identify any residues in the CEP prepropeptide that are under negative selection, ratios of non-synonymous to synonymous substitutions (d_N_/d_S_) were estimated for all residues of all orthologous groups (Additional file 3). Ratios below 1 indicate a selective constraint at that position, favoring specific AAs [25]. One exemplary orthologous group was selected from each well-represented angiosperm plant family. Significantly constrained residues were found across the CEP coding region in each exemplary group, including within the NSS (light red), variable region (grey), CEP domain (blue) and *C*-terminal extension (green) (Figure 4).

Although conserved residues were more common within the CEP domain than outside of it, short conserved motifs were identified in variable regions. These include the Cx_2_C motif present in orthologous groups 3 and 4 (Figure 4A and B). Combined with the Hx_5_H motif present within the CEP domains, this is reminiscent of a Cys_2_His_2_ zinc finger (ZnF) domain, but with a considerably longer gap between the second cysteine and first histidine than the 12 amino acid gaps seen in DNA and protein-binding Cys_2_His_2_ domains [26]. Orthologous group 8 includes a polyproline region (Figure 4C). Orthologous group 28 (Figure 4E) includes a cluster of conserved residues with the motif TxSPD in the variable region, which is potentially a binding site for post-translational modification by proline-directed kinases [27].

Consistent with the increased variation observed at position 2 of the CEP domain in Poaceae and Solanaceae, the highest probability density (HPD) interval for the d_N_/d_S_ ratio at that position of orthologous group 8 (from Solanaceae; Figure 4C) was 0.52 to 4.1, and for orthologous group 28 (from Poaceae; Figure 4E) was 0.42 to 1.97. As these intervals include 1.0, no evidence of a selective constraint at that position was observed for either group. However, the upper HPD limits for the d_N_/d_S_ ratio at position 2 of orthologous groups 3 (from Rosaceae; Figure 4A), 4 (from Fabaceae; Figure 4B) and 10 (from Brassicaceae and Cleomaceae; Figure 4D) were 0.99, 1.03 and 0.76 respectively. Since these limits are below 1 for groups 3 and 10, this is evidence of a selective constraint for phenylalanine at that position, which for group 4 is marginally non-significant. Despite the different AAs present at position 13 in different orthologous groups, a significant selective constraint was observed at all groups except group 8 (Figure 4).

### The relationship between GC content and CEP domain residues by plant family

**Figure 5:**
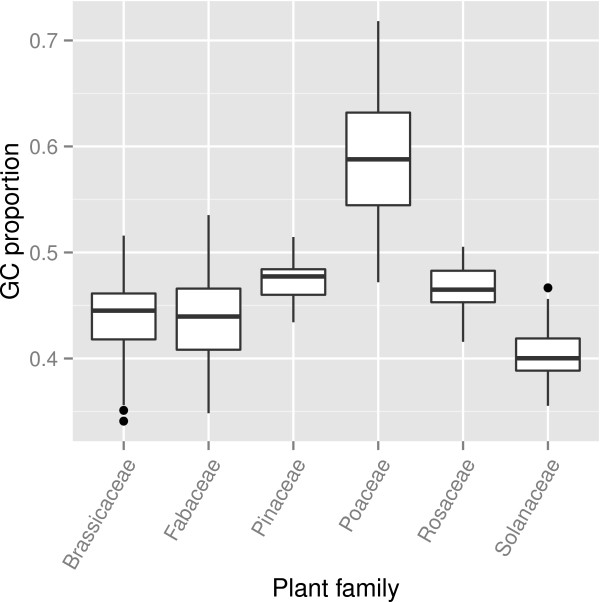
**GC content of CEP coding sequences in plant families.** Box plots show the median (center line), interquartile range (IQR, white box), range (whiskers) and outliers (circles). Outliers are defined as points more distant than 1.5 times the IQR from the median. Tukey’s test reveals that differences in GC content between all pairs of plant families except Brassicaceae-Fabaceae and Pinaceae-Rosaceae are statistically significant (*P* < 0.001).

To explore a potential basis for the different AA frequencies observed within the CEP domain for different plant families, the proportion of guanine and cytosine nucleotides (GC content) was calculated for all CEP genes identified in this study. Furthermore, the distribution of CEP gene GC content in the six well-represented plant families was calculated (Figure 5), and pairwise distances in GC content between those families statistically tested. The GC content of Poaceae CEP genes was significantly higher (*P* < 0.001) than any other plant family, and the GC content of Solanaceae CEP genes was significantly lower than any other plant family (*P* < 0.001).

**Figure 6:**
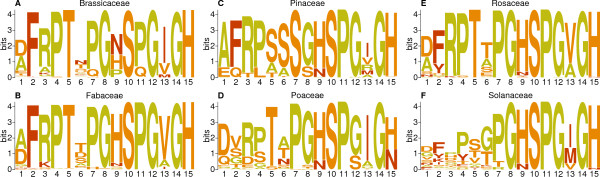
**Maximum GC content at each residue of the CEP domain in plant families.** These sequence logos are based on weighted CEP domain sequences identified in the genomes of the plant families Brassicaceae **(A)**, Fabaceae **(B)**, Pinaceae **(C)**, Poaceae **(D)**, Rosaceae **(E)** and Solanaceae **(F)**. AAs are colored by the maximum number of guanine and/or cytosine residues in the codons that encode each AA; 3 (green), 2 (orange) or 1 (red). All AAs are represented as standard, single-letter abbreviations [22].

The distributions of AAs at position 2 of the Poaceae and Solanaceae CEP domains (Figure 6) is consistent with the stark difference in GC content between CEP genes from those plant families. The most common AAs at this position in Poaceae – valine, serine, glycine and threonine – can be encoded using GC rich codons containing two or three guanine or cytosine nucleotides (Figure 6D). In contrast to this, the most common AAs at this position in Solanaceae – phenylalanine, tyrosine, lysine and isoleucine – can only be encoded using GC poor codons containing zero or one guanine or cytosine nucleotides (Figure 6F). However in position 13, where a selective constraint was often observed (Figure 4), the most common AA for both Poaceae and Solanaceae CEP domains was isoleucine, which utilizes GC poor codons (Figure 6D and Figure 6F).

### Expression analysis of the complete set of *Oryza sativa*CEP genes

**Figure 7:**
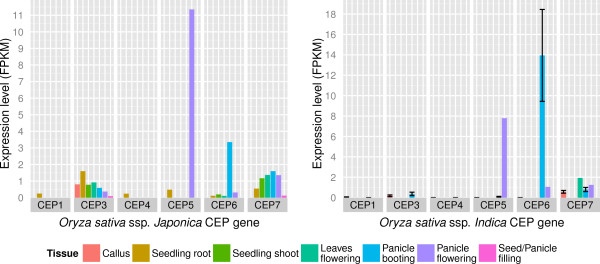
**CEP gene expression during** ***Oryza sativa*** **development, replicated in** ***Japonica*** **and** ***Indica*** **subspecies.** Alternating high expression of OsCEP6 during panicle booting, then OsCEP5 during panicle flowering, is consistent across subspecies. Error bars indicate standard error for tissues with replicates. N = 15 for *Indica* booting panicle, N = 14 for *Indica* callus, otherwise N = 1.

Given that CEP gene orthology was limited to single plant families, or at most sister plant families, this suggests some CEP genes could be phylogenetically and functionally unique to specific plant families. To investigate this possibility using a well-studied model organism, public RNA-Seq datasets were reanalyzed to measure the expression of all CEP genes identified in the Poaceae species *O. sativa*. A key feature of the Poaceae family is the development of grain-type seeds, and in *Oryza* these develop on characteristic loose panicles. Independent studies have been conducted using RNA-Seq to investigate the transcriptomes of different tissues of the two *O. sativa* subspecies, *Indica* [28] and *Japonica* [29]. The reanalysis uncovered a distinctive pattern of CEP gene expression in both subspecies, where *OsCEP6* is predominantly expressed in the (earlier) booting panicle, and *OsCEP5* is predominantly expressed in the (later) flowering panicle (Figure 7). This expression pattern implies a possible role for those CEP genes in panicle development.

### Kingdom-wide phylogenetic trees of the CEP gene family

**Figure 8:**
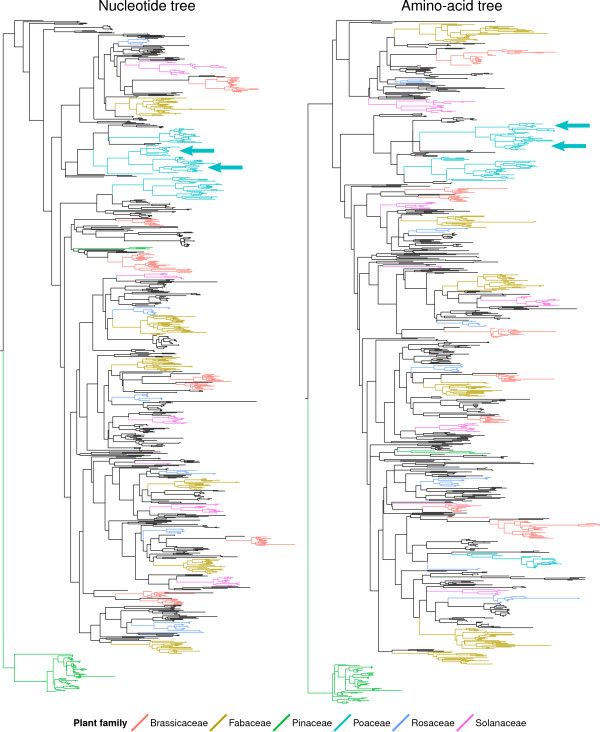
**Nucleotide and amino acid maximum likelihood trees of all identified CEP genes.** Maximum likelihood trees were computed based on aligned, non-redundant CEP gene sequences. Both trees were rooted using the largest cluster of gymnosperm CEP genes. The putative panicle development associated genes *OsCEP5* and *OsCEP6* (cyan arrows) are part of a single cluster of Poaceae CEP genes.

To infer when *OsCEP5* and *OsCEP6* emerged in the evolutionary history of plants, maximum likelihood phylogenetic genes of all CEP genes identified in this study were reconstructed based on both AA and nucleotide sequences. In both AA and nucleotide trees, *OsCEP5* and *OsCEP6* were located within a single cluster of Poaceae CEP genes (Figure 8). Bootstrap support values were calculated for both trees, and in the AA tree the cluster has a strong support value of 91 (Additional file 4), while in the nucleotide tree it has a weak support value of 49 (Additional file 5).

**Figure 9:**
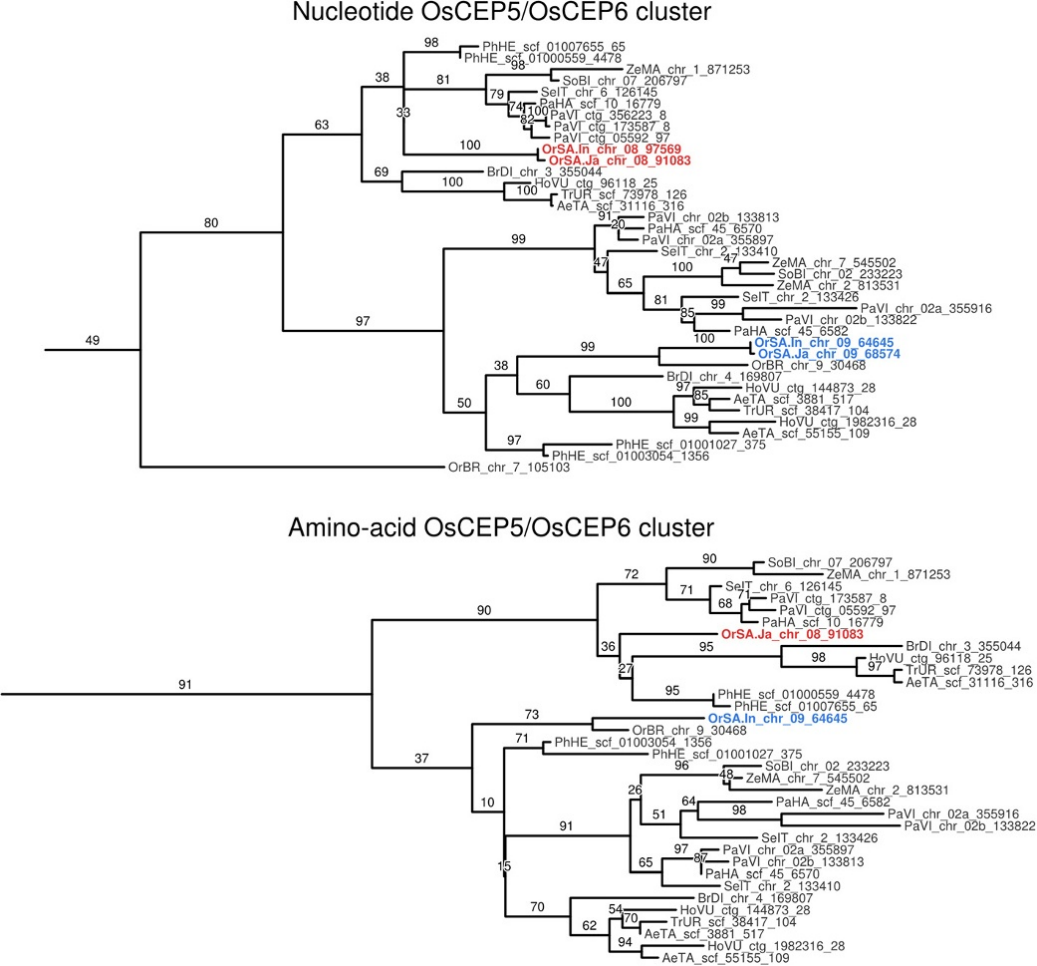
**The** ***OsCEP5*** **/** ***OsCEP6*** **lineage of Poaceae CEP genes from nucleotide and amino-acid CEP gene trees.** *OsCEP5* genes from *Oryza sativa* ssp. *Indica* and ssp. *Japonica* are highlighted in blue. *OsCEP6* genes from the same subspecies are highlighted in red. As the AA sequences of *Indica OsCEP5* and *OsCEP6* are identical to their *Japonica* orthologs, a single sequence representing both subspecies was used for each gene when generating the AA maximum likelihood tree.

A closer inspection revealed that *OsCEP5* and *OsCEP6* were placed into separate lineages within each cluster, and each lineage includes CEP genes from a broad range of Poaceae species (Figure 9). This is indicative of a gene duplication event having occurred prior to the evolution of Poaceae, or early in the Poaceae lineage. This identification of *OsCEP5* and *OsCEP6* as sister paralogs, and their conservation throughout Poaceae, suggests they may play a role in inflorescence development in other Poaceae species besides *O. sativa*.

Relationships between clusters of CEP genes from different plant families are difficult to resolve due to low bootstrap support values (Additional files 4 and 5). This may be due to the short length of the CEP coding sequence, leading to an insufficient number of phylogenetically informative residues to resolve the deep relationships between plant-family specific clusters. This is highlighted by the shortest CEP coding sequence identified, which is from the genome assembly of *Azadirachta indica* [30]. Its CEP coding sequence is 48 AAs in length, including an NSS of 19 AAs, a variable region of 10 AAs, a CEP domain of 15 AAs and a *C*-terminal extension of 4 AAs (Additional file 1: Table S2).

## Discussion

### Our method identified canonical CEP genes across angiosperms and gymnosperms, placing an earlier limit on CEP gene emergence

Previous studies have used BLAST to identify CEP genes in a haphazard way across existing sequence databases [6, 9]. By developing a systematic approach where essential features of a CEP gene (the NSS and CEP domain) are scanned for in all ORFs, 916 CEP genes were identified, greater than five-fold the number of genes identified in a previous study [6]. This includes genes from species where canonical CEP genes have not been previously identified, most importantly gymnosperms including *G. gnemon* and Pinaceae species, demonstrating that CEP genes are not limited to angiosperms but are present across seed plants.

The absence of CEP genes from the bryophyte moss *P. patens* or the lycophyte *S. moellendorffii* places a limit on earliest emergence of CEP genes after the divergence of those lineages, which is later than CLE genes which are present in both plants [31]. The positive identification of CEP genes in gymnosperms in this study places a limit on the latest emergence of CEP genes at the point of angiosperm/gymnosperm divergence. Further research is needed to determine whether CEP genes are present in monilophytes, which emerged after lycophytes but before the angiosperm/gymnosperm divergence [21]. The methods used in this study can be applied to monilophyte genome assemblies when they become available.

Additional CEP genes were also identified in previously analyzed species, for example the *OsCEP6* gene in *O. sativa*, as well as additional CEP genes in the model legume *M. truncatula*. The comprehensive database of CEP genes and domains identified in this study (Additional file 1) will therefore be a resource to researchers working on CEP genes in both model and non-model organisms.

### CEP genes have diversified in angiosperms, especially in Solanaceae and Poaceae

The comprehensive identification of CEP genes across a broad range of seed plants has enabled a partial elucidation of the evolutionary relationships between those genes. The newly identified gene *OsCEP6* emerged from a gene duplication event in the Poaceae plant family, which also produced the previously identified gene *OsCEP5*. The expression pattern of these genes points to a role in panicle development, and their conservation within Poaceae points to a role throughout that family. These paralogs demonstrate that at least some CEP genes are plant family specific, rather than being conserved in sequence and function across seed plants or across angiosperms.

To an extent the evolution of plant-family specific genes parallels the evolution of the CLE gene family. Some CLE peptides present in the Poaceae species *O. sativa* lack close relatives in the Brassicaceae species *A. thaliana* [32], and three separate CLE genes in *O. sativa* regulate meristem maintenance in the shoot apical, inflorescence and floral meristems, instead of the single *CLAVATA3* (*CLV3*) gene which serves that function in *A. thaliana* [33]. Multiple nodule-specific CLE genes have been identified in legumes which regulate nodulation in response to nitrate levels, a process specific to nodulating plants (mostly found in the Fabaceae family) [34].

However, the poor sequence diversity of CEP genes and CEP domains in gymnosperms differs from the CLE gene family, which has been found to be as diverse in the Pinophyta clade of gymnosperms as it is in angiosperms [35]. The lack of broadly conserved CEP domain sequences shared by gymnosperms and angiosperms differs from CLE peptides, many of which are closely conserved between Pinophyta (including Pinaceae) gymnosperms and the angiosperm *A. thaliana*. One important CLE peptide, TRACHEARY ELEMENT DIFFERENTIATION INHIBITORY FACTOR (TDIF), is perfectly conserved in sequence between Pinophyta and many angiosperm species including *A. thaliana* [35, 36].

This diversification of CEP genes and peptides in angiosperms (and the further diversification observed within Solanaceae and Poaceae), and the association of CEP genes with angiosperm-specific development, suggests that CEPs have been integral to the evolution of novel traits within the angiosperm lineage. Apart from the emergence of the Poaceae-specific CEP genes *OsCEP5* and *OsCEP6* and their association with panicle development in *O. sativa*, CEP genes have also been implicated in legume nodule development [7] and in floral development [9], all angiosperm-specific processes.

### Selective constraints on CEP residues support functional differences deriving from differences in amino acid sequence

Identification of orthologous groups of CEP genes and the characterization of selection pressure within the CEP domains of those groups has revealed key drivers of AA distributions and increased CEP domain sequence diversity. In orthologous groups from plant families that exhibit the highest CEP gene and CEP domain sequence diversity, Poaceae and Solanaceae, no evidence for selective constraint was observed at position 2 of the CEP domain, consistent with the greater variability observed at that residue and at positions 1–5 generally in those families.

Interestingly, AA distributions at position 2 differ between Poaceae and Solanaceae. In the absence of selective constraint, GC-biased gene conversion may alter the AA distribution at CEP peptide sites in Poaceae, by selecting for codons with higher GC content. GC-biased gene conversion is stronger in Poaceae than in other plant families [37], and the high GC content of Poaceae CEP genes reflects this phenomenon.

Across plant families, selective constraint was observed at position 13 of the CEP domain, which is typically a hydrophobic residue. However the exact residue varies between orthologous groups, suggesting that differences in CEP peptide sequences at this residue may be important to function.

The presence of conserved motifs within the variable regions of orthologous groups suggests that those regions may also have a functional role beyond simply linking the NSS and CEP peptide domain. Biochemical investigation is needed to determine the function of conserved motifs observed within the variable region, including their influence on protein folding, post-translational modification and binding properties.

Recently a secreted kinase was identified which is responsible for the phosphorylation of proteins targeted to the ER-dependent secretion pathway [38], and therefore potentially phosphorylated conserved motifs within variable regions could be sites targeted during secretion for regulation or processing of the CEP prepropeptide. Another avenue of investigation following the results of this study is whether the Cys_2_His_2_-like motifs identified in orthologous groups 3 and 4 bind ions in the same way as ZnF domains, despite the longer gap between the pair of cysteine and the pair of histidine residues in CEP genes relative to the gap in ZnF domains.

Homopolymeric proline repeats have been observed in the CLE gene OsCLE502, where they act as linkers between multiple CLE domains [39]. However, the polyproline sequence in CEP orthologous group 8 from Solanaceae is located in the variable region, and this orthologous group only encodes a single CEP domain. Regardless of whether homopolymeric repeats can be functional, the bulk of evidence suggests they can evolve neutrally [40].

Both within the CEP domain and within the variable region, selective constraints at sites which differ between orthologous groups could contribute to functional specificity deriving from the coding sequence of CEP genes, rather than their expression patterns alone. This is supported by a previous study that showed different CEP genes driven by a 35S overexpression promoter produced different phenotypes in *A. thaliana* [6].

## Conclusions

Until recently, identification of signaling peptide gene family members, including CEP genes, has been based on searching public sequence databases for homologous sequences. Now that many plant genome assemblies are available, we have systematically identified many canonical CEP genes by searching for their two essential domains (NSS and CEP) in the ORFs of those assemblies. This has confirmed the presence of CEP genes in gymnosperms, placing a new limit on the latest possible emergence of CEP genes.

By studying multiple aspects of the molecular evolution of CEP genes (orthology, selection pressure, IOPDs and gene trees), we can conclude that CEP genes have diversified in angiosperms, particularly in the Poaceae and Solanaceae plant families. We have also demonstrated that the *OsCEP5* and *OsCEP6* genes evolved with the Poaceae plant family, and are implicated in panicle development. Together with previous CEP reporter gene studies implicating CEP genes in other angiosperm-specific processes, this strongly suggests that the diversification of CEP genes has contributed to the evolution of angiosperms.

Finally, our approach can be applied to the study of other signaling peptide gene families, to shed further light on the evolutionary aspects and putative functions of signaling peptides in plants.

## Methods

### Generating position specific probability matrices

PSPMs, which describe the AA distribution at each site of a motif, were generated using the motif discovery tool MEME [41]. An initial PSPM of the CEP peptide domain was generated from an unweighted set of all previously identified [6] canonical CEP peptide domains. An improved second iteration of the CEP PSPM was generated from a weighted set of all CEP domains identified in plant genomes using the first PSPM. The CEP domains of specific plant families were characterized by generating separate PSPMs for each family, based on weighted sets of all CEP domains identified in those plant families using the second CEP PSPM.

For weighted sets, CEP domains weights were adjusted so that the weight for each CEP domain within a single ORF, the sum of weights for each ORF within a single genome assembly, as well as the sum of weights for each genome assembly were equal. A second weighting was applied to correct for over- and under-represented lineages using a species tree (Additional file 6). Over-represented lineages will contain more internal nodes, so CEP domain weights were reduced by 20% for each internal node from the root of the tree to each CEP domain’s associated organism.

The species tree used when weighting CEP domains was based on a previous study on the relationship between plant families [21], and previous studies on the relationship between species of gymnosperms [42], Brassicaceae and Cleomaceae [43], Cucurbitaceae [44], Euphorbiaceae [45], Fabaceae [46], Fragaria [47], Lamiales [48], Malpighiales [49], Nicotiana [50], Oryza [51], Poaceae [52], Rosaceae [53], Solanaceae [54] and the Triticum-Aegilops complex [55].

### Identifying CEP genes and CEP domains in plant genome assemblies

Pseudomolecules of the *M. truncatula* genome assembly version 4.0 [56] contained CEP genes absent from version 3.5.2 [57], but version 3.5.2 included unanchored BACs and unplaced contigs containing CEP genes absent from version 4.0. Through visual inspection of preliminarily identified *M. truncatula* CEP sequences, we verified that no CEP genes from version 4.0 pseudomolecules were present in version 3.5.2 unanchored BACs and unplaced contigs, and *vice versa*. In order to analyze the most complete set of *M. truncatula* CEP genes, a hybrid assembly was produced consisting of version 4.0 pseudomolecules and version 3.5.2 unanchored BACs and unplaced contigs. A second round of visual inspection of CEP sequences identified in the hybrid assembly confirmed that version 4.0 pseudomolecule CEPs and version 3.5.2 unanchored BAC/unplaced contig CEPs were mutually exclusive.

The *M. truncatula* hybrid genome and all other plant genome FASTA files were homogenized and concatenated using a custom Python script (Additional file 7). ORFs longer than 50 AAs between in-frame stop codons were extracted using the EMBOSS tool getorf [58]. This enabled the detection of very short CEP genes, including genes where the coding sequence (CDS) was less than 50 AAs in length, as long as the CDS was contained within a genomic sequence of at least 50 AA in length uninterrupted by stop codons. Extracted ORFs were interleaved across 100 output files so that later analysis would fit in available RAM. CEP domains were identified within extracted ORFs using the motif scanning tool FIMO [59], with a conservative *P*-value cut-off of 6 × 10^-11^.

CEP ORFs should contain an NSS which begins at the *N*-terminus. As a diverse range of plant genes utilizing start codons which typically code for leucine have been identified [60–63], the NSS might begin at any in-frame methionine or leucine codon before the CEP domain. To test for the presence of NSSs and to identify the start codon of each CEP ORF, the NSS identification tool SignalP 4.1 [64] was used to quantify the likelihood of an NSS at every possible start codon. ORFs where no start codon had a SignalP score above 0.400 were unlikely to contain an NSS and discarded. Otherwise, the possible start codon with the highest SignalP score was used to define the 5′ end and *N*-terminus of the nucleotide and AA sequence respectively.

### Calculating and comparing pairwise genetic distances

The genetic distance between all possible pairs of CEP genes and domains identified was estimated using the maximum likelihood algorithm implemented by PAML and the JTT AA substitution matrix [65, 66]. Global alignments of AA sequence pairs for use with PAML were generated using the L-INS-i algorithm implemented in MAFFT [67] and the JTT substitution matrix.

The diversity of CEP genes and domains within specific plant families (IOPDs) were calculated by aggregating the genetic distances between CEP genes and between CEP domains from the same genome assembly by plant family. Density plots of the distributions of aggregated pairwise distances were generated using ggplot2 [68]. To calculate statistical differences between those distributions, pairwise distances were first rank-transformed due to the multimodality, skewing and unequal variances observed in the density plots. Tukey’s test was used to calculate multiple-testing corrected *P*-values for all pairwise comparisons of plant families.

### Identifying orthologous relationships between CEP genes

Orthologous groups of CEP genes were identified by extending the RBH method of identifying orthologous genes shared by two species [69]. First, RBH pairs of CEP genes between all species were identified using BLASTP [70], and a graph assembled of those pairs. Second, RBH pairs from the lowest to highest BLAST bit scores were checked to ensure that both genes in the pair share the same set of orthologs. When another gene is orthologous to only one gene of the original pair, the two genes of the RBH pair may belong to different orthologous groups, and the edge connecting the pair was deleted. After an edge was deleted, this process was repeated from the second step until both genes from all remaining pairs shared identical sets of orthologs. By pruning edges connecting potentially non-orthologous RBH pairs, the graph was reduced to clusters of genes within which every gene is connected to every other gene. Clusters of more than four genes were considered orthologous groups and labelled sequentially.

This algorithm is a special case of a previously described clustering RBH (cRBH) algorithm, which left unspecified the order in which RBH pairs were compared with other genes, and therefore which edges are deleted to produce the reduced graph [71]. By repeatedly evaluating RBH pairs in ascending order of their BLAST bit scores, the method in this study is deterministic and reproducible.

### Calculating ratios of non-synonymous to synonymous substitutions (d_N_/d_S_)

Conservation of residues in orthologous groups was identified using d_N_/d_S_ ratios of non-synonymous to synonymous substitutions. The renaissance counting feature of the Bayesian phylogenetics package BEAST [72] was used to estimate d_N_/d_S_ ratios and 95% HPD intervals for each orthologous group of CEP genes. A multiple sequence alignment of each orthologous group was generated using the L-INS-i algorithm and the JTT substitution matrix. Poorly aligned columns were excised using the gappyout algorithm implemented by TrimAl [73]. For all groups, parameters were estimated using an HKY substitution model [74] with empirical base frequencies, a chain length of 10^8^ steps and a 10% burn-in. Graphs of d_N_/d_S_ ratios, HPD intervals and consensus sequences (generated using the EMBOSS tool cons [58]) were again generated using ggplot2.

### Comparing the GC content of CEP genes in different plant families

The number of guanine and cytosine nucleotides as a proportion of all nucleotides (GC content) was calculated for each CEP gene. For specific plant families, the distribution of GC content in all genes in each family was visualized as a boxplot using ggplot2. To calculate statistical differences between those distributions, a logit transformation was applied to GC content to change proportions (which are bounded by 0 to 1) into log-odds (which are unbounded). Tukey’s test was used to calculate multiple-testing corrected *P*-values for all pairwise comparisons of plant families.

### Calculating differential expression of CEP genes

To calculate the expression levels of CEP genes, RNA-Seq reads were remapped to their reference genomes using the short-read aligner SMALT [75] with default settings. Then, based on the CEP genes identified, general feature format (GFF) files were generated for each assembly which specified CEP mRNA regions as beginning 100 nucleotides upstream of the start codon and 300 nucleotides downstream of the stop codon. The mapped reads and GFF files were used as input for the cuffdiff program from the Cufflinks package [76], to calculate CEP gene expression as fragments per kilobase of exon model per million reads (FPKM).

### Reconstructing the phylogenetic tree of the CEP gene family

The phylogenetic history of the CEP gene family across all plants was reconstructed using a maximum likelihood approach. First, ambiguous characters were stripped from the sequences of CEP genes identified in this study. Multiple sequence alignments (MSA) of all nucleotide sequences were generated using L-INS-i, and all AA sequences were aligned using L-INS-i and the JTT substitution matrix. Poorly aligned columns from both nucleotide and AA MSAs were excised using the gappyout algorithm implemented by TrimAl. 100 bootstrap replicates and maximum parsimony starting trees of the original and bootstrap sequence alignments were generated using the RAxML phylogenetic software [77]. Unrooted gene trees were reconstructed for the original MSA and each bootstrap replicate using the phylogenetic software ExaML [78]. The nucleotide and AA maximum likelihood trees were generated based on the general time reversible and JTT substitution models respectively. Both were generated using the CAT model of rate heterogeneity [79]. Finally, bootstrap support values were added to the maximum likelihood trees of the original sequence alignments using the phylogenetic tree summarization program SumTrees [80].

## Electronic supplementary material

Additional file 1: **Additional tables.** Includes genome assemblies used in this study **(Table S1)**, CEP genes identified in those genomes (with symbolic names for CEP genes found in the model species *A. thaliana, M. truncatula and O. sativa*) and their properties **(Table S2)**, and identified CEP domains with their bit scores and associated CEP genes **(Table S3)**. (XLSX 330 KB)

Additional file 2: **MEME output for the canonical CEP domain.** Includes the CEP domain sequence logo and position-specific probability matrix (PSPM), iteratively generated from previously identified CEP domain sequences. (ZIP 108 KB)

Additional file 3: **d**_**N**_ **/d**_**S**_ **ratios for orthologous groups.** Each table contains mean values and high probability density (HPD) intervals of d_N_/d_S_ ratios, across all residues of the CEP prepropeptide for each orthologous group. (XLSX 211 KB)

Additional file 4: **Viewable with FigTree [** http://tree.bio.ed.ac.uk/software/figtree/ **].** CEP gene tree generated using amino acid sequences, with bootstrap support values. (ZIP 124 KB)

Additional file 5: **Viewable with FigTree [** http://tree.bio.ed.ac.uk/software/figtree/ **].** CEP gene tree generated using nucleotide sequences, with bootstrap support values. (ZIP 144 KB)

Additional file 6: **Viewable with FigTree [** http://tree.bio.ed.ac.uk/software/figtree/ **].** Rooted phylogenetic tree of plants used to weight CEP genes identified by organism. (ZIP 2 KB)

Additional file 7: **Can be extracted using 7-Zip [** http://www.7-zip.org/ **].** All Python, R and shell scripts used to analyze genomes and generate the results for this study. (ZIP 37 KB)
