# Supplementary material for: Diversification of the C-TERMINALLY ENCODED PEPTIDE (CEP) gene family in angiosperms, and evolution of plant-family specific CEP genes
Source: BMC Genomics. 2014 Oct 6;15(1):870. doi: 10.1186/1471-2164-15-870 (PMC4197245; doi:10.1186/1471-2164-15-870)
Supplement: Supplementary file 2 — Additional file 2: MEME output for the canonical CEP domain. Includes the CEP domain sequence logo and position-specific probability matrix (PSPM), iteratively generated from previously identified CEP domain sequences. (ZIP 108 KB) [file 12864_2014_6540_MOESM2_ESM.zip › index.html]

MEME


Discovered Motifs
  |  
Program information
  |  
Explanation

## Canonical CEP domain

| Summary | Sequence Logo |
| --- | --- |
| |  |  | | --- | --- | | E-value | 2.1e-10668 | | Width | 15 | | Sites | 1189 |  show more | Log Likelihood Ratio | | | --- | --- | |  | 29174 | | Information Content | | |  | 49.0 (bits) | | Relative Entropy | | |  | 35.4 (bits) | show less |  |

#### Regular expression

[DA]FRPT[ST][PS]GHSPG[VI]GH

#### Data Formats

View the motif in
PSPM Format 
    
PSSM Format 
    
BLOCKS Format 
    
FASTA Format 
    
Raw Format 
    
or
Hide  
This feature requires javascript.

#### Sites

Click on any row to highlight sequence in all motifs.

| Name | Start | *p*-value | Sites | | |
| --- | --- | --- | --- | --- | --- |
| MiGU\_scf\_11\_31507\_103 | 1 | 1.15e-15 |  | DFRPTSPGHSPGIGH |  |
| CiSI\_scf\_00006\_4959\_142 | 1 | 1.15e-15 |  | DFRPTSPGHSPGIGH |  |
| CiCL\_scf\_7\_106263\_142 | 1 | 1.15e-15 |  | DFRPTSPGHSPGIGH |  |
| BrRA\_chr\_07\_114992\_118 | 1 | 1.15e-15 |  | DFRPTSPGHSPGIGH |  |
| TaHA\_scf\_3\_40860\_86 | 1 | 1.24e-15 |  | DFRPTEPGHSPGVGH |  |
| PoTR\_chr\_07\_72949\_105 | 1 | 1.24e-15 |  | DFRPTEPGHSPGVGH |  |
| MaES\_chr\_xviii\_47391\_89 | 1 | 1.24e-15 |  | DFRPTEPGHSPGVGH |  |
| LuAN\_scf\_72448\_2\_5\_85 | 1 | 1.24e-15 |  | DFRPTEPGHSPGVGH |  |
| EuGR\_scf\_9\_63890\_152 | 1 | 1.24e-15 |  | DFRPTEPGHSPGVGH |  |
| EuGR\_scf\_8\_418858\_175 | 1 | 1.24e-15 |  | DFRPTEPGHSPGVGH |  |
| EuGR\_scf\_6\_193586\_146 | 1 | 1.24e-15 |  | DFRPTEPGHSPGVGH |  |
| EuCA\_ctg\_025488\_37\_129 | 1 | 1.24e-15 |  | DFRPTEPGHSPGVGH |  |
| ViAN\_ctg\_11585\_0\_37\_111 | 1 | 2.86e-15 |  | DFRPTSPGHSPGVGH |  |
| ThCA.Ma\_chr\_1\_202497\_88 | 1 | 2.86e-15 |  | DFRPTSPGHSPGVGH |  |
| ThCA.Cr\_chr\_01\_161958\_88 | 1 | 2.86e-15 |  | DFRPTSPGHSPGVGH |  |
| MeTR\_scf\_ac233112.6\_695\_68 | 1 | 2.86e-15 |  | DFRPTSPGHSPGVGH |  |
| LuAN\_scf\_76092\_52\_84 | 1 | 2.86e-15 |  | DFRPTSPGHSPGVGH |  |
| LeAL\_scf\_12\_1266\_85 | 1 | 2.86e-15 |  | AFRPTEPGHSPGIGH |  |
| LaSI\_ctg\_301883\_1\_91 | 1 | 2.86e-15 |  | DFRPTSPGHSPGVGH |  |
| GlMA\_chr\_11\_11263\_104 | 1 | 2.86e-15 |  | DFRPTSPGHSPGVGH |  |
| GlMA\_chr\_01\_174721\_105 | 1 | 2.86e-15 |  | DFRPTSPGHSPGVGH |  |
| FrEX\_scf\_20018\_3\_122 | 1 | 2.86e-15 |  | DFRPTSPGHSPGVGH |  |
| EuSA\_scf\_5\_91806\_71 | 1 | 2.86e-15 |  | AFRPTEPGHSPGIGH |  |
| CuSA\_chr\_6\_94315\_123 | 1 | 2.86e-15 |  | DFRPTSPGHSPGVGH |  |
| CuME\_chr\_08\_112760\_85 | 1 | 2.86e-15 |  | DFRPTSPGHSPGVGH |  |
| CiLA\_chr\_1\_108400\_72 | 1 | 2.86e-15 |  | DFRPTSPGHSPGVGH |  |
| CiAR.Ka\_chr\_7\_28093\_139 | 1 | 2.86e-15 |  | DFRPTSPGHSPGVGH |  |
| CaSA.PK\_scf\_79209\_8\_112 | 1 | 2.86e-15 |  | DFRPTSPGHSPGVGH |  |
| CaSA.Fi\_ctg\_14480433\_2\_119 | 1 | 2.86e-15 |  | DFRPTSPGHSPGVGH |  |
| SiIR\_scf\_8\_11134\_77 | 1 | 5.35e-15 |  | DFRPTTPGHSPGIGH |  |
| SeIN\_lg\_8\_120425\_83 | 1 | 5.35e-15 |  | DFRPTTPGHSPGIGH |  |
| SeIN\_lg\_15\_9726\_84 | 1 | 5.35e-15 |  | DFRPTTPGHSPGIGH |  |
| ScPA\_chr\_2-4\_32057\_77 | 1 | 5.35e-15 |  | DFRPTTPGHSPGIGH |  |
| MiGU\_scf\_8\_8694\_84 | 1 | 5.35e-15 |  | DFRPTTPGHSPGIGH |  |
| MiGU\_scf\_14\_91504\_107 | 1 | 5.35e-15 |  | DFRPTTPGHSPGIGH |  |
| MeTR\_ctg\_74316\_18\_95 | 1 | 5.35e-15 |  | DFRPTTPGHSPGIGH |  |
| LeAL\_scf\_72\_10060\_123 | 1 | 5.35e-15 |  | DFRPTTPGHSPGIGH |  |
| EuSA\_scf\_6\_1153\_108 | 1 | 5.35e-15 |  | DFRPTTPGHSPGIGH |  |
| CiAR.Ka\_chr\_7\_28012\_70 | 1 | 5.35e-15 |  | DFRPTTPGHSPGIGH |  |
| CiAR.Ka\_chr\_7\_27960\_134 | 1 | 5.35e-15 |  | DFRPTTPGHSPGIGH |  |
| BrRA\_chr\_02\_76900\_109 | 1 | 5.35e-15 |  | DFRPTTPGHSPGIGH |  |
| ArTH\_chr\_5\_82442\_124 | 1 | 5.35e-15 |  | DFRPTTPGHSPGIGH |  |
| ArLY\_scf\_8\_63492\_101 | 1 | 5.35e-15 |  | DFRPTTPGHSPGIGH |  |
| AeAR\_scf\_8\_9481\_99 | 1 | 5.35e-15 |  | DFRPTTPGHSPGIGH |  |
| TaHA\_scf\_33\_10698\_96 | 1 | 6.63e-15 |  | DFRPTAPGHSPGIGH |  |
| TaHA\_scf\_10\_9\_81 | 1 | 6.63e-15 |  | DFRPTAPGHSPGIGH |  |
| CuSA\_chr\_2\_34874\_112 | 1 | 6.63e-15 |  | DFRPTAPGHSPGIGH |  |
| CuME\_chr\_11\_2084\_115 | 1 | 6.63e-15 |  | DFRPTAPGHSPGIGH |  |
| CiLA\_chr\_2\_56957\_121 | 1 | 6.63e-15 |  | DFRPTAPGHSPGIGH |  |
| CaSA.Fi\_ctg\_5361018\_3\_115 | 1 | 6.63e-15 |  | DFRPTAPGHSPGIGH |  |
| CaRU\_scf\_4\_3263\_107 | 1 | 6.63e-15 |  | AFRPTEPGHSPGVGH |  |
| AqCO\_scf\_15\_2715\_140 | 1 | 6.63e-15 |  | DFRPTAPGHSPGIGH |  |
| ViAN\_scf\_2025\_131\_125 | 1 | 9.87e-15 |  | DFRPTTPGHSPGVGH |  |
| ThCA.Ma\_chr\_3\_92411\_77 | 1 | 9.87e-15 |  | AFRPTSPGHSPGVGH |  |
| ThCA.Cr\_chr\_03\_68864\_77 | 1 | 9.87e-15 |  | AFRPTSPGHSPGVGH |  |
| PrPE\_scf\_7\_52280\_197 | 1 | 9.87e-15 |  | DFRPTTPGHSPGVGH |  |
| PrPE\_scf\_5\_119216\_70 | 1 | 9.87e-15 |  | AFRPTSPGHSPGVGH |  |
| PrMU\_chr\_8\_31271\_121 | 1 | 9.87e-15 |  | DFRPTTPGHSPGVGH |  |
| PrMU\_chr\_7\_107295\_69 | 1 | 9.87e-15 |  | AFRPTSPGHSPGVGH |  |
| PoTR\_chr\_05\_109127\_118 | 1 | 9.87e-15 |  | DFRPTTPGHSPGVGH |  |
| PoEU\_scf\_234.1\_139\_128 | 1 | 9.87e-15 |  | DFRPTTPGHSPGVGH |  |
| PhVU\_chr\_03\_153596\_84 | 1 | 9.87e-15 |  | DFRPTTPGHSPGVGH |  |
| PhVU\_chr\_03\_136183\_78 | 1 | 9.87e-15 |  | AFRPTSPGHSPGVGH |  |
| PhVU\_chr\_03\_128143\_135 | 1 | 9.87e-15 |  | DFRPTTPGHSPGVGH |  |
| PhVU\_chr\_02\_189930\_69 | 1 | 9.87e-15 |  | AFRPTSPGHSPGVGH |  |
| NeNU\_scf\_00003\_56181\_143 | 1 | 9.87e-15 |  | DFRPTTPGHSPGVGH |  |
| MeTR\_chr\_8\_74208\_73 | 1 | 9.87e-15 |  | AFRPTSPGHSPGVGH |  |
| MeTR\_chr\_5\_197996\_73 | 1 | 9.87e-15 |  | AFRPTSPGHSPGVGH |  |
| MaES\_scf\_01179\_505\_71 | 1 | 9.87e-15 |  | AFRPTSPGHSPGVGH |  |
| MaES\_chr\_xii\_50581\_89 | 1 | 9.87e-15 |  | AFRPTSPGHSPGVGH |  |
| LuAN\_scf\_80590\_1\_7\_98 | 1 | 9.87e-15 |  | DFRPTTPGHSPGVGH |  |
| LuAN\_scf\_79499\_72\_77 | 1 | 9.87e-15 |  | AFRPTSPGHSPGVGH |  |
| LuAN\_scf\_51909\_121\_70 | 1 | 9.87e-15 |  | AFRPTSPGHSPGVGH |  |
| LuAN\_scf\_35677\_71\_92 | 1 | 9.87e-15 |  | AFRPTSPGHSPGVGH |  |
| LiUS\_scf\_488\_1504\_149 | 1 | 9.87e-15 |  | AFRPTSPGHSPGVGH |  |
| LaSI\_ctg\_303226\_5\_88 | 1 | 9.87e-15 |  | AFRPTSPGHSPGVGH |  |
| JaCU\_scf\_14397\_13\_80 | 1 | 9.87e-15 |  | AFRPTSPGHSPGVGH |  |
| JaCU\_scf\_12633\_34\_103 | 1 | 9.87e-15 |  | DFRPTTPGHSPGVGH |  |
| GlMA\_chr\_17\_185246\_118 | 1 | 9.87e-15 |  | DFRPTTPGHSPGVGH |  |
| GlMA\_chr\_16\_157295\_74 | 1 | 9.87e-15 |  | AFRPTSPGHSPGVGH |  |
| GlMA\_chr\_09\_118964\_74 | 1 | 9.87e-15 |  | AFRPTSPGHSPGVGH |  |
| GlMA\_chr\_05\_55563\_127 | 1 | 9.87e-15 |  | DFRPTTPGHSPGVGH |  |
| GlMA\_chr\_01\_142070\_103 | 1 | 9.87e-15 |  | AFRPTSPGHSPGVGH |  |
| FrVE\_lg\_6\_109525\_119 | 1 | 9.87e-15 |  | DFRPTTPGHSPGVGH |  |
| FrVE\_lg\_5\_134785\_87 | 1 | 9.87e-15 |  | AFRPTSPGHSPGVGH |  |
| FrNU\_scf\_00014410.1\_5\_84 | 1 | 9.87e-15 |  | AFRPTSPGHSPGVGH |  |
| FrNI\_scf\_00040543.1\_3\_141 | 1 | 9.87e-15 |  | AFRPTSPGHSPGVGH |  |
| FrII\_scf\_00065470.1\_27\_141 | 1 | 9.87e-15 |  | AFRPTSPGHSPGVGH |  |
| EuGR\_scf\_8\_418792\_178 | 1 | 9.87e-15 |  | DFRPTTPGHSPGVGH |  |
| EuGR\_scf\_5\_333963\_92 | 1 | 9.87e-15 |  | AFRPTSPGHSPGVGH |  |
| EuGR\_scf\_5\_333963\_312 | 1 | 9.87e-15 |  | AFRPTSPGHSPGVGH |  |
| EuGR\_scf\_5\_333963\_290 | 1 | 9.87e-15 |  | AFRPTSPGHSPGVGH |  |
| EuGR\_scf\_5\_333963\_268 | 1 | 9.87e-15 |  | AFRPTSPGHSPGVGH |  |
| EuGR\_scf\_5\_333963\_246 | 1 | 9.87e-15 |  | AFRPTSPGHSPGVGH |  |
| EuGR\_scf\_5\_333963\_224 | 1 | 9.87e-15 |  | AFRPTSPGHSPGVGH |  |
| EuGR\_scf\_5\_333963\_202 | 1 | 9.87e-15 |  | AFRPTSPGHSPGVGH |  |
| EuGR\_scf\_5\_333963\_180 | 1 | 9.87e-15 |  | AFRPTSPGHSPGVGH |  |
| EuGR\_scf\_5\_333963\_158 | 1 | 9.87e-15 |  | AFRPTSPGHSPGVGH |  |
| EuGR\_scf\_5\_333963\_136 | 1 | 9.87e-15 |  | AFRPTSPGHSPGVGH |  |
| EuGR\_scf\_5\_333937\_92 | 1 | 9.87e-15 |  | AFRPTSPGHSPGVGH |  |
| EuGR\_scf\_5\_333937\_246 | 1 | 9.87e-15 |  | AFRPTSPGHSPGVGH |  |
| EuGR\_scf\_5\_333937\_224 | 1 | 9.87e-15 |  | AFRPTSPGHSPGVGH |  |
| EuGR\_scf\_5\_333937\_180 | 1 | 9.87e-15 |  | AFRPTSPGHSPGVGH |  |
| EuGR\_scf\_5\_333937\_136 | 1 | 9.87e-15 |  | AFRPTSPGHSPGVGH |  |
| EuGR\_scf\_5\_333937\_114 | 1 | 9.87e-15 |  | AFRPTSPGHSPGVGH |  |
| EuGR\_scf\_5\_333720\_174 | 1 | 9.87e-15 |  | AFRPTSPGHSPGVGH |  |
| EuGR\_scf\_5\_238372\_290 | 1 | 9.87e-15 |  | AFRPTSPGHSPGVGH |  |
| EuGR\_scf\_5\_238372\_268 | 1 | 9.87e-15 |  | AFRPTSPGHSPGVGH |  |
| EuGR\_scf\_5\_238372\_246 | 1 | 9.87e-15 |  | AFRPTSPGHSPGVGH |  |
| EuGR\_scf\_5\_238372\_224 | 1 | 9.87e-15 |  | AFRPTSPGHSPGVGH |  |
| EuGR\_scf\_5\_238372\_202 | 1 | 9.87e-15 |  | AFRPTSPGHSPGVGH |  |
| EuGR\_scf\_5\_238372\_180 | 1 | 9.87e-15 |  | AFRPTSPGHSPGVGH |  |
| EuGR\_scf\_5\_238235\_75 | 1 | 9.87e-15 |  | AFRPTSPGHSPGVGH |  |
| EuGR\_scf\_5\_238225\_97 | 1 | 9.87e-15 |  | AFRPTSPGHSPGVGH |  |
| EuGR\_scf\_5\_238225\_75 | 1 | 9.87e-15 |  | AFRPTSPGHSPGVGH |  |
| EuGR\_scf\_5\_238225\_141 | 1 | 9.87e-15 |  | AFRPTSPGHSPGVGH |  |
| EuGR\_scf\_5\_238225\_119 | 1 | 9.87e-15 |  | AFRPTSPGHSPGVGH |  |
| EuGR\_scf\_4\_135215\_83 | 1 | 9.87e-15 |  | AFRPTSPGHSPGVGH |  |
| EuCA\_ctg\_023562\_313\_178 | 1 | 9.87e-15 |  | DFRPTTPGHSPGVGH |  |
| EuCA\_ctg\_023070\_58\_92 | 1 | 9.87e-15 |  | AFRPTSPGHSPGVGH |  |
| EuCA\_ctg\_023070\_58\_290 | 1 | 9.87e-15 |  | AFRPTSPGHSPGVGH |  |
| EuCA\_ctg\_023070\_58\_268 | 1 | 9.87e-15 |  | AFRPTSPGHSPGVGH |  |
| EuCA\_ctg\_023070\_58\_202 | 1 | 9.87e-15 |  | AFRPTSPGHSPGVGH |  |
| EuCA\_ctg\_023070\_58\_158 | 1 | 9.87e-15 |  | AFRPTSPGHSPGVGH |  |
| EuCA\_ctg\_023070\_58\_114 | 1 | 9.87e-15 |  | AFRPTSPGHSPGVGH |  |
| EuCA\_ctg\_018397\_212\_75 | 1 | 9.87e-15 |  | AFRPTSPGHSPGVGH |  |
| EuCA\_ctg\_018397\_20\_173 | 1 | 9.87e-15 |  | AFRPTSPGHSPGVGH |  |
| EuCA\_ctg\_011241\_14\_93 | 1 | 9.87e-15 |  | AFRPTSPGHSPGVGH |  |
| EuCA\_ctg\_010936\_1\_114 | 1 | 9.87e-15 |  | AFRPTSPGHSPGVGH |  |
| CuSA\_chr\_6\_94274\_95 | 1 | 9.87e-15 |  | DFRPTTPGHSPGVGH |  |
| CuSA\_chr\_4\_27432\_88 | 1 | 9.87e-15 |  | AFRPTSPGHSPGVGH |  |
| CuME\_chr\_08\_112716\_95 | 1 | 9.87e-15 |  | DFRPTTPGHSPGVGH |  |
| CuME\_chr\_07\_17885\_102 | 1 | 9.87e-15 |  | AFRPTSPGHSPGVGH |  |
| CiSI\_scf\_00028\_3838\_84 | 1 | 9.87e-15 |  | AFRPTSPGHSPGVGH |  |
| CiLA\_chr\_1\_108332\_117 | 1 | 9.87e-15 |  | DFRPTTPGHSPGVGH |  |
| CiLA\_chr\_11\_92040\_97 | 1 | 9.87e-15 |  | AFRPTSPGHSPGVGH |  |
| CiCL\_scf\_2\_23313\_84 | 1 | 9.87e-15 |  | AFRPTSPGHSPGVGH |  |
| CiAR.Ka\_chr\_8\_49897\_98 | 1 | 9.87e-15 |  | AFRPTSPGHSPGVGH |  |
| CiAR.De\_lg\_3\_44114\_98 | 1 | 9.87e-15 |  | AFRPTSPGHSPGVGH |  |
| CaPA\_ctg\_55\_3048\_126 | 1 | 9.87e-15 |  | DFRPTTPGHSPGVGH |  |
| CaCA\_scf\_133302\_238\_87 | 1 | 9.87e-15 |  | DFRPTTPGHSPGVGH |  |
| CaCA\_lg\_11\_231024\_76 | 1 | 9.87e-15 |  | AFRPTSPGHSPGVGH |  |
| CaCA\_lg\_10\_34423\_105 | 1 | 9.87e-15 |  | DFRPTTPGHSPGVGH |  |
| CaCA\_lg\_06\_45397\_123 | 1 | 9.87e-15 |  | AFRPTSPGHSPGVGH |  |
| BeNA\_scf\_7799\_40\_74 | 1 | 9.87e-15 |  | AFRPTSPGHSPGVGH |  |
| BeNA\_scf\_47625\_4\_80 | 1 | 9.87e-15 |  | AFRPTSPGHSPGVGH |  |
| BeNA\_scf\_14434\_133\_110 | 1 | 9.87e-15 |  | DFRPTTPGHSPGVGH |  |
| AzIN\_scf\_4996\_1\_8\_83 | 1 | 9.87e-15 |  | AFRPTSPGHSPGVGH |  |
| AzIN\_scf\_23221\_3\_16\_83 | 1 | 9.87e-15 |  | AFRPTSPGHSPGVGH |  |
| ArIP\_chr\_05\_24681\_129 | 1 | 9.87e-15 |  | AFRPTSPGHSPGVGH |  |
| ThCA.Ma\_chr\_1\_202475\_156 | 1 | 1.10e-14 |  | DFRPTNPGHSPGVGH |  |
| ThCA.Cr\_chr\_00\_28530\_156 | 1 | 1.10e-14 |  | DFRPTNPGHSPGVGH |  |
| ThCA.Ma\_chr\_1\_202461\_153 | 1 | 1.26e-14 |  | DFRPTAPGHSPGVGH |  |
| ThCA.Cr\_chr\_01\_161924\_153 | 1 | 1.26e-14 |  | DFRPTAPGHSPGVGH |  |
| TaHA\_scf\_10\_4\_230 | 1 | 1.26e-14 |  | AFRPTTPGHSPGIGH |  |
| RiCO\_ctg\_29676\_82\_137 | 1 | 1.26e-14 |  | DFRPTAPGHSPGVGH |  |
| RiCO\_ctg\_29676\_38\_114 | 1 | 1.26e-14 |  | DFRPTAPGHSPGVGH |  |
| PyCO\_scf\_19577\_8\_112 | 1 | 1.26e-14 |  | DFRPTAPGHSPGVGH |  |
| PyBR\_scf\_520.0\_2966\_132 | 1 | 1.26e-14 |  | DFRPTAPGHSPGVGH |  |
| PyBR\_scf\_520.0\_2920\_105 | 1 | 1.26e-14 |  | DFRPTAPGHSPGVGH |  |
| PyBR\_scf\_4.0\_9723\_112 | 1 | 1.26e-14 |  | DFRPTAPGHSPGVGH |  |
| PyBR\_scf\_197.0\_2336\_118 | 1 | 1.26e-14 |  | DFRPTAPGHSPGVGH |  |
| PyBR\_scf\_197.0\_2325\_105 | 1 | 1.26e-14 |  | DFRPTAPGHSPGVGH |  |
| PyBR\_scf\_145.0\_93\_102 | 1 | 1.26e-14 |  | DFRPTAPGHSPGVGH |  |
| PrPE\_scf\_7\_52240\_107 | 1 | 1.26e-14 |  | DFRPTAPGHSPGVGH |  |
| PoTR\_chr\_07\_72718\_128 | 1 | 1.26e-14 |  | DFRPTAPGHSPGVGH |  |
| PoTR\_chr\_07\_72626\_128 | 1 | 1.26e-14 |  | DFRPTAPGHSPGVGH |  |
| PoEU\_scf\_354.1\_775\_121 | 1 | 1.26e-14 |  | DFRPTAPGHSPGVGH |  |
| MoNO\_scf\_123\_489\_89 | 1 | 1.26e-14 |  | DFRPTAPGHSPGVGH |  |
| MoNO\_scf\_123\_420\_102 | 1 | 1.26e-14 |  | DFRPTAPGHSPGVGH |  |
| MeTR\_scf\_ac233112.6\_194\_63 | 1 | 1.26e-14 |  | AFRPTTPGHSPGIGH |  |
| MaES\_chr\_xviii\_47367\_103 | 1 | 1.26e-14 |  | DFRPTAPGHSPGVGH |  |
| MaES\_chr\_viii\_35996\_108 | 1 | 1.26e-14 |  | DFRPTAPGHSPGVGH |  |
| MaDO\_chr\_15\_179568\_115 | 1 | 1.26e-14 |  | DFRPTAPGHSPGVGH |  |
| LiUS\_scf\_2416\_608\_97 | 1 | 1.26e-14 |  | DFRPTAPGHSPGVGH |  |
| LiUS\_scf\_1249\_1864\_70 | 1 | 1.26e-14 |  | DFRPTAPGHSPGVGH |  |
| HeBR\_scf\_155851\_143\_131 | 1 | 1.26e-14 |  | DFRPTAPGHSPGVGH |  |
| GoRA\_chr\_04\_120962\_88 | 1 | 1.26e-14 |  | AFRPTTPGHSPGIGH |  |
| GoRA\_chr\_03\_227227\_82 | 1 | 1.26e-14 |  | DFRPTAPGHSPGVGH |  |
| GoRA\_chr\_03\_227128\_82 | 1 | 1.26e-14 |  | DFRPTAPGHSPGVGH |  |
| GoRA\_chr\_03\_227124\_82 | 1 | 1.26e-14 |  | DFRPTAPGHSPGVGH |  |
| FrVE\_lg\_6\_109489\_114 | 1 | 1.26e-14 |  | DFRPTAPGHSPGVGH |  |
| FrNU\_scf\_00078196.1\_20\_114 | 1 | 1.26e-14 |  | DFRPTAPGHSPGVGH |  |
| FrNI\_scf\_00104625.1\_5\_114 | 1 | 1.26e-14 |  | DFRPTAPGHSPGVGH |  |
| FrII\_scf\_00001632.1\_18\_107 | 1 | 1.26e-14 |  | DFRPTAPGHSPGVGH |  |
| FrEX\_scf\_5423\_91\_92 | 1 | 1.26e-14 |  | AFRPTTPGHSPGIGH |  |
| FrEX\_scf\_5423\_116\_91 | 1 | 1.26e-14 |  | DFRPTEPGHSPGMGH |  |
| FrEX\_scf\_3900\_232\_91 | 1 | 1.26e-14 |  | DFRPTEPGHSPGMGH |  |
| EuGR\_scf\_9\_63899\_121 | 1 | 1.26e-14 |  | DFRPTAPGHSPGVGH |  |
| EuGR\_scf\_9\_63713\_93 | 1 | 1.26e-14 |  | DFRPTAPGHSPGVGH |  |
| EuGR\_scf\_6\_193456\_107 | 1 | 1.26e-14 |  | DFRPTAPGHSPGVGH |  |
| EuGR\_scf\_6\_193261\_122 | 1 | 1.26e-14 |  | DFRPTAPGHSPGVGH |  |
| EuGR\_scf\_6\_193234\_113 | 1 | 1.26e-14 |  | DFRPTAPGHSPGVGH |  |
| EuCA\_ctg\_025488\_47\_131 | 1 | 1.26e-14 |  | DFRPTAPGHSPGVGH |  |
| EuCA\_ctg\_005169\_46\_147 | 1 | 1.26e-14 |  | DFRPTAPGHSPGVGH |  |
| EuCA\_ctg\_004830\_158\_147 | 1 | 1.26e-14 |  | DFRPTAPGHSPGVGH |  |
| CiSI\_scf\_00006\_4984\_82 | 1 | 1.26e-14 |  | DFRPTAPGHSPGVGH |  |
| CiSI\_scf\_00006\_4933\_148 | 1 | 1.26e-14 |  | DFRPTAPGHSPGVGH |  |
| CiCL\_scf\_7\_106293\_82 | 1 | 1.26e-14 |  | DFRPTAPGHSPGVGH |  |
| CiCL\_scf\_7\_106244\_148 | 1 | 1.26e-14 |  | DFRPTAPGHSPGVGH |  |
| CiAR.Ka\_chr\_7\_28076\_78 | 1 | 1.26e-14 |  | AFRPTTPGHSPGIGH |  |
| CiAR.De\_scf\_04274\_79\_78 | 1 | 1.26e-14 |  | AFRPTTPGHSPGIGH |  |
| BeNA\_scf\_16996\_34\_116 | 1 | 1.26e-14 |  | DFRPTAPGHSPGVGH |  |
| AzIN\_scf\_8125\_2\_25\_123 | 1 | 1.26e-14 |  | DFRPTAPGHSPGVGH |  |
| AzIN\_scf\_42817\_1\_25\_121 | 1 | 1.26e-14 |  | DFRPTAPGHSPGVGH |  |
| AzIN\_scf\_32239\_2\_2\_46 | 1 | 1.26e-14 |  | DFRPTAPGHSPGVGH |  |
| AzIN\_scf\_2708\_2\_45\_98 | 1 | 1.26e-14 |  | DFRPTAPGHSPGVGH |  |
| AzIN\_scf\_2043\_2\_3\_98 | 1 | 1.26e-14 |  | DFRPTAPGHSPGVGH |  |
| AzIN\_scf\_2043\_2\_22\_46 | 1 | 1.26e-14 |  | DFRPTAPGHSPGVGH |  |
| AzIN\_scf\_17876\_1\_40\_121 | 1 | 1.26e-14 |  | DFRPTAPGHSPGVGH |  |
| AzIN\_scf\_17876\_1\_26\_121 | 1 | 1.26e-14 |  | DFRPTAPGHSPGVGH |  |
| ArIP\_chr\_05\_24658\_71 | 1 | 1.26e-14 |  | DFRPTAPGHSPGVGH |  |
| ArDU\_chr\_05\_21700\_71 | 1 | 1.26e-14 |  | DFRPTAPGHSPGVGH |  |
| AqCO\_scf\_15\_2734\_84 | 1 | 1.26e-14 |  | AFRPTTPGHSPGIGH |  |
| LiUS\_scf\_2416\_588\_171 | 1 | 1.33e-14 |  | DFRPTEPGYSPGVGH |  |
| LiUS\_scf\_1249\_1842\_168 | 1 | 1.33e-14 |  | DFRPTEPGYSPGVGH |  |
| NeNU\_scf\_00099\_7001\_128 | 1 | 1.46e-14 |  | AFRPTAPGHSPGIGH |  |
| FrEX\_scf\_8460\_10\_97 | 1 | 1.46e-14 |  | AFRPTAPGHSPGIGH |  |
| FrEX\_scf\_14360\_8\_123 | 1 | 1.46e-14 |  | DFRPTSPGQSPGVGH |  |
| RiCO\_ctg\_29676\_57\_165 | 1 | 1.59e-14 |  | DFRPTSPGYSPGVGH |  |
| JaCU\_scf\_07803\_17\_84 | 1 | 1.59e-14 |  | EFRPTTPGHSPGVGH |  |
| JaCU\_scf\_02121\_40\_249 | 1 | 1.59e-14 |  | DFRPTSPGYSPGVGH |  |
| JaCU\_scf\_02121\_40\_190 | 1 | 1.59e-14 |  | DFRPTSPGYSPGVGH |  |
| JaCU\_scf\_02121\_40\_129 | 1 | 1.59e-14 |  | DFRPTKPGYSPGVGH |  |
| HeBR\_scf\_243691\_91\_88 | 1 | 1.59e-14 |  | DFRPTSPGYSPGVGH |  |
| HeBR\_scf\_155851\_157\_109 | 1 | 1.59e-14 |  | DFRPTSPGYSPGVGH |  |
| EuGR\_scf\_9\_63568\_136 | 1 | 1.59e-14 |  | DFRPTQPGHSPGVGH |  |
| EuGR\_scf\_6\_193319\_146 | 1 | 1.59e-14 |  | DFRPTQPGHSPGVGH |  |
| ViAN\_scf\_2025\_123\_85 | 1 | 1.77e-14 |  | AFRPTTPGHSPGVGH |  |
| ViAN\_ctg\_11585\_0\_25\_93 | 1 | 1.77e-14 |  | AFRPTTPGHSPGVGH |  |
| ThCA.Ma\_chr\_1\_202485\_213 | 1 | 1.77e-14 |  | AFRPTTPGHSPGVGH |  |
| ThCA.Cr\_chr\_01\_161945\_213 | 1 | 1.77e-14 |  | AFRPTTPGHSPGVGH |  |
| PyCO\_scf\_00781\_665\_83 | 1 | 1.77e-14 |  | AFRPTTPGHSPGVGH |  |
| PyBR\_scf\_190.0\_3174\_83 | 1 | 1.77e-14 |  | AFRPTTPGHSPGVGH |  |
| PrPE\_scf\_7\_52257\_80 | 1 | 1.77e-14 |  | AFRPTTPGHSPGVGH |  |
| PrMU\_chr\_8\_31255\_80 | 1 | 1.77e-14 |  | AFRPTTPGHSPGVGH |  |
| PoTR\_scf\_376\_64\_80 | 1 | 1.77e-14 |  | AFRPTTPGHSPGVGH |  |
| PoTR\_chr\_04\_65116\_80 | 1 | 1.77e-14 |  | AFRPTTPGHSPGVGH |  |
| PoEU\_scf\_14.1\_5334\_104 | 1 | 1.77e-14 |  | AFRPTTPGHSPGVGH |  |
| PhVU\_chr\_03\_128129\_94 | 1 | 1.77e-14 |  | AFRPTTPGHSPGVGH |  |
| PhVU\_chr\_02\_5535\_105 | 1 | 1.77e-14 |  | AFRPTTPGHSPGVGH |  |
| MeTR\_chr\_5\_16827\_84 | 1 | 1.77e-14 |  | AFRPTTPGHSPGVGH |  |
| LuAN\_scf\_13935\_13\_67 | 1 | 1.77e-14 |  | AFRPTTPGHSPGVGH |  |
| LaSI\_scf\_25684\_14\_96 | 1 | 1.77e-14 |  | AFRPTTPGHSPGVGH |  |
| GoRA\_chr\_07\_252127\_82 | 1 | 1.77e-14 |  | AFRPTTPGHSPGVGH |  |
| GoRA\_chr\_03\_227267\_75 | 1 | 1.77e-14 |  | AFRPTTPGHSPGVGH |  |
| GlMA\_chr\_17\_185198\_110 | 1 | 1.77e-14 |  | AFRPTTPGHSPGVGH |  |
| GlMA\_chr\_11\_11247\_72 | 1 | 1.77e-14 |  | AFRPTTPGHSPGVGH |  |
| GlMA\_chr\_05\_55551\_80 | 1 | 1.77e-14 |  | AFRPTTPGHSPGVGH |  |
| GlMA\_chr\_01\_174715\_86 | 1 | 1.77e-14 |  | AFRPTTPGHSPGVGH |  |
| FrVE\_lg\_6\_109514\_101 | 1 | 1.77e-14 |  | AFRPTTPGHSPGVGH |  |
| FrNU\_scf\_00093857.1\_6\_101 | 1 | 1.77e-14 |  | AFRPTTPGHSPGVGH |  |
| FrNI\_scf\_00052972.1\_1\_86 | 1 | 1.77e-14 |  | AFRPTTPGHSPGVGH |  |
| FrII\_scf\_00066163.1\_12\_118 | 1 | 1.77e-14 |  | AFRPTTPGHSPGVGH |  |
| CuSA\_chr\_6\_94302\_79 | 1 | 1.77e-14 |  | AFRPTTPGHSPGVGH |  |
| CuME\_chr\_08\_112743\_85 | 1 | 1.77e-14 |  | AFRPTTPGHSPGVGH |  |
| CiSI\_scf\_00006\_4973\_85 | 1 | 1.77e-14 |  | AFRPTTPGHSPGVGH |  |
| CiLA\_chr\_1\_108387\_184 | 1 | 1.77e-14 |  | AFRPTTPGHSPGVGH |  |
| CiCL\_scf\_7\_106282\_89 | 1 | 1.77e-14 |  | AFRPTTPGHSPGVGH |  |
| CaSA.PK\_scf\_63384\_15\_68 | 1 | 1.77e-14 |  | AFRPTTPGHSPGVGH |  |
| CaSA.PK\_scf\_47871\_7\_92 | 1 | 1.77e-14 |  | AFRPTTPGHSPGVGH |  |
| CaSA.Fi\_ctg\_14483977\_3\_68 | 1 | 1.77e-14 |  | AFRPTTPGHSPGVGH |  |
| CaSA.Fi\_ctg\_14449324\_3\_108 | 1 | 1.77e-14 |  | AFRPTTPGHSPGVGH |  |
| CaRU\_scf\_1\_50368\_115 | 1 | 1.77e-14 |  | DFRPTSPGNSPGVGH |  |
| CaPA\_ctg\_55\_3035\_58 | 1 | 1.77e-14 |  | AFRPTTPGHSPGVGH |  |
| CaCA\_lg\_10\_34412\_108 | 1 | 1.77e-14 |  | AFRPTTPGHSPGVGH |  |
| CaCA\_lg\_06\_45387\_73 | 1 | 1.77e-14 |  | AFRPTTPGHSPGVGH |  |
| BeNA\_scf\_16996\_46\_81 | 1 | 1.77e-14 |  | AFRPTTPGHSPGVGH |  |
| AzIN\_scf\_42817\_1\_18\_125 | 1 | 1.77e-14 |  | AFRPTTPGHSPGVGH |  |
| AzIN\_scf\_17876\_1\_33\_125 | 1 | 1.77e-14 |  | AFRPTTPGHSPGVGH |  |
| ArIP\_chr\_05\_897879\_80 | 1 | 1.77e-14 |  | AFRPTTPGHSPGVGH |  |
| ArDU\_chr\_05\_606935\_76 | 1 | 1.77e-14 |  | AFRPTTPGHSPGVGH |  |
| RiCO\_ctg\_29676\_74\_79 | 1 | 2.02e-14 |  | AFRPTNPGHSPGVGH |  |
| PoTR\_chr\_07\_72919\_117 | 1 | 2.02e-14 |  | AFRPTNPGHSPGVGH |  |
| PoEU\_scf\_234.1\_307\_97 | 1 | 2.02e-14 |  | AFRPTNPGHSPGVGH |  |
| MoNO\_scf\_123\_476\_102 | 1 | 2.02e-14 |  | AFRPTNPGHSPGVGH |  |
| MaES\_chr\_viii\_75422\_87 | 1 | 2.02e-14 |  | AFRPTNPGHSPGVGH |  |
| JaCU\_scf\_19785\_4\_77 | 1 | 2.02e-14 |  | AFRPTNPGHSPGVGH |  |
| AmTR\_scf\_00157\_241\_75 | 1 | 2.02e-14 |  | NFRPTSPGHSPGIGH |  |
| SiIR\_scf\_8\_11159\_61 | 1 | 2.35e-14 |  | AFRPTKPGNSPGIGH |  |
| ScPA\_chr\_4-2\_333\_105 | 1 | 2.35e-14 |  | AFRPTAPGHSPGVGH |  |
| ScPA\_chr\_2-4\_32077\_91 | 1 | 2.35e-14 |  | AFRPTKPGNSPGIGH |  |
| MoNO\_scf\_256\_2508\_89 | 1 | 2.35e-14 |  | AFRPTAPGHSPGVGH |  |
| FrEX\_scf\_7878\_74\_73 | 1 | 2.35e-14 |  | DFRPTTPGNSPGIGH |  |
| EuSA\_scf\_6\_1171\_90 | 1 | 2.35e-14 |  | AFRPTKPGNSPGIGH |  |
| EuSA\_scf\_15\_6297\_98 | 1 | 2.35e-14 |  | AFRPTAPGHSPGVGH |  |
| CaRU\_scf\_8\_42181\_67 | 1 | 2.35e-14 |  | AFRPTKPGNSPGIGH |  |
| BrRA\_chr\_09\_208498\_68 | 1 | 2.35e-14 |  | AFRPTKPGNSPGIGH |  |
| BrRA\_chr\_07\_115031\_67 | 1 | 2.35e-14 |  | AFRPTKPGNSPGIGH |  |
| ArLY\_scf\_4\_5700\_92 | 1 | 2.35e-14 |  | AFRPTAPGHSPGVGH |  |
| ArIP\_chr\_05\_24674\_60 | 1 | 2.35e-14 |  | AFRPTAPGHSPGVGH |  |
| ArDU\_chr\_05\_21712\_77 | 1 | 2.35e-14 |  | AFRPTAPGHSPGVGH |  |
| ArDU\_chr\_01\_22525\_80 | 1 | 2.35e-14 |  | AFRPTAPGHSPGVGH |  |
| LuAN\_scf\_92387\_1\_2\_84 | 1 | 2.50e-14 |  | NFRPTEPGHSPGVGH |  |
| FrEX\_scf\_233\_540\_103 | 1 | 2.50e-14 |  | DFRPTNPGNSPGIGH |  |
| TaHA\_scf\_33\_10708\_113 | 1 | 3.02e-14 |  | DFKPTSPGHSPGVGH |  |
| GlMA\_chr\_11\_11233\_165 | 1 | 3.02e-14 |  | DFRPTDPGHSPGVGH |  |
| ArIP\_chr\_01\_784210\_91 | 1 | 3.02e-14 |  | DFRPTDPGHSPGVGH |  |
| ArDU\_chr\_01\_262\_90 | 1 | 3.02e-14 |  | DFRPTDPGHSPGVGH |  |
| AmTR\_scf\_00270\_48\_202 | 1 | 3.02e-14 |  | DFRPTDPGHSPGVGH |  |
| AmTR\_scf\_00270\_48\_175 | 1 | 3.02e-14 |  | DFRPTDPGHSPGVGH |  |
| AmTR\_scf\_00270\_119\_90 | 1 | 3.02e-14 |  | DFRPTDPGHSPGVGH |  |
| AmTR\_scf\_00240\_313\_90 | 1 | 3.02e-14 |  | DFRPTDPGHSPGVGH |  |
| AmTR\_scf\_00240\_313\_117 | 1 | 3.02e-14 |  | DFRPTDPGHSPGVGH |  |
| AeAR\_scf\_22\_2\_103 | 1 | 3.02e-14 |  | DFRPTNPGHSPGMGH |  |
| ThCA.Ma\_chr\_1\_202475\_111 | 1 | 3.35e-14 |  | DFRPTTPGNSPGVGH |  |
| ThCA.Cr\_chr\_00\_28530\_111 | 1 | 3.35e-14 |  | DFRPTTPGNSPGVGH |  |
| PyCO\_scf\_03185\_22\_100 | 1 | 3.35e-14 |  | DFRPTTPGNSPGVGH |  |
| PrPE\_scf\_7\_52252\_138 | 1 | 3.35e-14 |  | DFRPTTPGNSPGVGH |  |
| PrMU\_chr\_8\_31248\_99 | 1 | 3.35e-14 |  | DFRPTTPGNSPGVGH |  |
| GoRA\_chr\_03\_4174\_104 | 1 | 3.35e-14 |  | DFRPTTPGNSPGVGH |  |
| GoRA\_chr\_03\_227308\_104 | 1 | 3.35e-14 |  | DFRPTTPGNSPGVGH |  |
| GoRA\_chr\_03\_227254\_104 | 1 | 3.35e-14 |  | DFRPTTPGNSPGVGH |  |
| GoRA\_chr\_03\_227048\_78 | 1 | 3.35e-14 |  | DFRPTTPGNSPGVGH |  |
| CuSA\_chr\_2\_34874\_70 | 1 | 3.35e-14 |  | DFRPTTPGNSPGVGH |  |
| CuME\_chr\_11\_2084\_73 | 1 | 3.35e-14 |  | DFRPTTPGNSPGVGH |  |
| CiSI\_scf\_00006\_4959\_95 | 1 | 3.35e-14 |  | DFRPTTPGNSPGVGH |  |
| CiLA\_chr\_2\_56957\_84 | 1 | 3.35e-14 |  | DFRPTTPGNSPGVGH |  |
| CiCL\_scf\_7\_106263\_95 | 1 | 3.35e-14 |  | DFRPTTPGNSPGVGH |  |
| BeNA\_scf\_14434\_160\_88 | 1 | 3.35e-14 |  | DFRPTTPGNSPGVGH |  |
| AzIN\_scf\_17876\_1\_26\_75 | 1 | 3.35e-14 |  | DFRPTTPGNSPGVGH |  |
| AqCO\_scf\_63\_6175\_77 | 1 | 3.35e-14 |  | AFRPTSPGNSPGVGH |  |
| ScPA\_chr\_5-1\_14490\_221 | 1 | 3.64e-14 |  | DFKPTTPGHSPGIGH |  |
| ScPA\_chr\_1-2\_6237\_71 | 1 | 3.64e-14 |  | DFRPTNPGNSPGVGH |  |
| NeNU\_scf\_00003\_56217\_113 | 1 | 3.64e-14 |  | AFRPTDPGHSPGIGH |  |
| LeAL\_scf\_30\_1513\_76 | 1 | 3.64e-14 |  | EFRPTTPGNSPGIGH |  |
| JaCU\_scf\_02121\_40\_308 | 1 | 3.64e-14 |  | DFRPTAPGYSPGVGH |  |
| EuSA\_scf\_7\_63503\_123 | 1 | 3.64e-14 |  | DFRPTNPGNSPGVGH |  |
| EuSA\_scf\_16\_7797\_218 | 1 | 3.64e-14 |  | DFKPTTPGHSPGIGH |  |
| BrRA\_chr\_01\_110059\_174 | 1 | 3.64e-14 |  | DFKPTTPGHSPGIGH |  |
| ArTH\_chr\_5\_82456\_80 | 1 | 3.64e-14 |  | EFRPTTPGNSPGIGH |  |
| ArTH\_chr\_1\_53133\_134 | 1 | 3.64e-14 |  | DFRPTNPGNSPGVGH |  |
| ArLY\_scf\_8\_63513\_83 | 1 | 3.64e-14 |  | EFRPTTPGNSPGIGH |  |
| ArLY\_scf\_1\_64585\_88 | 1 | 3.64e-14 |  | DFRPTNPGNSPGVGH |  |
| AeAR\_scf\_8\_9495\_67 | 1 | 3.64e-14 |  | EFRPTTPGNSPGIGH |  |
| AeAR\_scf\_2092\_3\_67 | 1 | 3.64e-14 |  | DFRPTNPGNSPGVGH |  |
| TaHA\_scf\_12\_14888\_70 | 1 | 4.15e-14 |  | DFRPTAPGNSPGVGH |  |
| SiIR\_scf\_8\_11167\_81 | 1 | 4.54e-14 |  | QFRPTTPGNSPGIGH |  |
| PyCO\_scf\_00343\_1191\_86 | 1 | 4.54e-14 |  | AFRPTSPGHSPGVGN |  |
| PyBR\_scf\_136.0\_4568\_83 | 1 | 4.54e-14 |  | AFRPTSPGHSPGVGN |  |
| LeAL\_scf\_72\_10087\_66 | 1 | 4.54e-14 |  | AFRPTNPGNSPGIGH |  |
| LeAL\_scf\_30\_1510\_69 | 1 | 4.54e-14 |  | AFRPTNPGNSPGIGH |  |
| EuSA\_scf\_6\_1181\_107 | 1 | 4.54e-14 |  | QFRPTTPGNSPGIGH |  |
| EuGR\_scf\_5\_333937\_202 | 1 | 4.54e-14 |  | AFRPTSPGHSPGVGN |  |
| EuGR\_scf\_5\_333720\_196 | 1 | 4.54e-14 |  | AFRPTSPGHSPGVGN |  |
| EuCA\_scf\_629621\_2\_174 | 1 | 4.54e-14 |  | AFRPTSPGHSPGVGN |  |
| EuCA\_ctg\_018397\_20\_195 | 1 | 4.54e-14 |  | AFRPTSPGHSPGVGN |  |
| BrRA\_chr\_07\_115039\_106 | 1 | 4.54e-14 |  | QFRPTTPGNSPGIGH |  |
| ArTH\_chr\_5\_82453\_67 | 1 | 4.54e-14 |  | AFRPTNPGNSPGIGH |  |
| ArLY\_scf\_8\_63506\_67 | 1 | 4.54e-14 |  | AFRPTNPGNSPGIGH |  |
| AeAR\_scf\_8\_9492\_72 | 1 | 4.54e-14 |  | AFRPTNPGNSPGIGH |  |
| AqCO\_scf\_15\_2726\_82 | 1 | 4.94e-14 |  | DYRPTAPGHSPGIGH |  |
| SiIR\_scf\_20\_535\_232 | 1 | 5.34e-14 |  | DFKPTTPGHSPGVGH |  |
| ScPA\_chr\_2-4\_32069\_96 | 1 | 5.34e-14 |  | DFKPTTPGHSPGVGH |  |
| EuSA\_scf\_6\_1164\_87 | 1 | 5.34e-14 |  | DFKPTTPGHSPGVGH |  |
| CaPA\_ctg\_64\_5934\_76 | 1 | 5.34e-14 |  | NFRPTTPGHSPGVGH |  |
| BrRA\_chr\_09\_146618\_227 | 1 | 5.34e-14 |  | DFKPTTPGHSPGVGH |  |
| AzIN\_scf\_2708\_1\_18\_131 | 1 | 5.34e-14 |  | DFRPTAPGHSPGVGN |  |
| ArTH\_chr\_3\_89083\_252 | 1 | 5.34e-14 |  | DFKPTTPGHSPGVGH |  |
| ArLY\_scf\_5\_79011\_239 | 1 | 5.34e-14 |  | DFKPTTPGHSPGVGH |  |
| ViVI\_chr\_2\_50414\_78 | 1 | 5.93e-14 |  | AYRPTSPGHSPGVGH |  |
| ViAN\_scf\_11539\_37\_63 | 1 | 5.93e-14 |  | AYRPTSPGHSPGVGH |  |
| ViAN\_ctg\_11418\_0\_91\_73 | 1 | 5.93e-14 |  | AYRPTSPGHSPGVGH |  |
| SeIN\_lg\_1\_108320\_81 | 1 | 5.93e-14 |  | AFRPTAPGHSPGMGH |  |
| PiTA\_scf\_306645\_59\_149 | 1 | 5.93e-14 |  | AFRPTSSGHSPGVGH |  |
| PiTA\_scf\_184314\_493\_135 | 1 | 5.93e-14 |  | AFRPTSSGHSPGVGH |  |
| PhVU\_chr\_03\_128057\_103 | 1 | 5.93e-14 |  | AFRPTTPGNSPGVGH |  |
| NeNU\_scf\_00096\_5431\_74 | 1 | 5.93e-14 |  | AFRPTAPGHSPGMGH |  |
| MeTR\_scf\_ac233112.6\_165\_83 | 1 | 5.93e-14 |  | AFRPTTPGNSPGVGH |  |
| LuAN\_scf\_76000\_38\_68 | 1 | 5.93e-14 |  | AFRPTTPGNSPGVGH |  |
| LuAN\_scf\_65780\_36\_67 | 1 | 5.93e-14 |  | AFRPTTPGNSPGVGH |  |
| LuAN\_scf\_18977\_68\_69 | 1 | 5.93e-14 |  | AFRPTTPGNSPGVGH |  |
| LuAN\_scf\_14424\_14\_163 | 1 | 5.93e-14 |  | DFKPTNPGHSPGVGH |  |
| LiUS\_scf\_66\_415\_106 | 1 | 5.93e-14 |  | AYRPTSPGHSPGVGH |  |
| LeAL\_scf\_217\_537\_170 | 1 | 5.93e-14 |  | EFKPTTPGHSPGIGH |  |
| KaFE\_scf\_1373279\_129\_112 | 1 | 5.93e-14 |  | AFRPTSSGHSPGVGH |  |
| KaFE\_scf\_1366140\_229\_67 | 1 | 5.93e-14 |  | AFRPTSSGHSPGVGH |  |
| HeBR\_scf\_114295\_32\_84 | 1 | 5.93e-14 |  | AYRPTSPGHSPGVGH |  |
| GlMA\_chr\_17\_184876\_120 | 1 | 5.93e-14 |  | AFRPTTPGNSPGVGH |  |
| GlMA\_chr\_17\_184560\_114 | 1 | 5.93e-14 |  | AFRPTTPGNSPGVGH |  |
| GlMA\_chr\_16\_156497\_78 | 1 | 5.93e-14 |  | AYRPTSPGHSPGVGH |  |
| GlMA\_chr\_11\_11233\_116 | 1 | 5.93e-14 |  | AFRPTTPGNSPGVGH |  |
| GlMA\_chr\_01\_174694\_98 | 1 | 5.93e-14 |  | AFRPTTPGNSPGVGH |  |
| CaRU\_scf\_5\_58226\_229 | 1 | 5.93e-14 |  | EFKPTTPGHSPGIGH |  |
| CaCA\_scf\_133302\_222\_88 | 1 | 5.93e-14 |  | AFRPTTPGNSPGVGH |  |
| TaHA\_scf\_10\_1346\_150 | 1 | 6.44e-14 |  | DFKPTAPGHSPGVGH |  |
| PoTR\_chr\_07\_72858\_497 | 1 | 6.44e-14 |  | AFKPTTPGHSPGIGH |  |
| PoTR\_chr\_07\_72858\_416 | 1 | 6.44e-14 |  | AFKPTTPGHSPGIGH |  |
| PoTR\_chr\_07\_72858\_340 | 1 | 6.44e-14 |  | AFKPTTPGHSPGIGH |  |
| PoTR\_chr\_07\_72858\_264 | 1 | 6.44e-14 |  | AFKPTTPGHSPGIGH |  |
| PoTR\_chr\_07\_72674\_421 | 1 | 6.44e-14 |  | AFKPTTPGHSPGIGH |  |
| PoTR\_chr\_07\_72674\_340 | 1 | 6.44e-14 |  | AFKPTTPGHSPGIGH |  |
| PoTR\_chr\_07\_72674\_264 | 1 | 6.44e-14 |  | AFKPTTPGHSPGIGH |  |
| PoTR\_chr\_05\_109193\_222 | 1 | 6.44e-14 |  | AFKPTTPGHSPGIGH |  |
| PoEU\_scf\_623.1\_696\_181 | 1 | 6.44e-14 |  | AFKPTTPGHSPGIGH |  |
| PoEU\_scf\_354.1\_893\_181 | 1 | 6.44e-14 |  | AFKPTTPGHSPGIGH |  |
| PoEU\_scf\_234.1\_203\_353 | 1 | 6.44e-14 |  | AFKPTTPGHSPGIGH |  |
| GlMA\_chr\_11\_11219\_73 | 1 | 6.44e-14 |  | NFRPTAPGHSPGVGH |  |
| GlMA\_chr\_01\_174682\_117 | 1 | 6.44e-14 |  | NFRPTAPGHSPGVGH |  |
| CaSA.PK\_scf\_43257\_7\_97 | 1 | 6.44e-14 |  | NFRPTAPGHSPGVGH |  |
| CaSA.Fi\_ctg\_14469617\_5\_97 | 1 | 6.44e-14 |  | NFRPTAPGHSPGVGH |  |
| CaPA\_ctg\_1124\_137\_76 | 1 | 6.44e-14 |  | DLRPTTPGHSPGVGH |  |
| CaCA\_lg\_06\_45335\_70 | 1 | 6.44e-14 |  | NFRPTAPGHSPGVGH |  |
| BeNA\_scf\_29328\_15\_140 | 1 | 6.44e-14 |  | DLRPTTPGHSPGVGH |  |
| AzIN\_scf\_2708\_2\_71\_111 | 1 | 6.44e-14 |  | NFRPTAPGHSPGVGH |  |
| AzIN\_scf\_2708\_1\_30\_115 | 1 | 6.44e-14 |  | NFRPTAPGHSPGVGH |  |
| SiIR\_scf\_29\_6054\_87 | 1 | 7.08e-14 |  | AFRPTMPGHSPGVGH |  |
| LuAN\_scf\_82446\_1\_15\_74 | 1 | 7.08e-14 |  | DFRPTEPGHSPGAGH |  |
| ThCA.Cr\_chr\_08\_1541\_121 | 1 | 8.76e-14 |  | DFRPTSPGHSPGAGH |  |
| SeIN\_lg\_15\_9817\_70 | 1 | 8.76e-14 |  | DFRPTVPGHSPGIGH |  |
| PoTR\_chr\_05\_109193\_304 | 1 | 8.76e-14 |  | DFRPTVPGHSPGIGH |  |
| PoEU\_scf\_623.1\_696\_263 | 1 | 8.76e-14 |  | DFRPTVPGHSPGIGH |  |
| PoEU\_scf\_354.1\_893\_263 | 1 | 8.76e-14 |  | DFRPTVPGHSPGIGH |  |
| MaES\_chr\_xviii\_47386\_96 | 1 | 8.76e-14 |  | AFRPTYPGHSPGVGH |  |
| EuGR\_scf\_5\_238235\_141 | 1 | 8.76e-14 |  | AFRPTSPGHSLGVGH |  |
| EuCA\_ctg\_018397\_212\_119 | 1 | 8.76e-14 |  | AFRPTSPGHSLGVGH |  |
| RiCO\_ctg\_29676\_57\_280 | 1 | 9.44e-14 |  | DYRPTQPGHSPGVGH |  |
| PoTR\_chr\_07\_72858\_188 | 1 | 9.44e-14 |  | AFKPTTPGHSPGVGH |  |
| PoTR\_chr\_07\_72674\_188 | 1 | 9.44e-14 |  | AFKPTTPGHSPGVGH |  |
| PoEU\_scf\_234.1\_203\_196 | 1 | 9.44e-14 |  | AFKPTTPGHSPGVGH |  |
| MaES\_chr\_viii\_36047\_301 | 1 | 9.44e-14 |  | DYRPTQPGHSPGVGH |  |
| JaCU\_scf\_02121\_40\_338 | 1 | 9.44e-14 |  | DYRPTQPGHSPGVGH |  |
| HeBR\_scf\_155851\_157\_336 | 1 | 9.44e-14 |  | DYRPTQPGHSPGVGH |  |
| FrVE\_lg\_6\_109503\_147 | 1 | 9.44e-14 |  | DYRPTQPGHSPGVGH |  |
| FrNU\_scf\_00078196.1\_34\_147 | 1 | 9.44e-14 |  | DYRPTQPGHSPGVGH |  |
| FrNI\_ctg\_04327039.1\_4\_149 | 1 | 9.44e-14 |  | DYRPTQPGHSPGVGH |  |
| FrEX\_scf\_5019\_23\_91 | 1 | 1.01e-13 |  | DFRPTAPGKSPGIGH |  |
| EuGR\_scf\_9\_64398\_111 | 1 | 1.01e-13 |  | DFRSTEPGHSPGVGH |  |
| EuCA\_ctg\_045219\_187\_111 | 1 | 1.01e-13 |  | DFRSTEPGHSPGVGH |  |
| PoEU\_scf\_234.1\_363\_104 | 1 | 1.27e-13 |  | DFRPTVPGHSPGVGH |  |
| MaES\_chr\_viii\_75440\_84 | 1 | 1.27e-13 |  | DFRPTVPGHSPGVGH |  |
| HeBR\_scf\_420305\_30\_75 | 1 | 1.27e-13 |  | AFRPTQQGHSPGVGH |  |
| EuCA\_ctg\_010936\_1\_92 | 1 | 1.27e-13 |  | AFRLTSPGHSPGVGH |  |
| ViAN\_scf\_14509\_12\_162 | 1 | 1.66e-13 |  | DFKPTDPGHSPGVGH |  |
| ThCA.Ma\_chr\_8\_1795\_109 | 1 | 1.66e-13 |  | DFRPTTPGHSPGAGH |  |
| ThCA.Cr\_chr\_08\_1531\_109 | 1 | 1.66e-13 |  | DFRPTTPGHSPGAGH |  |
| PhVU\_chr\_03\_128036\_160 | 1 | 1.66e-13 |  | DFKPTDPGHSPGVGH |  |
| MiGU\_scf\_5\_94256\_71 | 1 | 1.66e-13 |  | AFRPTTPGNSPGMGH |  |
| MaES\_chr\_x\_67227\_73 | 1 | 1.66e-13 |  | AFRPTSPGHSPGAGH |  |
| LoJA\_chr\_5\_164713\_89 | 1 | 1.66e-13 |  | DFKPTDPGHSPGVGH |  |
| LiUS\_scf\_74\_1464\_96 | 1 | 1.66e-13 |  | DFRPTTPGHSPGAGH |  |
| LaSI\_ctg\_347625\_2\_102 | 1 | 1.66e-13 |  | DFRPTTPGHSPGAGH |  |
| GoRA\_chr\_10\_309123\_56 | 1 | 1.66e-13 |  | DFRPTTPGHSPGAGH |  |
| GlMA\_chr\_17\_184446\_168 | 1 | 1.66e-13 |  | DFKPTDPGHSPGVGH |  |
| GlMA\_chr\_05\_53925\_180 | 1 | 1.66e-13 |  | DFKPTDPGHSPGVGH |  |
| GlMA\_chr\_01\_174694\_145 | 1 | 1.66e-13 |  | DFKPTDPGHSPGVGH |  |
| EuGR\_scf\_5\_333720\_240 | 1 | 1.66e-13 |  | AFRPTSPGHSPGAGH |  |
| EuGR\_scf\_5\_240944\_159 | 1 | 1.66e-13 |  | AFRPTSPGHSPGAGH |  |
| EuGR\_scf\_5\_144942\_159 | 1 | 1.66e-13 |  | AFRPTSPGHSPGAGH |  |
| EuCA\_scf\_629621\_2\_218 | 1 | 1.66e-13 |  | AFRPTSPGHSPGAGH |  |
| EuCA\_scf\_629621\_2\_196 | 1 | 1.66e-13 |  | AFRPTSPGHSPGAGH |  |
| CuSA\_chr\_4\_70129\_127 | 1 | 1.66e-13 |  | DFRPTTPGHSPGAGH |  |
| CiSI\_scf\_00082\_585\_94 | 1 | 1.66e-13 |  | DFRPTTPGHSPGAGH |  |
| CiSI\_scf\_00002\_6043\_93 | 1 | 1.66e-13 |  | DFRPTTPGHSPGAGH |  |
| CiLA\_chr\_5\_104585\_93 | 1 | 1.66e-13 |  | DFRPTTPGHSPGAGH |  |
| CiCL\_scf\_8\_84116\_94 | 1 | 1.66e-13 |  | DFRPTTPGHSPGAGH |  |
| CiCL\_scf\_5\_107501\_93 | 1 | 1.66e-13 |  | DFRPTTPGHSPGAGH |  |
| CaCA\_scf\_133302\_184\_142 | 1 | 1.66e-13 |  | DFKPTDPGHSPGVGH |  |
| CaCA\_scf\_132880\_33\_64 | 1 | 1.66e-13 |  | AFRPTSPGHSPGAGH |  |
| AzIN\_scf\_2207\_1\_23\_218 | 1 | 1.66e-13 |  | DFRPTTPGHSPGAGH |  |
| RiCO\_ctg\_30170\_4822\_82 | 1 | 1.85e-13 |  | DFRPTNPGHSPGAGH |  |
| JaCU\_scf\_08269\_85\_70 | 1 | 1.85e-13 |  | DFRPTNPGHSPGAGH |  |
| JaCU\_scf\_04617\_69\_70 | 1 | 1.85e-13 |  | DFRPTNPGHSPGAGH |  |
| HeBR\_scf\_119199\_95\_60 | 1 | 1.85e-13 |  | DFRPTNPGHSPGAGH |  |
| ArTH\_chr\_2\_30548\_78 | 1 | 1.85e-13 |  | TFRPTEPGHSPGIGH |  |
| AmTR\_scf\_00270\_48\_270 | 1 | 1.85e-13 |  | DYRPTDPGHSPGVGH |  |
| AmTR\_scf\_00240\_313\_185 | 1 | 1.85e-13 |  | DYRPTDPGHSPGVGH |  |
| SeIN\_lg\_1\_108355\_72 | 1 | 2.00e-13 |  | AFRPTAPGNSPGMGH |  |
| PhVU\_chr\_02\_5513\_132 | 1 | 2.00e-13 |  | SFRPTEPGHSPGVGH |  |
| MoNO\_scf\_7113\_6\_143 | 1 | 2.00e-13 |  | DFRPTAPGHSPGAGH |  |
| MoNO\_scf\_467\_1582\_128 | 1 | 2.00e-13 |  | DFRPTAPGHSPGAGH |  |
| GoRA\_chr\_07\_252200\_102 | 1 | 2.00e-13 |  | DFRPTAPGHSPGAGH |  |
| ArTH\_chr\_2\_30559\_119 | 1 | 2.00e-13 |  | AFRSTEPGHSPGVGH |  |
| NeNU\_scf\_00045\_24649\_129 | 1 | 2.27e-13 |  | AFRPTTQGHSPGMGH |  |
| FrEX\_scf\_3403\_77\_99 | 1 | 2.27e-13 |  | AFRPTTPGHSPGMGN |  |
| FrEX\_scf\_2486\_721\_108 | 1 | 2.27e-13 |  | AFRPTTPGHSPGMGN |  |
| CiAR.Ka\_chr\_7\_183045\_93 | 1 | 2.27e-13 |  | AFRPTRPGHSPGVGH |  |
| CiAR.De\_lg\_7\_10832\_93 | 1 | 2.27e-13 |  | AFRPTRPGHSPGVGH |  |
| EuGR\_scf\_5\_333963\_356 | 1 | 2.61e-13 |  | AFQPTSPGHSPGVGH |  |
| EuGR\_scf\_5\_238372\_312 | 1 | 2.61e-13 |  | AFQPTSPGHSPGVGH |  |
| LiUS\_scf\_2416\_588\_318 | 1 | 2.89e-13 |  | DFRPTQPGHSPGAGH |  |
| LiUS\_scf\_2416\_588\_279 | 1 | 2.89e-13 |  | DFRPTQPGHSPGAGH |  |
| LiUS\_scf\_1249\_1842\_352 | 1 | 2.89e-13 |  | DFRPTQPGHSPGAGH |  |
| LiUS\_scf\_1249\_1842\_311 | 1 | 2.89e-13 |  | DFRPTQPGHSPGAGH |  |
| LiUS\_scf\_1249\_1842\_277 | 1 | 2.89e-13 |  | DFRPTQPGHSPGAGH |  |
| FrEX\_scf\_3405\_191\_116 | 1 | 2.89e-13 |  | DFRPTGPGHSPGIGH |  |
| CaRU\_scf\_8\_42165\_99 | 1 | 2.89e-13 |  | DFRPTRPGNSPGIGH |  |
| AzIN\_scf\_2207\_1\_23\_105 | 1 | 2.89e-13 |  | DYRPTTPGHSPGVGN |  |
| SeIN\_lg\_8\_120417\_68 | 1 | 3.19e-13 |  | SFRPTTPGHSPGIGH |  |
| NeNU\_scf\_00099\_6923\_157 | 1 | 3.19e-13 |  | SFRPTTPGHSPGIGH |  |
| MuBA\_chr\_3\_168239\_103 | 1 | 3.19e-13 |  | DVRPTTPGHSPGIGH |  |
| MuAC\_chr\_3\_185713\_108 | 1 | 3.19e-13 |  | DVRPTTPGHSPGIGH |  |
| MaES\_chr\_x\_67218\_142 | 1 | 3.19e-13 |  | AFRPTTPGHSPGAGH |  |
| FrEX\_scf\_8251\_14\_104 | 1 | 3.19e-13 |  | SFRPTTPGHSPGIGH |  |
| PhHE\_scf\_01003396\_771\_81 | 1 | 3.60e-13 |  | DVRPTNPGHSPGIGH |  |
| OrSA.Ja\_chr\_03\_64050\_85 | 1 | 3.60e-13 |  | DVRPTNPGHSPGIGH |  |
| OrSA.Ja\_chr\_03\_226607\_155 | 1 | 3.60e-13 |  | DVRPTNPGHSPGIGH |  |
| OrSA.Ja\_chr\_03\_226607\_129 | 1 | 3.60e-13 |  | DVRPTNPGHSPGIGH |  |
| OrSA.Ja\_chr\_03\_226607\_103 | 1 | 3.60e-13 |  | DVRPTNPGHSPGIGH |  |
| OrSA.In\_chr\_03\_67955\_85 | 1 | 3.60e-13 |  | DVRPTNPGHSPGIGH |  |
| OrSA.In\_chr\_03\_245043\_155 | 1 | 3.60e-13 |  | DVRPTNPGHSPGIGH |  |
| OrSA.In\_chr\_03\_245043\_129 | 1 | 3.60e-13 |  | DVRPTNPGHSPGIGH |  |
| OrSA.In\_chr\_03\_245043\_103 | 1 | 3.60e-13 |  | DVRPTNPGHSPGIGH |  |
| OrBR\_chr\_3\_48638\_82 | 1 | 3.60e-13 |  | DVRPTNPGHSPGIGH |  |
| EuGR\_scf\_5\_333937\_268 | 1 | 3.60e-13 |  | AFRPTSPAHSPGVGH |  |
| EuCA\_ctg\_023070\_58\_224 | 1 | 3.60e-13 |  | TFRPTSPGHSPGVGH |  |
| ArDU\_chr\_05\_21732\_121 | 1 | 3.60e-13 |  | TFRPTSPGHSPGVGH |  |
| TaHA\_scf\_3\_40844\_115 | 1 | 3.94e-13 |  | SFRPTAPGHSPGIGH |  |
| NeNU\_scf\_00099\_6847\_125 | 1 | 4.37e-13 |  | GFRPTTPGHSPGIGH |  |
| MaES\_chr\_viii\_36047\_75 | 1 | 4.37e-13 |  | DFRPTSPGFSPGVGH |  |
| MaES\_chr\_viii\_36047\_267 | 1 | 4.37e-13 |  | DFRPTSPGFSPGVGH |  |
| LiUS\_scf\_2416\_569\_84 | 1 | 4.37e-13 |  | GFRPTTPGHSPGIGH |  |
| HeBR\_scf\_155851\_157\_302 | 1 | 4.37e-13 |  | DFRPTSPGFSPGVGH |  |
| GoRA\_chr\_03\_227090\_103 | 1 | 4.37e-13 |  | DFRPTGPGHSPGVGH |  |
| GnGN\_ctg\_139619\_2\_75 | 1 | 4.37e-13 |  | ALRPTSSGHSPGVGH |  |
| EuGR\_scf\_9\_63945\_151 | 1 | 4.37e-13 |  | DFRPTGPGHSPGVGH |  |
| EuGR\_scf\_8\_418948\_218 | 1 | 4.37e-13 |  | DFRPTGPGHSPGVGH |  |
| EuCA\_ctg\_051094\_155\_177 | 1 | 4.37e-13 |  | DFRPTGPGHSPGVGH |  |
| AmTR\_scf\_00240\_74\_117 | 1 | 4.37e-13 |  | DFRPTDPGLSPGVGH |  |
| PyCO\_scf\_00016\_3652\_73 | 1 | 4.82e-13 |  | SFRPTTPGHSPGVGH |  |
| PyBR\_scf\_4.0\_9812\_73 | 1 | 4.82e-13 |  | SFRPTTPGHSPGVGH |  |
| MiGU\_scf\_8\_8701\_110 | 1 | 4.82e-13 |  | AFQPTTPGHSPGVGH |  |
| MaDO\_chr\_2\_149047\_73 | 1 | 4.82e-13 |  | SFRPTTPGHSPGVGH |  |
| LuAN\_scf\_14424\_14\_118 | 1 | 4.82e-13 |  | AFRPTSPGDSPGIGH |  |
| GoRA\_chr\_08\_258111\_85 | 1 | 4.82e-13 |  | DFRRTSPGHSPGIGH |  |
| FrVE\_lg\_2\_88445\_87 | 1 | 4.82e-13 |  | DYRPTEPGHSPGAGH |  |
| FrNU\_scf\_00085996.1\_9\_87 | 1 | 4.82e-13 |  | DYRPTEPGHSPGAGH |  |
| FrNI\_ctg\_04412679.1\_6\_87 | 1 | 4.82e-13 |  | DYRPTEPGHSPGAGH |  |
| FrII\_scf\_00054285.1\_8\_87 | 1 | 4.82e-13 |  | DYRPTEPGHSPGAGH |  |
| EnVE\_ctg\_22030\_4646726\_1\_11\_105 | 1 | 4.82e-13 |  | DVRPTTPGHSPGVGH |  |
| PhVU\_chr\_01\_290172\_90 | 1 | 5.41e-13 |  | DFRPTDPGHSPGAGH |  |
| MuBA\_chr\_un\_random\_228501\_97 | 1 | 5.41e-13 |  | DVRPTNPGHSPGVGH |  |
| MuBA\_chr\_1\_49638\_81 | 1 | 5.41e-13 |  | DVRPTNPGHSPGVGH |  |
| MuAC\_chr\_un\_random\_176676\_97 | 1 | 5.41e-13 |  | DVRPTNPGHSPGVGH |  |
| MuAC\_chr\_1\_53460\_83 | 1 | 5.41e-13 |  | DVRPTNPGHSPGVGH |  |
| MeTR\_chr\_1\_15289\_85 | 1 | 5.41e-13 |  | DFRPTDPGHSPGAGH |  |
| LeAL\_scf\_12\_1242\_72 | 1 | 5.41e-13 |  | TFRPTAPGHSPGIGH |  |
| HeBR\_scf\_413830\_5\_75 | 1 | 5.41e-13 |  | DFRPTDPGHSPGAGH |  |
| GlMA\_chr\_17\_113157\_91 | 1 | 5.41e-13 |  | DFRPTDPGHSPGAGH |  |
| GlMA\_chr\_03\_19720\_68 | 1 | 5.41e-13 |  | DFRPTDPGHSPGAGH |  |
| EuGR\_scf\_5\_333963\_114 | 1 | 5.41e-13 |  | AFRSTNPGHSPGVGH |  |
| EuGR\_scf\_5\_238235\_163 | 1 | 5.41e-13 |  | AFRSTNPGHSPGVGH |  |
| CaRU\_scf\_4\_3227\_98 | 1 | 5.41e-13 |  | NFRPTVPGHSPGIGH |  |
| CaCA\_scf\_132748\_1420\_95 | 1 | 5.41e-13 |  | DFRPTDPGHSPGAGH |  |
| BrRA\_chr\_02\_76978\_71 | 1 | 5.41e-13 |  | AFRPTRPGNSPGIGH |  |
| BeNA\_scf\_7080\_88\_107 | 1 | 5.41e-13 |  | DFRPTDPGHSPGAGH |  |
| ArLY\_scf\_4\_5687\_63 | 1 | 5.41e-13 |  | TFRPTAPGHSPGIGH |  |
| ArIP\_chr\_07\_426831\_88 | 1 | 5.41e-13 |  | DFRPTDPGHSPGAGH |  |
| ArIP\_chr\_06\_412349\_67 | 1 | 5.41e-13 |  | DFRPTDPGHSPGAGH |  |
| ArDU\_chr\_08\_250174\_88 | 1 | 5.41e-13 |  | DFRPTDPGHSPGAGH |  |
| ArDU\_chr\_06\_314502\_129 | 1 | 5.41e-13 |  | DFRPTDPGHSPGAGH |  |
| AqCO\_scf\_15\_2745\_75 | 1 | 5.41e-13 |  | DFRPTAPGHSPSVGH |  |
| SpOL\_scf\_25714\_168\_412 | 1 | 5.95e-13 |  | DYRPTKPGHSPGAGH |  |
| PyCO\_scf\_00043\_728\_76 | 1 | 5.95e-13 |  | DYRPTKPGHSPGAGH |  |
| FrVE\_lg\_6\_109503\_107 | 1 | 5.95e-13 |  | DFRPTTPGNSPGAGH |  |
| FrNU\_scf\_00078196.1\_34\_107 | 1 | 5.95e-13 |  | DFRPTTPGNSPGAGH |  |
| FrNI\_ctg\_04327039.1\_4\_109 | 1 | 5.95e-13 |  | DFRPTTPGNSPGAGH |  |
| LiUS\_scf\_1249\_1817\_84 | 1 | 6.50e-13 |  | GFRPTTPGHSPGVGH |  |
| CiAR.Ka\_chr\_8\_10022\_61 | 1 | 6.50e-13 |  | TFRPTTPGHSPGVGH |  |
| CiAR.De\_lg\_3\_37933\_61 | 1 | 6.50e-13 |  | TFRPTTPGHSPGVGH |  |
| LuAN\_scf\_76000\_49\_104 | 1 | 7.12e-13 |  | AFRPTSPGDSPGVGH |  |
| HeBR\_scf\_362425\_115\_100 | 1 | 7.12e-13 |  | GFRPTNPGHSPGVGH |  |
| PyBR\_scf\_520.0\_2943\_100 | 1 | 7.92e-13 |  | DFRPTTAGNSPGVGH |  |
| EuGR\_scf\_6\_70588\_99 | 1 | 7.92e-13 |  | NFRSTKPGHSPGVGH |  |
| EuGR\_scf\_6\_122736\_90 | 1 | 7.92e-13 |  | NFRSTKPGHSPGVGH |  |
| EuCA\_scf\_751148\_11\_90 | 1 | 7.92e-13 |  | NFRSTKPGHSPGVGH |  |
| EuCA\_ctg\_076399\_2\_90 | 1 | 7.92e-13 |  | NFRSTKPGHSPGVGH |  |
| EuCA\_ctg\_011619\_37\_99 | 1 | 7.92e-13 |  | NFRSTKPGHSPGVGH |  |
| ArTH\_chr\_5\_82449\_88 | 1 | 7.92e-13 |  | DFEPTTPGHSPGVGH |  |
| ArLY\_scf\_8\_63500\_90 | 1 | 7.92e-13 |  | DFEPTTPGHSPGVGH |  |
| PoTR\_chr\_07\_72858\_146 | 1 | 8.62e-13 |  | DFQPTTPGNSPGVGH |  |
| PoTR\_chr\_07\_72674\_146 | 1 | 8.62e-13 |  | DFQPTTPGNSPGVGH |  |
| SiIR\_scf\_24\_20762\_72 | 1 | 9.41e-13 |  | DFRSTNPGNSPGVGH |  |
| RiCO\_ctg\_29676\_57\_246 | 1 | 9.41e-13 |  | DFRPTAPGFSPGVGH |  |
| PoTR\_chr\_14\_7933\_75 | 1 | 9.41e-13 |  | DFKPTTPGHSPGAGH |  |
| PoEU\_scf\_2.1\_3612\_75 | 1 | 9.41e-13 |  | DFKPTTPGHSPGAGH |  |
| NeNU\_scf\_00003\_56239\_98 | 1 | 9.41e-13 |  | DIRPTAPGHSPGVGH |  |
| LiUS\_scf\_2416\_588\_203 | 1 | 9.41e-13 |  | DFRPTAPGFSPGVGH |  |
| LiUS\_scf\_1249\_1842\_201 | 1 | 9.41e-13 |  | DFRPTAPGFSPGVGH |  |
| EuGR\_scf\_5\_238372\_136 | 1 | 9.41e-13 |  | AFRPTSPRHSPGVGH |  |
| EuCA\_ctg\_023562\_269\_122 | 1 | 9.41e-13 |  | DFRPTEPGYSPGVGR |  |
| BrRA\_chr\_08\_110158\_109 | 1 | 9.41e-13 |  | DFRSTNPGNSPGVGH |  |
| BeVU\_chr\_6.sca003\_7759\_301 | 1 | 9.41e-13 |  | EYRPTKPGHSPGAGH |  |
| AqCO\_scf\_15\_2747\_95 | 1 | 9.41e-13 |  | DIRPTAPGHSPGVGH |  |
| AmTR\_scf\_00157\_136\_112 | 1 | 9.41e-13 |  | DLRSTSPGHSPGVGH |  |
| PyBR\_scf\_154.0\_468\_76 | 1 | 1.04e-12 |  | DYRPTTPGHSPGAGH |  |
| PiTA\_scf\_t8959\_80\_129 | 1 | 1.04e-12 |  | AFRPSSSGHSPGIGH |  |
| PiTA\_scf\_t3921\_878\_153 | 1 | 1.04e-12 |  | AFRPSSSGHSPGIGH |  |
| PiTA\_scf\_t3921\_3572\_129 | 1 | 1.04e-12 |  | AFRPSSSGHSPGIGH |  |
| PiTA\_scf\_901296\_275\_360 | 1 | 1.04e-12 |  | AFRPSSSGHSPGIGH |  |
| PiTA\_scf\_881528\_51\_149 | 1 | 1.04e-12 |  | AFRPSSSGHSPGIGH |  |
| PiTA\_scf\_789033\_82\_129 | 1 | 1.04e-12 |  | AFRPSSSGHSPGIGH |  |
| PiTA\_scf\_274065\_340\_153 | 1 | 1.04e-12 |  | AFRPSSSGHSPGIGH |  |
| PiTA\_scf\_185885\_469\_129 | 1 | 1.04e-12 |  | AFRPSSSGHSPGIGH |  |
| PiTA\_ctg\_30790360\_8\_155 | 1 | 1.04e-12 |  | AFRPSSSGHSPGIGH |  |
| PiTA\_ctg\_28724920\_2\_129 | 1 | 1.04e-12 |  | AFRPSSSGHSPGIGH |  |
| PiSY\_ctg\_6288925\_3\_164 | 1 | 1.04e-12 |  | AFRPTSSGHSPGAGH |  |
| PiSY\_ctg\_10265915\_4\_149 | 1 | 1.04e-12 |  | AFRPSSSGHSPGIGH |  |
| PiGL\_ctg\_168152959\_3\_5\_153 | 1 | 1.04e-12 |  | AFRPSSSGHSPGIGH |  |
| PiGL\_ctg\_168009378\_8\_28\_159 | 1 | 1.04e-12 |  | AFRPSSSGHSPGIGH |  |
| PiGL\_ctg\_167936109\_6\_41\_156 | 1 | 1.04e-12 |  | AFRPSSSGHSPGIGH |  |
| PiGL\_ctg\_167670474\_0\_12\_135 | 1 | 1.04e-12 |  | AFRPSSSGHSPGIGH |  |
| PiGL\_ctg\_164078179\_0\_4\_137 | 1 | 1.04e-12 |  | AFRPSSSGHSPGIGH |  |
| PiAB\_ctg\_9991140\_55\_135 | 1 | 1.04e-12 |  | AFRPSSSGHSPGIGH |  |
| PiAB\_ctg\_490634\_20\_149 | 1 | 1.04e-12 |  | AFRPSSSGHSPGIGH |  |
| PiAB\_ctg\_433845\_9\_153 | 1 | 1.04e-12 |  | AFRPSSSGHSPGIGH |  |
| PiAB\_ctg\_3289\_442\_136 | 1 | 1.04e-12 |  | AFRPSSSGHSPGIGH |  |
| MaDO\_chr\_15\_8414\_76 | 1 | 1.04e-12 |  | DYRPTTPGHSPGAGH |  |
| LiUS\_scf\_650\_806\_167 | 1 | 1.04e-12 |  | GFRPTQPGHSPGVGH |  |
| FrEX\_scf\_2186\_496\_114 | 1 | 1.04e-12 |  | GFRPTQPGHSPGVGH |  |
| EuGR\_scf\_8\_418753\_122 | 1 | 1.04e-12 |  | DFRPTEPGNSPGVGR |  |
| EuGR\_scf\_5\_333963\_334 | 1 | 1.04e-12 |  | AFRPISPGHSPGVGH |  |
| CuME\_chr\_07\_6955\_127 | 1 | 1.04e-12 |  | DFRPTTSGHSPGAGH |  |
| CiAR.Ka\_chr\_7\_27979\_119 | 1 | 1.04e-12 |  | DFKKTDPGHSPGVGH |  |
| PhHE\_scf\_01002760\_234\_79 | 1 | 1.14e-12 |  | DVRPTNPGNSPGIGH |  |
| MaDO\_chr\_15\_179591\_110 | 1 | 1.14e-12 |  | DFRPTTPGNSPAVGH |  |
| FrEX\_scf\_2682\_616\_230 | 1 | 1.14e-12 |  | DFRPTTPGHSPGFGH |  |
| EuGR\_scf\_5\_238372\_158 | 1 | 1.14e-12 |  | AFRPTSPSHSPGVGH |  |
| EuGR\_scf\_5\_238372\_114 | 1 | 1.14e-12 |  | AFRPTSPSHSPGVGH |  |
| EuGR\_scf\_5\_238235\_97 | 1 | 1.14e-12 |  | AFRQTSPGHSPKVGH |  |
| EuGR\_scf\_5\_238235\_119 | 1 | 1.14e-12 |  | AFRPTSPSHSPGVGH |  |
| EuCA\_ctg\_018397\_212\_97 | 1 | 1.14e-12 |  | AFRQTSPGHSPKVGH |  |
| PoTR\_chr\_05\_109193\_108 | 1 | 1.24e-12 |  | DFRPTTPGVSPGVGH |  |
| ScPA\_chr\_2-4\_32083\_81 | 1 | 1.37e-12 |  | GFRPTTPGNSPGIGH |  |
| PhDA\_scf\_001357\_177\_113 | 1 | 1.37e-12 |  | DARPTTPGHSPGVGH |  |
| PhDA\_scf\_000109\_280\_135 | 1 | 1.37e-12 |  | DARPTTPGHSPGVGH |  |
| PhDA\_scf\_000017\_10633\_135 | 1 | 1.37e-12 |  | DARPTTPGHSPGVGH |  |
| FrEX\_scf\_1891\_617\_90 | 1 | 1.37e-12 |  | DLRSTAPGHSPGIGH |  |
| PiTA\_scf\_888115\_424\_135 | 1 | 1.51e-12 |  | AFRPSSSGHSPGVGH |  |
| MeTR\_ctg\_59554\_7\_127 | 1 | 1.51e-12 |  | AFQPTTPGNSPGVGH |  |
| LuAN\_scf\_13705\_12\_160 | 1 | 1.51e-12 |  | GFKPTEPGHSPGVGH |  |
| LiUS\_scf\_2416\_588\_124 | 1 | 1.51e-12 |  | DFRPTAPGVSPGVGH |  |
| LiUS\_scf\_1249\_1842\_121 | 1 | 1.51e-12 |  | DFRPTAPGVSPGVGH |  |
| PyBR\_scf\_145.0\_80\_113 | 1 | 1.63e-12 |  | DFRPTAPGYSPGVAH |  |
| PoTR\_chr\_05\_109193\_182 | 1 | 1.63e-12 |  | AFKPTTPGHSPGAGH |  |
| EuGR\_scf\_5\_238372\_92 | 1 | 1.63e-12 |  | AFRPTSPGHSPRVGH |  |
| SeIN\_lg\_15\_9806\_116 | 1 | 1.77e-12 |  | SYRPTTPGHSPGIGH |  |
| MiGU\_scf\_8\_8710\_67 | 1 | 1.77e-12 |  | SYRPTTPGHSPGIGH |  |
| MaES\_chr\_xviii\_47380\_625 | 1 | 1.77e-12 |  | GFRPTTPGYSPGVGH |  |
| CiAR.Ka\_chr\_7\_27979\_74 | 1 | 1.77e-12 |  | AFQPTAPGNSPGVGH |  |
| ArLY\_scf\_8\_33647\_340 | 1 | 1.77e-12 |  | DFEPTNPGNSPGIGH |  |
| SiIR\_scf\_20\_535\_160 | 1 | 1.94e-12 |  | DFAPTSPGHSPGVGH |  |
| CaCA\_lg\_10\_34380\_98 | 1 | 1.94e-12 |  | GFRPTTPGNSPGVGH |  |
| ArTH\_chr\_3\_89083\_129 | 1 | 1.94e-12 |  | DFAPTSPGHSPGVGH |  |
| ArLY\_scf\_5\_79011\_128 | 1 | 1.94e-12 |  | DFAPTSPGHSPGVGH |  |
| PyCO\_scf\_00016\_3634\_99 | 1 | 2.11e-12 |  | DFRPTTPGSSPGVGH |  |
| PyBR\_scf\_4.0\_9791\_99 | 1 | 2.11e-12 |  | DFRPTTPGSSPGVGH |  |
| MaDO\_chr\_2\_149034\_94 | 1 | 2.11e-12 |  | DFRPTTPGSSPGVGH |  |
| GoRA\_chr\_03\_227050\_71 | 1 | 2.11e-12 |  | DFRPTTPGSSPGVGH |  |
| FrEX\_scf\_3405\_191\_87 | 1 | 2.11e-12 |  | SFRPTSPGKSPGIGH |  |
| EuGR\_scf\_8\_418948\_174 | 1 | 2.11e-12 |  | DFRPTTPGSSPGVGH |  |
| EuGR\_scf\_8\_418858\_131 | 1 | 2.11e-12 |  | DFRPTTPGSSPGVGH |  |
| EuGR\_scf\_8\_418581\_121 | 1 | 2.11e-12 |  | DFRPTTPGSSPGVGH |  |
| EuCA\_ctg\_051094\_155\_133 | 1 | 2.11e-12 |  | DFRPTTPGSSPGVGH |  |
| EuCA\_ctg\_034442\_93\_105 | 1 | 2.11e-12 |  | DFRPTTPGSSPGVGH |  |
| RiCO\_ctg\_29827\_2918\_83 | 1 | 2.48e-12 |  | AFRPTAPGHSSGVGH |  |
| PyCO\_scf\_00781\_604\_101 | 1 | 2.48e-12 |  | NFRPTTPGYSPGAGH |  |
| PyBR\_scf\_190.0\_3108\_129 | 1 | 2.48e-12 |  | NFRPTTPGYSPGAGH |  |
| PoEU\_scf\_234.1\_203\_272 | 1 | 2.48e-12 |  | AFKPTTPGHSPSIGH |  |
| MeTR\_ctg\_59554\_7\_174 | 1 | 2.48e-12 |  | EFQKTNPGHSPGVGH |  |
| EuGR\_scf\_9\_63505\_188 | 1 | 2.48e-12 |  | DFQSTEPGHSPGVGH |  |
| EuGR\_scf\_6\_193279\_158 | 1 | 2.48e-12 |  | DFQSTEPGHSPGVGH |  |
| EuGR\_scf\_1325\_3\_158 | 1 | 2.48e-12 |  | DFQSTEPGHSPGVGH |  |
| EuCA\_ctg\_049989\_43\_158 | 1 | 2.48e-12 |  | DFQSTEPGHSPGVGH |  |
| BeVU\_chr\_6.sca003\_7759\_142 | 1 | 2.48e-12 |  | DFRPTAPGKSPGAGH |  |
| AzIN\_scf\_2207\_1\_23\_156 | 1 | 2.48e-12 |  | GFRPTTPGHSPGVGN |  |
| ArTH\_chr\_1\_42237\_136 | 1 | 2.48e-12 |  | DFAPTNPGHSPGIGH |  |
| ArLY\_scf\_8\_33647\_415 | 1 | 2.48e-12 |  | DFAPTNPGHSPGIGH |  |
| ArLY\_scf\_8\_33647\_365 | 1 | 2.48e-12 |  | DFAPTNPGHSPGIGH |  |
| ArLY\_scf\_8\_33647\_315 | 1 | 2.48e-12 |  | DFAPTNPGHSPGIGH |  |
| ArLY\_scf\_8\_33647\_265 | 1 | 2.48e-12 |  | DFAPTNPGHSPGIGH |  |
| ArLY\_scf\_8\_33647\_215 | 1 | 2.48e-12 |  | DFAPTNPGHSPGIGH |  |
| ArLY\_scf\_8\_33647\_140 | 1 | 2.48e-12 |  | DFAPTNPGHSPGIGH |  |
| ArLY\_scf\_2\_10720\_415 | 1 | 2.48e-12 |  | DFAPTNPGHSPGIGH |  |
| ArLY\_scf\_2\_10720\_365 | 1 | 2.48e-12 |  | DFAPTNPGHSPGIGH |  |
| ArLY\_scf\_2\_10720\_315 | 1 | 2.48e-12 |  | DFAPTNPGHSPGIGH |  |
| ArLY\_scf\_2\_10720\_215 | 1 | 2.48e-12 |  | DFAPTNPGHSPGIGH |  |
| ArLY\_scf\_2\_10720\_165 | 1 | 2.48e-12 |  | DFAPTNPGHSPGIGH |  |
| ArLY\_scf\_2\_10720\_140 | 1 | 2.48e-12 |  | DFAPTNPGHSPGIGH |  |
| SiIR\_scf\_8\_11146\_88 | 1 | 2.71e-12 |  | DFKPTTPGNSPGVEH |  |
| PiTA\_scf\_880179.2\_309\_110 | 1 | 2.71e-12 |  | AFRPSSSGHSPGIGY |  |
| ElOL\_scf\_00187\_4136\_91 | 1 | 2.71e-12 |  | DTRPTTPGHSPGVGH |  |
| ElGU\_chr\_7\_48393\_96 | 1 | 2.71e-12 |  | DTRPTTPGHSPGVGH |  |
| ElGU\_chr\_3\_71946\_129 | 1 | 2.71e-12 |  | DTRPTTPGHSPGVGH |  |
| SpPO\_chr\_4\_56998\_137 | 1 | 2.93e-12 |  | GLRPTAPGHSPGIGH |  |
| GoRA\_chr\_07\_301853\_82 | 1 | 2.93e-12 |  | DFWPTAPGNSPGIGH |  |
| AmTR\_scf\_00676\_17\_90 | 1 | 2.93e-12 |  | DFRPTDPSHSPGVGH |  |
| ViAN\_scf\_14509\_28\_150 | 1 | 3.18e-12 |  | GFKPTNPGHSPGVGH |  |
| SeIN\_lg\_3\_62711\_91 | 1 | 3.18e-12 |  | DFAPTTPGHSPGVGH |  |
| PhVU\_chr\_03\_128057\_149 | 1 | 3.18e-12 |  | GFKPTNPGHSPGVGH |  |
| GlMA\_chr\_17\_184876\_169 | 1 | 3.18e-12 |  | GFKPTNPGHSPGVGH |  |
| GlMA\_chr\_17\_184560\_164 | 1 | 3.18e-12 |  | GFKPTNPGHSPGVGH |  |
| CaCA\_scf\_133302\_222\_135 | 1 | 3.18e-12 |  | GFKPTNPGHSPGVGH |  |
| ViAN\_scf\_14509\_28\_104 | 1 | 3.48e-12 |  | AFRPTTPGSSPGVGH |  |
| SpOL\_scf\_24951\_21\_107 | 1 | 3.48e-12 |  | AFRPTTPGLSPGAGH |  |
| PoTR\_chr\_05\_109193\_142 | 1 | 3.48e-12 |  | DFQPTTPGHSPGAGH |  |
| PiGL\_ctg\_167815528\_5\_4\_183 | 1 | 3.48e-12 |  | AFRPSSSGHSPGMGH |  |
| PiGL\_ctg\_163248640\_0\_5\_163 | 1 | 3.48e-12 |  | AFRPSSSGHSPGMGH |  |
| PiGL\_ctg\_162771921\_0\_12\_163 | 1 | 3.48e-12 |  | AFRPSSSGHSPGMGH |  |
| PiGL\_ctg\_161449300\_0\_17\_163 | 1 | 3.48e-12 |  | AFRPSSSGHSPGMGH |  |
| PiAB\_ctg\_18434\_210\_178 | 1 | 3.48e-12 |  | AFRPSSSGHSPGMGH |  |
| MeTR\_scf\_ac233112.6\_181\_104 | 1 | 3.48e-12 |  | AFRPTTPGSSPGVGH |  |
| LuAN\_scf\_68177\_1\_18\_75 | 1 | 3.48e-12 |  | AFRPTTPGSSPGVGH |  |
| GoRA\_chr\_05\_44934\_87 | 1 | 3.48e-12 |  | DFQPTTPGHSPGAGH |  |
| CaCA\_lg\_06\_45369\_90 | 1 | 3.48e-12 |  | AFRPTTPGSSPGVGH |  |
| PiGL\_ctg\_168012670\_1\_14\_153 | 1 | 3.80e-12 |  | AFRPASSGHSPGMGH |  |
| PiGL\_ctg\_167910995\_40\_6\_153 | 1 | 3.80e-12 |  | AFRPASSGHSPGMGH |  |
| PiAB\_ctg\_18434\_18\_153 | 1 | 3.80e-12 |  | AFRPASSGHSPGMGH |  |
| PhDA\_scf\_000134\_4732\_559 | 1 | 3.80e-12 |  | DSRPTAPGHSPGVGH |  |
| CaSA.Fi\_ctg\_14326822\_1\_100 | 1 | 3.80e-12 |  | GFRPTEPGHSPGAGH |  |
| CaRU\_scf\_8\_42174\_72 | 1 | 3.80e-12 |  | DFAPTSPGNSPGIGH |  |
| CaRU\_scf\_5\_58226\_158 | 1 | 3.80e-12 |  | DFAPTSPGNSPGIGH |  |
| BrRA\_chr\_09\_146618\_104 | 1 | 3.80e-12 |  | DFAPTSPGNSPGIGH |  |
| ArTH\_chr\_3\_89083\_181 | 1 | 3.80e-12 |  | DFAPTSPGNSPGIGH |  |
| SpOL\_scf\_25714\_168\_240 | 1 | 4.09e-12 |  | DFRSTAPGHSPGAGH |  |
| HeBR\_scf\_155851\_157\_203 | 1 | 4.09e-12 |  | DFRPTDPGNSPYVGH |  |
| AmTR\_scf\_00157\_397\_87 | 1 | 4.09e-12 |  | DLKSTSPGHSPGVGH |  |
| SiIR\_scf\_29\_6036\_65 | 1 | 4.48e-12 |  | TFRPTVPGHSPGIGH |  |
| ScPA\_chr\_4-2\_312\_148 | 1 | 4.48e-12 |  | TFRPTVPGHSPGIGH |  |
| PrPE\_scf\_1\_122064\_74 | 1 | 4.48e-12 |  | NYRPTTPGHSPGAGH |  |
| PrMU\_chr\_2\_241459\_151 | 1 | 4.48e-12 |  | NYRPTTPGHSPGAGH |  |
| LuAN\_scf\_76000\_49\_148 | 1 | 4.48e-12 |  | GFKPTQPGHSPGVGH |  |
| EuSA\_scf\_15\_6258\_73 | 1 | 4.48e-12 |  | TFRPTVPGHSPGIGH |  |
| EuCA\_ctg\_023070\_58\_180 | 1 | 4.48e-12 |  | AFKPISPGHSPGVGH |  |
| BrRA\_chr\_09\_146618\_156 | 1 | 4.48e-12 |  | DFAPTSPGHSPGMGH |  |
| BrRA\_chr\_09\_128063\_65 | 1 | 4.48e-12 |  | TFRPTVPGHSPGIGH |  |
| SpOL\_scf\_25714\_168\_372 | 1 | 4.87e-12 |  | DFRPTKPGDSPGAGH |  |
| MaES\_chr\_viii\_36047\_169 | 1 | 4.87e-12 |  | DFRPTPPGHSPGLGH |  |
| FrEX\_scf\_2186\_496\_76 | 1 | 4.87e-12 |  | GFRPTNPGKSPGIGH |  |
| EuGR\_scf\_5\_333937\_158 | 1 | 4.87e-12 |  | AFRPTSHGHSPGVGH |  |
| ElGU\_chr\_15\_38527\_418 | 1 | 4.87e-12 |  | DGRPTEPGHSPGVGH |  |
| SiIR\_scf\_20\_535\_108 | 1 | 5.29e-12 |  | DFAPTSPGNSPGVGH |  |
| ScPA\_chr\_5-1\_14490\_98 | 1 | 5.29e-12 |  | DFAPTSPGNSPGVGH |  |
| ScPA\_chr\_5-1\_14490\_202 | 1 | 5.29e-12 |  | DFAPTSPGNSPGVGH |  |
| HeBR\_scf\_243691\_91\_313 | 1 | 5.29e-12 |  | DYRPTHPGHSPGVGH |  |
| FrEX\_scf\_5019\_23\_120 | 1 | 5.29e-12 |  | DFRSTGPGHSPGIGH |  |
| EuGR\_scf\_8\_418792\_134 | 1 | 5.29e-12 |  | DFRPTTPGTSPGVGN |  |
| CaCA\_scf\_127737\_1130\_86 | 1 | 5.29e-12 |  | DFRPTDPGHSPGVRH |  |
| ArLY\_scf\_5\_79011\_76 | 1 | 5.29e-12 |  | DFAPTSPGNSPGVGH |  |
| SeIN\_lg\_15\_9821\_107 | 1 | 5.76e-12 |  | DSKPTSPGHSPGIGH |  |
| NiTO\_scf\_28409\_188\_97 | 1 | 5.76e-12 |  | AFQPSTPGHSPGIGH |  |
| KaFE\_scf\_1360502\_67\_85 | 1 | 5.76e-12 |  | SFRPTTPGHSPGAGH |  |
| GoRA\_chr\_09\_302341\_103 | 1 | 5.76e-12 |  | AFRSTTPGHSPGAGH |  |
| EuGR\_scf\_5\_333720\_218 | 1 | 5.76e-12 |  | AFRPTSPGHSPRVGN |  |
| ArLY\_scf\_8\_33647\_165 | 1 | 5.76e-12 |  | DFVPTNPGHSPGIGH |  |
| ArLY\_scf\_5\_79011\_168 | 1 | 5.76e-12 |  | EFAPTSPGNSPGIGH |  |
| AmTR\_scf\_00270\_119\_63 | 1 | 5.76e-12 |  | NFRPTDPDHSPGVGH |  |
| SpOL\_scf\_33414\_69\_78 | 1 | 6.29e-12 |  | DFRPTTPGRSPGAGH |  |
| SpOL\_scf\_24951\_60\_82 | 1 | 6.29e-12 |  | AFRPTSPGRSPGAGH |  |
| SoBI\_chr\_01\_202134\_105 | 1 | 6.29e-12 |  | DVRPSNPGHSPGIGH |  |
| LuAN\_scf\_76092\_44\_98 | 1 | 6.29e-12 |  | GFRPTVPGHSPGVGH |  |
| BrDI\_chr\_1\_402044\_63 | 1 | 6.29e-12 |  | DVRPSNPGHSPGIGH |  |
| BeVU\_chr\_6.sca003\_7843\_117 | 1 | 6.29e-12 |  | DFRPTTPGRSPGAGH |  |
| RiCO\_ctg\_29676\_57\_89 | 1 | 6.83e-12 |  | AFRPTPPGNSPGVGH |  |
| NeNU\_scf\_00099\_6989\_89 | 1 | 6.83e-12 |  | SFRPTAPGHSPGAGH |  |
| KaFE\_scf\_1368498\_122\_83 | 1 | 6.83e-12 |  | NFRPTALGHSPGAGH |  |
| ArLY\_scf\_8\_33647\_290 | 1 | 6.83e-12 |  | DFAPTNPGNSPGIGH |  |
| ArLY\_scf\_8\_33647\_240 | 1 | 6.83e-12 |  | DFAPTNPGNSPGIGH |  |
| ArLY\_scf\_2\_10720\_390 | 1 | 6.83e-12 |  | DFAPTNPGNSPGIGH |  |
| ArLY\_scf\_2\_10720\_290 | 1 | 6.83e-12 |  | DFAPTNPGNSPGIGH |  |
| ArLY\_scf\_2\_10720\_240 | 1 | 6.83e-12 |  | DFAPTNPGNSPGIGH |  |
| ArLY\_scf\_2\_10720\_190 | 1 | 6.83e-12 |  | DFAPTNPGNSPGIGH |  |
| AeAR\_scf\_13\_713\_103 | 1 | 6.83e-12 |  | DFAPTNPGNSPGIGH |  |
| PhHE\_scf\_01007655\_65\_77 | 1 | 7.43e-12 |  | DSRPTAPGNSPGIGH |  |
| LiUS\_scf\_464\_9241\_88 | 1 | 7.43e-12 |  | AFRPTTPGHSPGVCC |  |
| KaFE\_scf\_1368498\_168\_107 | 1 | 7.43e-12 |  | DFLPTTPGHSPGAGH |  |
| EuCA\_ctg\_023070\_58\_246 | 1 | 7.43e-12 |  | AFRPTSPGHSSKVGH |  |
| CaCA\_lg\_10\_34380\_144 | 1 | 7.43e-12 |  | GFKPTDPGHSPGVGH |  |
| BeVU\_chr\_6.sca003\_7722\_111 | 1 | 7.43e-12 |  | DFRPTAPGRSPGAGH |  |
| SpOL\_scf\_25714\_168\_336 | 1 | 8.07e-12 |  | DFRPTTPGDSPGAGH |  |
| NeNU\_scf\_00003\_13710\_87 | 1 | 8.07e-12 |  | FFRPTRPGHSPGVGH |  |
| LoJA\_chr\_2\_54742\_107 | 1 | 8.07e-12 |  | AFRPTTHGHSPGVGH |  |
| EuGR\_scf\_8\_143712\_190 | 1 | 8.07e-12 |  | DFRPTAPGSSPGLGH |  |
| EuGR\_scf\_8\_143467\_172 | 1 | 8.07e-12 |  | DFRPTAPGSSPGLGH |  |
| EuCA\_ctg\_011137\_153\_190 | 1 | 8.07e-12 |  | DFRPTAPGSSPGLGH |  |
| EuCA\_ctg\_007672\_9\_131 | 1 | 8.07e-12 |  | DFRPTAPGSSPGLGH |  |
| ViAN\_scf\_5923\_20\_108 | 1 | 8.83e-12 |  | DFQPTDPGHSPGAGH |  |
| PhVU\_chr\_09\_158890\_94 | 1 | 8.83e-12 |  | DFQPTDPGHSPGAGH |  |
| MeTR\_scf\_ac233112.6\_181\_148 | 1 | 8.83e-12 |  | AFKPTYPNHSPGVGH |  |
| LuAN\_scf\_13705\_12\_114 | 1 | 8.83e-12 |  | AFRPTSPGGSPGVGH |  |
| LeAL\_scf\_217\_537\_99 | 1 | 8.83e-12 |  | DFAPTTPGNSPGVGH |  |
| GlMA\_chr\_06\_276641\_130 | 1 | 8.83e-12 |  | DFQPTDPGHSPGAGH |  |
| FrEX\_scf\_2186\_525\_73 | 1 | 8.83e-12 |  | SFRPTTPGHSPSIGH |  |
| EuSA\_scf\_16\_7797\_147 | 1 | 8.83e-12 |  | DFAPTTPGNSPGVGH |  |
| CaCA\_lg\_07\_34851\_94 | 1 | 8.83e-12 |  | DFQPTDPGHSPGAGH |  |
| NeNU\_scf\_00099\_7938\_71 | 1 | 9.59e-12 |  | EIRPTIPGHSPGIGH |  |
| DiCA\_scf\_477\_135\_66 | 1 | 9.59e-12 |  | NFRPTNPGKSPGAGH |  |
| DiCA\_scf\_204\_432\_65 | 1 | 9.59e-12 |  | NFRPTNPGKSPGAGH |  |
| AeAR\_scf\_13\_713\_207 | 1 | 9.59e-12 |  | DFAPTNPGNSPGVGH |  |
| SeIN\_lg\_15\_9787\_116 | 1 | 1.04e-11 |  | GFQPTTPGHSPGVGH |  |
| GoRA\_chr\_07\_301859\_85 | 1 | 1.04e-11 |  | GVRPTKPGHSPGVGH |  |
| CaSA.PK\_scf\_3971\_17\_150 | 1 | 1.04e-11 |  | DIRPTAPGHSPGAGH |  |
| CaSA.Fi\_ctg\_10459586\_3\_126 | 1 | 1.04e-11 |  | DIRPTAPGHSPGAGH |  |
| LiUS\_scf\_2416\_588\_244 | 1 | 1.13e-11 |  | GFRPTQPGHSPGAGH |  |
| LiUS\_scf\_1249\_1842\_242 | 1 | 1.13e-11 |  | GFRPTQPGHSPGAGH |  |
| ArLY\_scf\_2\_10720\_340 | 1 | 1.13e-11 |  | DFEPTNPGNSLGIGH |  |
| TaHA\_scf\_10\_1346\_102 | 1 | 1.23e-11 |  | GFQPTSPGNSPGIGH |  |
| SiIR\_scf\_20\_535\_212 | 1 | 1.23e-11 |  | DFAPTSPGNSPGMGH |  |
| ScPA\_chr\_5-1\_14490\_150 | 1 | 1.23e-11 |  | DFAPTSPGNSPGMGH |  |
| GoRA\_chr\_03\_227185\_102 | 1 | 1.23e-11 |  | DFRPTTPGNSPGSGH |  |
| EuSA\_scf\_16\_7797\_95 | 1 | 1.23e-11 |  | DFAPTSPGNSPGMGH |  |
| EuSA\_scf\_16\_7797\_199 | 1 | 1.23e-11 |  | DFAPTSPGNSPGMGH |  |
| DiCA\_scf\_1661\_256\_73 | 1 | 1.23e-11 |  | AFRPTAPGRSPGAGH |  |
| DiCA\_scf\_1239\_8\_73 | 1 | 1.23e-11 |  | AFRPTAPGRSPGAGH |  |
| CaRU\_scf\_5\_58226\_210 | 1 | 1.23e-11 |  | DFAPTSPGNSPGMGH |  |
| BrRA\_chr\_04\_29832\_96 | 1 | 1.23e-11 |  | TFRPTVPGNSPGIGH |  |
| BrRA\_chr\_01\_110059\_155 | 1 | 1.23e-11 |  | DFAPTSPGNSPGMGH |  |
| BeVU\_chr\_5.sca002\_29411\_110 | 1 | 1.23e-11 |  | AFRPTAPGRSPGAGH |  |
| ArTH\_chr\_3\_89083\_77 | 1 | 1.23e-11 |  | DFVPTSPGNSPGVGH |  |
| AmTR\_scf\_00240\_74\_90 | 1 | 1.23e-11 |  | DFRPTDPGHSHGVGH |  |
| OrSA.Ja\_chr\_03\_226607\_77 | 1 | 1.33e-11 |  | GVRPTNPGHSPGIGH |  |
| OrSA.In\_chr\_03\_245043\_77 | 1 | 1.33e-11 |  | GVRPTNPGHSPGIGH |  |
| OrBR\_chr\_3\_153092\_92 | 1 | 1.33e-11 |  | GVRPTNPGHSPGIGH |  |
| GlMA\_chr\_14\_273581\_86 | 1 | 1.33e-11 |  | DFRPMDPGHSPGAGH |  |
| ViAN\_scf\_14509\_12\_115 | 1 | 1.44e-11 |  | AFRPTTPGGSPGVGH |  |
| PhVU\_chr\_03\_128036\_114 | 1 | 1.44e-11 |  | AFRPTTPGGSPGVGH |  |
| PhVU\_chr\_03\_128027\_115 | 1 | 1.44e-11 |  | AFRPTTPGGSPGVGH |  |
| OrSA.Ja\_chr\_09\_68574\_75 | 1 | 1.44e-11 |  | DGRPTSPGHSPGIGN |  |
| OrSA.In\_chr\_09\_64645\_75 | 1 | 1.44e-11 |  | DGRPTSPGHSPGIGN |  |
| OrBR\_chr\_9\_30468\_70 | 1 | 1.44e-11 |  | DGRPTSPGHSPGIGN |  |
| OrBR\_chr\_3\_48668\_173 | 1 | 1.44e-11 |  | DARPTTPGHSPGAGH |  |
| GlMA\_chr\_17\_184446\_120 | 1 | 1.44e-11 |  | AFRPTTPGGSPGVGH |  |
| CaCA\_scf\_133302\_184\_96 | 1 | 1.44e-11 |  | AFRPTTPGGSPGVGH |  |
| AeAR\_scf\_13\_713\_51 | 1 | 1.44e-11 |  | DFTPTNPGNSPGIGH |  |
| ThCA.Ma\_chr\_8\_1804\_121 | 1 | 1.57e-11 |  | DFRPTPPGHSPGAGH |  |
| SpPO\_chr\_4\_56998\_171 | 1 | 1.57e-11 |  | GARPTEPGHSPGIGH |  |
| SeIN\_lg\_15\_9787\_77 | 1 | 1.57e-11 |  | SFQPTTPGNSPGIGH |  |
| EuGR\_scf\_9\_64376\_141 | 1 | 1.57e-11 |  | AFHPTNPGHSPGVGH |  |
| EuCA\_ctg\_045219\_166\_101 | 1 | 1.57e-11 |  | AFHPTNPGHSPGVGH |  |
| PoEU\_scf\_234.1\_203\_154 | 1 | 1.70e-11 |  | DFQPTTPSHSPGVGH |  |
| OrSA.Ja\_chr\_03\_64155\_114 | 1 | 1.70e-11 |  | DARPTAPGHSPGAGH |  |
| OrSA.In\_chr\_03\_68043\_104 | 1 | 1.70e-11 |  | DARPTAPGHSPGAGH |  |
| DiCA\_scf\_234\_262\_166 | 1 | 1.84e-11 |  | DFRPTAPGTSPGAGH |  |
| HeBR\_scf\_155851\_99\_81 | 1 | 2.00e-11 |  | DFRATAPVHSPGVGH |  |
| ElOL\_scf\_00733\_1061\_823 | 1 | 2.00e-11 |  | DAQPTTPGHSPGVGH |  |
| BrRA\_chr\_09\_146618\_208 | 1 | 2.00e-11 |  | DFAPTTPGNSPGMGH |  |
| ArTH\_chr\_3\_89083\_233 | 1 | 2.00e-11 |  | DFAPTTPGNSPGMGH |  |
| ArLY\_scf\_5\_79011\_220 | 1 | 2.00e-11 |  | DFAPTTPGNSPGMGH |  |
| SoBI\_chr\_01\_340892\_101 | 1 | 2.17e-11 |  | DVRPTNPSHSPGIGH |  |
| SoBI\_chr\_01\_340810\_85 | 1 | 2.17e-11 |  | DVRPTNPSHSPGIGH |  |
| SiIR\_scf\_8\_11146\_59 | 1 | 2.17e-11 |  | DFGPTSPGNSPGIGH |  |
| PaVI\_chr\_09b\_67367\_89 | 1 | 2.17e-11 |  | DVRPTNPSHSPGIGH |  |
| PaVI\_chr\_09b\_67367\_149 | 1 | 2.17e-11 |  | DVRPTNPSHSPGIGH |  |
| PaVI\_chr\_09a\_177585\_81 | 1 | 2.17e-11 |  | DVRPTNPSHSPGIGH |  |
| PaVI\_chr\_09a\_177585\_140 | 1 | 2.17e-11 |  | DVRPTNPSHSPGIGH |  |
| PaHA\_scf\_147\_2797\_61 | 1 | 2.17e-11 |  | DVRPTNPSHSPGIGH |  |
| PaHA\_scf\_147\_2797\_121 | 1 | 2.17e-11 |  | DVRPTNPSHSPGIGH |  |
| LeAL\_scf\_72\_10083\_80 | 1 | 2.17e-11 |  | DFGPTSPGNSPGIGH |  |
| GlMA\_chr\_17\_184765\_74 | 1 | 2.17e-11 |  | DFRPMTPRHSPGVGH |  |
| EuSA\_scf\_6\_1164\_58 | 1 | 2.17e-11 |  | DFGPTSPGNSPGIGH |  |
| EuCA\_ctg\_023070\_58\_136 | 1 | 2.17e-11 |  | AFRPTSPGHSPGARH |  |
| BrRA\_chr\_07\_115013\_55 | 1 | 2.17e-11 |  | DFGPTSPGNSPGIGH |  |
| ArTH\_chr\_5\_82449\_59 | 1 | 2.17e-11 |  | DFGPTSPGNSPGIGH |  |
| ArLY\_scf\_8\_63500\_61 | 1 | 2.17e-11 |  | DFGPTSPGNSPGIGH |  |
| AmTR\_scf\_00240\_313\_63 | 1 | 2.17e-11 |  | DFRPTDPRLSPGVGH |  |
| AeAR\_scf\_13\_713\_155 | 1 | 2.17e-11 |  | DFAPTNPGNSPGMGH |  |
| LeAL\_scf\_217\_537\_151 | 1 | 2.34e-11 |  | DFAPTAPGNSPGMGH |  |
| ZeMA\_chr\_1\_871253\_86 | 1 | 2.54e-11 |  | DSRPTAPGNSPGIGN |  |
| SoBI\_chr\_07\_206797\_80 | 1 | 2.54e-11 |  | DSRPTAPGNSPGIGN |  |
| SeIT\_chr\_6\_126145\_188 | 1 | 2.54e-11 |  | DSRPTAPGNSPGIGN |  |
| PhHE\_scf\_01000559\_4478\_77 | 1 | 2.54e-11 |  | DSRPTAPGNSPGIGN |  |
| PaVI\_ctg\_356223\_8\_78 | 1 | 2.54e-11 |  | DSRPTAPGNSPGIGN |  |
| PaVI\_ctg\_173587\_8\_80 | 1 | 2.54e-11 |  | DSRPTAPGNSPGIGN |  |
| PaVI\_ctg\_05592\_97\_86 | 1 | 2.54e-11 |  | DSRPTAPGNSPGIGN |  |
| PaHA\_scf\_10\_16779\_92 | 1 | 2.54e-11 |  | DSRPTAPGNSPGIGN |  |
| OrSA.Ja\_chr\_08\_91083\_88 | 1 | 2.54e-11 |  | DSRPTAPGNSPGIGN |  |
| OrSA.In\_chr\_08\_97569\_88 | 1 | 2.54e-11 |  | DSRPTAPGNSPGIGN |  |
| NiSY\_scf\_1546\_797\_82 | 1 | 2.54e-11 |  | AYQPSTPGHSPGIGH |  |
| NiBE\_scf\_465\_4465\_96 | 1 | 2.54e-11 |  | AYQPSTPGHSPGIGH |  |
| HeBR\_scf\_243691\_91\_279 | 1 | 2.54e-11 |  | GFRPTTPGFSPGVGH |  |
| FrEX\_scf\_233\_540\_141 | 1 | 2.54e-11 |  | DFRPTGPGHSPGFGH |  |
| BrRA\_chr\_01\_110059\_103 | 1 | 2.54e-11 |  | DFAPTAPGNSPGVGY |  |
| BeVU\_chr\_6.sca003\_7759\_239 | 1 | 2.54e-11 |  | DFRPTAPGSSPGAGH |  |
| ZeMA\_chr\_7\_545502\_109 | 1 | 2.75e-11 |  | DGRPTAPGHSPGIGN |  |
| ZeMA\_chr\_2\_813531\_110 | 1 | 2.75e-11 |  | DGRPTAPGHSPGIGN |  |
| UtGI\_scf\_00238\_177\_76 | 1 | 2.75e-11 |  | SFRPTRPGNSPGMGH |  |
| SoBI\_chr\_02\_233223\_75 | 1 | 2.75e-11 |  | DGRPTAPGHSPGIGN |  |
| SeIT\_chr\_2\_133426\_66 | 1 | 2.75e-11 |  | DGRPTAPGHSPGIGN |  |
| SeIT\_chr\_2\_133410\_71 | 1 | 2.75e-11 |  | DGRPTAPGHSPGIGN |  |
| PhHE\_scf\_01003054\_1356\_67 | 1 | 2.75e-11 |  | DGRPTAPGHSPGIGN |  |
| PaVI\_chr\_02b\_133813\_67 | 1 | 2.75e-11 |  | DGRPTAPGHSPGIGN |  |
| PaVI\_chr\_02a\_355916\_87 | 1 | 2.75e-11 |  | DGRPTAPGHSPGIGN |  |
| PaHA\_scf\_45\_6570\_68 | 1 | 2.75e-11 |  | DGRPTAPGHSPGIGN |  |
| MuBA\_chr\_6\_22482\_94 | 1 | 2.75e-11 |  | DTRPTTPGHSPGAGH |  |
| MuAC\_chr\_6\_24028\_94 | 1 | 2.75e-11 |  | DTRPTTPGHSPGAGH |  |
| MiGU\_scf\_8\_8701\_66 | 1 | 2.75e-11 |  | GFQPTTPGNSPGVGH |  |
| AmTR\_scf\_00157\_6341\_123 | 1 | 2.75e-11 |  | DSRSTSPGHSPGVGH |  |
| SeIT\_chr\_9\_300280\_83 | 1 | 2.97e-11 |  | DVRPTTPTHSPGIGH |  |
| SeIT\_chr\_9\_300249\_77 | 1 | 2.97e-11 |  | DVRPTTPTHSPGIGH |  |
| LiUS\_scf\_2416\_588\_142 | 1 | 2.97e-11 |  | GFRPTAPGFSPGVGH |  |
| LiUS\_scf\_1249\_1842\_139 | 1 | 2.97e-11 |  | GFRPTAPGFSPGVGH |  |
| GoRA\_chr\_03\_227119\_76 | 1 | 2.97e-11 |  | DFRPTAPGNSPGGGH |  |
| CiAR.Ka\_chr\_7\_28005\_109 | 1 | 2.97e-11 |  | GFKPTIPGHSPGVGH |  |
| PhHE\_scf\_01004822\_313\_89 | 1 | 3.21e-11 |  | DTRPTAPGHSPGAGH |  |
| MuAC\_chr\_7\_138912\_167 | 1 | 3.21e-11 |  | NVRPSTPGHSPGVGH |  |
| LeAL\_scf\_17\_1975\_95 | 1 | 3.21e-11 |  | DFRPTDHGNSPGVGH |  |
| EuCA\_ctg\_047439\_35\_90 | 1 | 3.47e-11 |  | NFRSTKRGHSPGVGH |  |
| ArLY\_scf\_8\_33647\_440 | 1 | 3.74e-11 |  | DFAPTNPGYSLGIGH |  |
| AmTR\_scf\_00676\_17\_63 | 1 | 3.74e-11 |  | DFRPADPRHSPGVGH |  |
| TaHA\_scf\_33\_10708\_72 | 1 | 4.04e-11 |  | GFEPTSPGNSPGVGH |  |
| MaES\_chr\_xviii\_47380\_659 | 1 | 4.04e-11 |  | DHRPTQPGHSPGVGH |  |
| GlMA\_chr\_16\_157456\_68 | 1 | 4.04e-11 |  | AFRPTCRGHSPGAGH |  |
| EuCA\_ctg\_018397\_212\_141 | 1 | 4.04e-11 |  | AFRSTNPGHSPRVGH |  |
| ArLY\_scf\_8\_33647\_190 | 1 | 4.04e-11 |  | DFAPTNPGNSLGIGH |  |
| ArLY\_scf\_2\_10720\_440 | 1 | 4.04e-11 |  | DFAPTNPGNSLGIGH |  |
| PoTR\_chr\_07\_72858\_120 | 1 | 4.36e-11 |  | DFKPITSGQSPGVGH |  |
| PoTR\_chr\_07\_72674\_120 | 1 | 4.36e-11 |  | DFKPITSGQSPGVGH |  |
| PiGL\_ctg\_165564112\_0\_3\_147 | 1 | 4.71e-11 |  | AFRPSSSGHSPGRGH |  |
| MeTR\_scf\_ac233112.6\_159\_73 | 1 | 4.71e-11 |  | SFRPTTPGSSPGVGH |  |
| JaCU\_scf\_02121\_40\_73 | 1 | 4.71e-11 |  | DFRPTTGGHSPGAGH |  |
| HeBR\_scf\_155851\_130\_120 | 1 | 4.71e-11 |  | EFRPTATGDSPGVGH |  |
| CiAR.Ka\_chr\_7\_28005\_62 | 1 | 4.71e-11 |  | SFRPTTPGSSPGVGH |  |
| CaPA\_ctg\_55\_3031\_51 | 1 | 4.71e-11 |  | GFRPTTPGISPGVGH |  |
| SpOL\_scf\_21396\_72\_78 | 1 | 5.07e-11 |  | DFRPTSPGGSPGAGH |  |
| SpOL\_scf\_21396\_72\_445 | 1 | 5.07e-11 |  | DFRPTSPGGSPGAGH |  |
| PiGL\_ctg\_30784271\_0\_25\_136 | 1 | 5.07e-11 |  | AFRPSSSSHSPGIGH |  |
| AmTR\_scf\_00270\_48\_148 | 1 | 5.46e-11 |  | NFRPTDLRHSPGVGH |  |
| SpOL\_scf\_25714\_168\_284 | 1 | 5.89e-11 |  | DFRHTTPGHSPGAGH |  |
| MeTR\_chr\_5\_33061\_42 | 1 | 6.34e-11 |  | AFRPTPSGHSLGVGH |  |
| CaRU\_scf\_5\_58226\_106 | 1 | 6.34e-11 |  | DFAPTFPGHSPGVGH |  |
| PyCO\_scf\_00016\_3634\_132 | 1 | 6.83e-11 |  | AGKPTAPGHSPGVGH |  |
| PyBR\_scf\_4.0\_9791\_132 | 1 | 6.83e-11 |  | AGKPTAPGHSPGVGH |  |
| MaDO\_chr\_2\_149034\_127 | 1 | 6.83e-11 |  | AGKPTAPGHSPGVGH |  |
| EuGR\_scf\_8\_418581\_165 | 1 | 7.36e-11 |  | DFEATRPGHSPGVGH |  |
| EuCA\_ctg\_034442\_93\_149 | 1 | 7.36e-11 |  | DFEATRPGHSPGVGH |  |
| PoEU\_scf\_623.1\_696\_139 | 1 | 7.92e-11 |  | DFQPTTPGRSPGAGH |  |
| PoEU\_scf\_354.1\_893\_139 | 1 | 7.92e-11 |  | DFQPTTPGRSPGAGH |  |
| PaVI\_chr\_02b\_133822\_68 | 1 | 7.92e-11 |  | DGRPTAPGHSPGMGN |  |
| EuGR\_scf\_9\_64047\_93 | 1 | 7.92e-11 |  | DFPSTEPGHSPGVGH |  |
| EuCA\_ctg\_050450\_46\_93 | 1 | 7.92e-11 |  | DFPSTEPGHSPGVGH |  |
| PrPE\_scf\_7\_52252\_171 | 1 | 8.53e-11 |  | GTRPTAPGHSPGVGH |  |
| PrMU\_chr\_8\_31248\_131 | 1 | 8.53e-11 |  | GTRPTAPGHSPGVGH |  |
| PiTA\_scf\_585603\_36\_135 | 1 | 8.53e-11 |  | GFRPTSSSHSPGVGH |  |
| NeNU\_scf\_00099\_7001\_151 | 1 | 9.18e-11 |  | AFRPTAPSHSFGIGH |  |
| CaCH\_scf\_110772\_34\_125 | 1 | 9.18e-11 |  | DFQPSGPGHSPGVGH |  |
| CaAN\_chr\_02\_414050\_125 | 1 | 9.18e-11 |  | DFQPSGPGHSPGVGH |  |
| DiCA\_scf\_82\_1375\_144 | 1 | 9.89e-11 |  | GFRPTSPGRSPGAGH |  |
| BrRA\_chr\_04\_76977\_93 | 1 | 9.89e-11 |  | AYAPTDPGNSPGIGH |  |
| BrRA\_chr\_04\_76977\_174 | 1 | 9.89e-11 |  | AYAPTDPGNSPGIGH |  |
| BrRA\_chr\_04\_76968\_93 | 1 | 9.89e-11 |  | AYAPTDPGNSPGIGH |  |
| AmTR\_scf\_00157\_6321\_111 | 1 | 9.89e-11 |  | NSRSTSPGHSPGVGH |  |
| AzIN\_scf\_2043\_2\_19\_119 | 1 | 1.07e-10 |  | DFLPTKSGSSPGIGH |  |
| AzIN\_scf\_18451\_1\_5\_139 | 1 | 1.07e-10 |  | DFLPTKSGSSPGIGH |  |
| AzIN\_scf\_18451\_1\_5\_104 | 1 | 1.07e-10 |  | DFLPTKSGSSPGIGH |  |
| BrRA\_chr\_09\_208495\_60 | 1 | 1.15e-10 |  | DFGPTYPGNSPGIGH |  |
| EuGR\_scf\_8\_418665\_133 | 1 | 1.23e-10 |  | DFRPTTPGSSPRVGH |  |
| CaCA\_lg\_06\_45369\_132 | 1 | 1.23e-10 |  | GFSPTEPGHSPGVGH |  |
| AbSI\_ctg\_3356250\_5\_137 | 1 | 1.23e-10 |  | AFRPASSGNSPGRGH |  |
| MeTR\_scf\_ac233112.6\_159\_120 | 1 | 1.33e-10 |  | GFKPTNPSHSPGVGH |  |
| SpOL\_scf\_33414\_32\_87 | 1 | 1.43e-10 |  | SFRPTAPGRSPGAGH |  |
| EuSA\_scf\_5\_91852\_71 | 1 | 1.43e-10 |  | AFRPTLPGPSQGIGH |  |
| ElGU\_chr\_15\_77587\_117 | 1 | 1.43e-10 |  | DARPTTPGHGPGVGH |  |
| BeVU\_chr\_6.sca003\_7802\_97 | 1 | 1.43e-10 |  | SFRPTAPGRSPGAGH |  |
| MuBA\_chr\_7\_123907\_170 | 1 | 1.53e-10 |  | SVRPSTPGHSPGVGH |  |
| BrDI\_chr\_3\_355044\_70 | 1 | 1.53e-10 |  | DSRSTSPGNSPGIGN |  |
| BrDI\_chr\_1\_269090\_87 | 1 | 1.53e-10 |  | GVRPSNPGHSPGIGH |  |
| DiCA\_scf\_234\_262\_202 | 1 | 1.65e-10 |  | EFQPTAPGDSPGAGH |  |
| AmTR\_scf\_00157\_302\_75 | 1 | 1.65e-10 |  | NFSPTAPGHSLGIGH |  |
| SpOL\_scf\_25714\_134\_121 | 1 | 1.77e-10 |  | GFRPTAPGRSPGAGH |  |
| MaES\_chr\_x\_67218\_114 | 1 | 1.77e-10 |  | AFLPTTPGHSSGVGN |  |
| DiCA\_scf\_234\_290\_213 | 1 | 1.77e-10 |  | SFRPTAPGDSPGAGH |  |
| ArLY\_scf\_2\_10720\_265 | 1 | 1.77e-10 |  | DFAPTNPGHSPGIRH |  |
| SeIT\_chr\_9\_170607\_82 | 1 | 1.90e-10 |  | DVRPSTPRHSPGIGH |  |
| MuBA\_chr\_9\_55857\_168 | 1 | 2.04e-10 |  | AQQPTSPGHSPSVGH |  |
| EnVE\_ctg\_22030\_27790\_1\_28\_176 | 1 | 2.04e-10 |  | AQQPTSPGHSPSVGH |  |
| SoTU\_chr\_03\_273390\_149 | 1 | 2.20e-10 |  | DFGPTGPGHSPGIGH |  |
| SoPI\_ctg\_870016\_5\_103 | 1 | 2.20e-10 |  | DFGPTGPGHSPGIGH |  |
| SoLY\_chr\_03\_288882\_103 | 1 | 2.20e-10 |  | DFGPTGPGHSPGIGH |  |
| PiGL\_ctg\_165043132\_0\_3\_153 | 1 | 2.20e-10 |  | AFRPSSSGPSPGIGH |  |
| DiCA\_scf\_234\_290\_177 | 1 | 2.20e-10 |  | DFQPTAPGISPGAGH |  |
| ArLY\_scf\_8\_33647\_390 | 1 | 2.20e-10 |  | DFAPTNPRNSPGIGH |  |
| PiTA\_scf\_t7716\_19726\_147 | 1 | 2.36e-10 |  | AFTPSSSGHSPGIGH |  |
| PiTA\_scf\_t5839\_2326\_171 | 1 | 2.36e-10 |  | AFTPSSSGHSPGIGH |  |
| PiTA\_scf\_t2391\_529\_171 | 1 | 2.36e-10 |  | AFTPSSSGHSPGIGH |  |
| PiTA\_scf\_44548\_262\_148 | 1 | 2.36e-10 |  | AFTPSSSGHSPGIGH |  |
| PiTA\_scf\_365587\_807\_171 | 1 | 2.36e-10 |  | AFTPSSSGHSPGIGH |  |
| PiTA\_ctg\_32464818\_318\_171 | 1 | 2.36e-10 |  | AFTPSSSGHSPGIGH |  |
| PaVI\_chr\_02a\_355897\_71 | 1 | 2.36e-10 |  | DGRATNPGHSPGIGN |  |
| FrEX\_scf\_14360\_8\_221 | 1 | 2.36e-10 |  | GFRPTESGPSPGIGH |  |
| EuGR\_scf\_9\_64228\_109 | 1 | 2.36e-10 |  | AIHPTKPGHSPGVGH |  |
| MaDO\_chr\_15\_179591\_143 | 1 | 2.53e-10 |  | AGRPTGPGHSPGVGH |  |
| BeVU\_chr\_6.sca003\_7759\_186 | 1 | 2.53e-10 |  | DFRRTTPGRSPGAGH |  |
| PhHE\_scf\_01001027\_375\_77 | 1 | 2.92e-10 |  | DGRSTAPGHSPGIGN |  |
| MuBA\_chr\_9\_55857\_148 | 1 | 2.92e-10 |  | WQQPTSPGHSPSIGH |  |
| MuAC\_chr\_9\_62965\_148 | 1 | 2.92e-10 |  | WQQPTSPGHSPSIGH |  |
| EnVE\_ctg\_22030\_27790\_1\_28\_156 | 1 | 2.92e-10 |  | WQQPTSPGHSPSIGH |  |
| BrDI\_chr\_4\_169807\_80 | 1 | 2.92e-10 |  | DGRSTAPGHSPGIGN |  |
| EnVE\_scf\_22030\_78542\_116\_48 | 1 | 3.13e-10 |  | WLPPTSQGHSPGVGH |  |
| CaRU\_scf\_5\_58226\_54 | 1 | 3.13e-10 |  | DFVPTSLGNSLGVGH |  |
| ThCA.Ma\_chr\_1\_202452\_84 | 1 | 3.36e-10 |  | DFQLTKPGPSPGVGH |  |
| ThCA.Cr\_chr\_01\_161913\_84 | 1 | 3.36e-10 |  | DFQLTKPGPSPGVGH |  |
| EuSA\_scf\_5\_91709\_95 | 1 | 3.36e-10 |  | AFRPTVPGPSQGIGH |  |
| PaHA\_scf\_45\_6582\_63 | 1 | 3.61e-10 |  | DSRPTPPGHSPGIGN |  |
| EuCA\_ctg\_023562\_313\_134 | 1 | 3.87e-10 |  | HFRPTTPGTSPGVGN |  |
| DiCA\_scf\_234\_274\_102 | 1 | 3.87e-10 |  | TFRPTAPGTSPGAGH |  |
| DiCA\_scf\_234\_252\_101 | 1 | 3.87e-10 |  | TFRPTAPGTSPGAGH |  |
| CaRU\_scf\_8\_42175\_60 | 1 | 4.15e-10 |  | SIKPTTPGHSQGVGH |  |
| BrRA\_chr\_09\_146618\_52 | 1 | 4.15e-10 |  | EFVPSSPGNSPGMGH |  |
| ArTH\_chr\_1\_42237\_161 | 1 | 4.15e-10 |  | DFAPTNPGNSPGIRH |  |
| ScPA\_chr\_2-4\_32069\_67 | 1 | 4.44e-10 |  | GFGPTSPGNSPGIGH |  |
| TaHA\_scf\_21\_4981\_72 | 1 | 4.76e-10 |  | AFRPTYQGPSQGIGH |  |
| SpOL\_scf\_21396\_72\_349 | 1 | 4.76e-10 |  | DFRPTSPGGSPGDGH |  |
| SpOL\_scf\_21396\_72\_174 | 1 | 4.76e-10 |  | DFRPTSPGGSPGDGH |  |
| NiBE\_scf\_2639\_1487\_70 | 1 | 4.76e-10 |  | SFKPSGPGHSPGVGH |  |
| EuGR\_scf\_8\_418665\_177 | 1 | 4.76e-10 |  | DFEGTRPGHSPGVGH |  |
| EuGR\_scf\_8\_143712\_234 | 1 | 4.76e-10 |  | DFEGTRPGHSPGVGH |  |
| EuGR\_scf\_8\_143467\_216 | 1 | 4.76e-10 |  | DFEGTRPGHSPGVGH |  |
| EuCA\_ctg\_023562\_184\_177 | 1 | 4.76e-10 |  | DFEGTRPGHSPGVGH |  |
| EuCA\_ctg\_011137\_153\_234 | 1 | 4.76e-10 |  | DFEGTRPGHSPGVGH |  |
| EuCA\_ctg\_007672\_9\_159 | 1 | 4.76e-10 |  | DFEGTRPGHSPGVGH |  |
| BrDI\_chr\_1\_402157\_80 | 1 | 4.76e-10 |  | DSRPTGPGHSPGAGH |  |
| AeAR\_scf\_9\_3455\_78 | 1 | 4.76e-10 |  | AFRPTYQGPSQGIGH |  |
| CaAN\_chr\_02\_414002\_77 | 1 | 5.10e-10 |  | KYAPTSTGNSPGIGH |  |
| SoBI\_chr\_01\_340955\_81 | 1 | 5.46e-10 |  | DTRATDPGHSPGAGH |  |
| SeIT\_chr\_9\_300311\_79 | 1 | 5.46e-10 |  | DTRATDPGHSPGAGH |  |
| PaVI\_ctg\_85087\_29\_82 | 1 | 5.46e-10 |  | DTRATDPGHSPGAGH |  |
| PaVI\_chr\_09b\_67384\_118 | 1 | 5.46e-10 |  | DTRATDPGHSPGAGH |  |
| PaHA\_scf\_147\_2812\_80 | 1 | 5.46e-10 |  | DTRATDPGHSPGAGH |  |
| AmTR\_scf\_00240\_74\_63 | 1 | 5.46e-10 |  | DFRPTDPHHSSGVGH |  |
| TrUR\_scf\_38417\_104\_81 | 1 | 6.26e-10 |  | DSRGTAPGHSPGIGN |  |
| MeTR\_chr\_5\_33063\_115 | 1 | 6.26e-10 |  | AFRPTPPGHSPGGGH |  |
| HoVU\_ctg\_144873\_28\_180 | 1 | 6.26e-10 |  | DSRGTAPGHSPGIGN |  |
| EuCA\_ctg\_018397\_20\_217 | 1 | 6.26e-10 |  | AFRPTSPGHNPGARH |  |
| AeTA\_scf\_3881\_517\_91 | 1 | 6.26e-10 |  | DSRGTAPGHSPGIGN |  |
| SiIR\_scf\_43\_14573\_148 | 1 | 7.17e-10 |  | AFRPTGPGPSQGIGH |  |
| ScPA\_chr\_1-1\_34613\_59 | 1 | 7.17e-10 |  | AFRPTGPGPSQGIGH |  |
| MiGU\_scf\_8\_8739\_79 | 1 | 7.17e-10 |  | DSKSTVPGHSPGIGH |  |
| DiCA\_scf\_6523\_40\_104 | 1 | 7.17e-10 |  | RFRPTMPGTSPGAGH |  |
| DiCA\_scf\_234\_310\_104 | 1 | 7.17e-10 |  | RFRPTMPGTSPGAGH |  |
| BrRA\_chr\_08\_95395\_224 | 1 | 7.17e-10 |  | AFRPTGPGPSQGIGH |  |
| PyCO\_scf\_03185\_22\_133 | 1 | 8.20e-10 |  | AGKPTGPGHSPGVGH |  |
| PyBR\_scf\_520.0\_2943\_133 | 1 | 8.20e-10 |  | AGKPTGPGHSPGVGH |  |
| CaCH\_scf\_102917\_258\_77 | 1 | 8.77e-10 |  | KYAPTATGNSPGIGH |  |
| EuGR\_scf\_8\_418753\_166 | 1 | 9.38e-10 |  | EFSSTRPGHSPGVGH |  |
| EuCA\_ctg\_023562\_269\_166 | 1 | 9.38e-10 |  | EFSSTRPGHSPGVGH |  |
| SiIR\_scf\_20\_535\_56 | 1 | 1.07e-09 |  | EFVPTSPGSSPGLGH |  |
| SpOL\_scf\_21396\_72\_509 | 1 | 1.15e-09 |  | DFRPTSPGGSPGGGH |  |
| SpOL\_scf\_21396\_72\_477 | 1 | 1.15e-09 |  | DFRPTSPGGSPGGGH |  |
| SpOL\_scf\_21396\_72\_413 | 1 | 1.15e-09 |  | DFRPTSPGGSPGGGH |  |
| SpOL\_scf\_21396\_72\_317 | 1 | 1.15e-09 |  | DFRPTSPGGSPGGGH |  |
| SpOL\_scf\_21396\_72\_285 | 1 | 1.15e-09 |  | DFRPTSPGGSPGGGH |  |
| SpOL\_scf\_21396\_72\_238 | 1 | 1.15e-09 |  | DFRPTSPGGSPGGGH |  |
| SpOL\_scf\_21396\_72\_206 | 1 | 1.15e-09 |  | DFRPTSPGGSPGGGH |  |
| SpOL\_scf\_21396\_72\_142 | 1 | 1.15e-09 |  | DFRPTSPGGSPGGGH |  |
| SpOL\_scf\_21396\_72\_110 | 1 | 1.15e-09 |  | DFRPTSPGGSPGGGH |  |
| NiTO\_scf\_15877\_27\_88 | 1 | 1.15e-09 |  | SFHPSTPGHSPGIGH |  |
| NiSY\_scf\_161423\_75\_80 | 1 | 1.15e-09 |  | SFHPSTPGHSPGIGH |  |
| NiBE\_scf\_7636\_341\_80 | 1 | 1.15e-09 |  | SFHPSTPGHSPGIGH |  |
| NiBE\_scf\_606\_2401\_80 | 1 | 1.15e-09 |  | SFHPSTPGHSPGIGH |  |
| CaCH\_scf\_44597\_8\_79 | 1 | 1.22e-09 |  | SFHPATPGHSPGIGH |  |
| CaAN\_chr\_02\_42595\_79 | 1 | 1.22e-09 |  | SFHPATPGHSPGIGH |  |
| AmTR\_scf\_00270\_48\_229 | 1 | 1.22e-09 |  | DFRPTRSGPSPGAGH |  |
| ThCA.Ma\_chr\_8\_113480\_99 | 1 | 1.40e-09 |  | DVHPTSPGHSPSVGH |  |
| ThCA.Cr\_chr\_08\_64055\_99 | 1 | 1.40e-09 |  | DVHPTSPGHSPSVGH |  |
| NiTO\_scf\_10718\_593\_73 | 1 | 1.59e-09 |  | GFQPSGPGHSPGVGH |  |
| NiBE\_scf\_465\_4629\_89 | 1 | 1.59e-09 |  | GFQPSGPGHSPGVGH |  |
| EuGR\_scf\_6\_1772\_116 | 1 | 1.70e-09 |  | DQWSTEPGHSPGAGH |  |
| EuCA\_ctg\_050958\_143\_116 | 1 | 1.70e-09 |  | DQWSTEPGHSPGAGH |  |
| EuCA\_ctg\_023293\_14\_116 | 1 | 1.70e-09 |  | DQWSTEPGHSPGAGH |  |
| ElGU\_chr\_15\_38349\_1292 | 1 | 1.81e-09 |  | DGGPTEPGHSPGVGH |  |
| TrUR\_scf\_73978\_126\_81 | 1 | 1.94e-09 |  | ESRGTAPGNSPGIGN |  |
| HoVU\_ctg\_96118\_25\_175 | 1 | 1.94e-09 |  | ESRGTAPGNSPGIGN |  |
| EuGR\_scf\_6\_1772\_95 | 1 | 1.94e-09 |  | DSESTDPGHSPGVGH |  |
| EuCA\_ctg\_055172\_400\_184 | 1 | 1.94e-09 |  | GFKPPSPGHSPGIGN |  |
| EuCA\_ctg\_050958\_143\_95 | 1 | 1.94e-09 |  | DSESTDPGHSPGVGH |  |
| EuCA\_ctg\_031336\_177\_140 | 1 | 1.94e-09 |  | VFKPPTPGHSPGIGN |  |
| EuCA\_ctg\_023293\_14\_95 | 1 | 1.94e-09 |  | DSESTDPGHSPGVGH |  |
| CaRU\_scf\_1\_36405\_85 | 1 | 1.94e-09 |  | AFRPTGQGPSQGIGH |  |
| ArTH\_chr\_1\_36231\_73 | 1 | 1.94e-09 |  | AFRPTGQGPSQGIGH |  |
| AeTA\_scf\_31116\_316\_81 | 1 | 1.94e-09 |  | ESRGTAPGNSPGIGN |  |
| NiTO\_scf\_3364\_1611\_90 | 1 | 2.07e-09 |  | SFHPVTPGHSPGIGH |  |
| EuGR\_scf\_6\_192772\_87 | 1 | 2.20e-09 |  | GFRPTIPGPSPGIGN |  |
| PoEU\_scf\_234.1\_203\_228 | 1 | 2.35e-09 |  | ELSVTTPGQSPGVGH |  |
| GoRA\_chr\_03\_227254\_151 | 1 | 2.35e-09 |  | GFRHTTPGNSPGAGH |  |
| DiCA\_scf\_6587\_48\_100 | 1 | 2.35e-09 |  | RYRPTMPGTSPGAGH |  |
| LiUS\_scf\_464\_9234\_106 | 1 | 2.51e-09 |  | AFRPTAPGHGSAVGH |  |
| OrBR\_chr\_7\_105103\_82 | 1 | 2.67e-09 |  | DSLPTNPGHSPGTGH |  |
| ArTH\_chr\_1\_42237\_111 | 1 | 2.67e-09 |  | DFAPTNPGHNSGIGH |  |
| ArTH\_chr\_1\_118662\_119 | 1 | 2.67e-09 |  | EFAPTNPEDSLGIGH |  |
| MuBA\_chr\_1\_126682\_67 | 1 | 2.85e-09 |  | WQAPTNPGHSPSIGH |  |
| MuAC\_chr\_1\_141422\_67 | 1 | 2.85e-09 |  | WQAPTNPGHSPSIGH |  |
| ArTH\_chr\_1\_118662\_144 | 1 | 3.04e-09 |  | DFAPTNPGDSPGIRH |  |
| TrUR\_scf\_69099\_94\_65 | 1 | 3.24e-09 |  | QVESTTPGHSPSIGH |  |
| HoVU\_ctg\_853286\_22\_86 | 1 | 3.24e-09 |  | QVESTTPGHSPSIGH |  |
| AeTA\_scf\_7016\_496\_65 | 1 | 3.24e-09 |  | QVESTTPGHSPSIGH |  |
| NiSY\_scf\_35720\_248\_113 | 1 | 3.45e-09 |  | GFRRSGPGHSPGIGH |  |
| NiBE\_scf\_1697\_919\_107 | 1 | 3.45e-09 |  | GFRRSGPGHSPGIGH |  |
| AmTR\_scf\_00011\_26680\_111 | 1 | 3.45e-09 |  | DTPPTSPGHSPGAGH |  |
| TaHA\_scf\_2\_25572\_95 | 1 | 3.68e-09 |  | AFRPTHQGPSQGIGH |  |
| SiIR\_scf\_1042\_47125\_77 | 1 | 3.68e-09 |  | AFRPTHQGPSQGIGH |  |
| ScPA\_chr\_4-6\_41886\_75 | 1 | 3.68e-09 |  | AFRPTHQGPSQGIGH |  |
| LeAL\_scf\_38\_774\_88 | 1 | 3.68e-09 |  | AFRPTHQGPSQGIGH |  |
| LeAL\_scf\_26\_8556\_79 | 1 | 3.68e-09 |  | AFRPTHQGPSQGIGH |  |
| EuSA\_scf\_10\_49408\_67 | 1 | 3.68e-09 |  | AFRPTHQGPSQGIGH |  |
| CaRU\_scf\_4\_63114\_71 | 1 | 3.68e-09 |  | AFRPTHQGPSQGIGH |  |
| BrRA\_chr\_05\_14020\_76 | 1 | 3.68e-09 |  | AFRPTHQGPSQGIGH |  |
| BrRA\_chr\_04\_64576\_78 | 1 | 3.68e-09 |  | AFRPTHQGPSQGIGH |  |
| BrRA\_chr\_03\_161871\_75 | 1 | 3.68e-09 |  | AFRPTHQGPSQGIGH |  |
| ArTH\_chr\_2\_76174\_76 | 1 | 3.68e-09 |  | AFRPTHQGPSQGIGH |  |
| ArLY\_scf\_4\_85025\_74 | 1 | 3.68e-09 |  | AFRPTHQGPSQGIGH |  |
| GlMA\_chr\_05\_53925\_133 | 1 | 3.92e-09 |  | AFRSTTPGGSTGVGH |  |
| NiTO\_scf\_16588\_177\_82 | 1 | 4.44e-09 |  | DYPPIKPGHSPGMGN |  |
| NiSY\_scf\_26250\_274\_88 | 1 | 4.44e-09 |  | DYPPIKPGHSPGMGN |  |
| NiBE\_scf\_118\_3224\_96 | 1 | 4.44e-09 |  | DYPPIKPGHSPGMGN |  |
| ElOL\_scf\_00542\_907\_90 | 1 | 4.73e-09 |  | DSLPTNPGHGPGVGH |  |
| ElGU\_chr\_11\_150068\_67 | 1 | 4.73e-09 |  | DSLPTNPGHGPGVGH |  |
| PhDA\_scf\_000143\_2896\_73 | 1 | 5.03e-09 |  | DNLPTNPGHGPGVGH |  |
| NiBE\_scf\_126\_1778\_83 | 1 | 5.03e-09 |  | SFHPVTPGHSPGMGH |  |
| DiCA\_scf\_234\_290\_255 | 1 | 6.07e-09 |  | DYQPTPPGHSPQAGH |  |
| PhDA\_scf\_001357\_165\_88 | 1 | 6.45e-09 |  | GDRPSVPGHSPGVGH |  |
| OrSA.Ja\_chr\_06\_127008\_73 | 1 | 6.87e-09 |  | QVDSTTPGHSPSIGH |  |
| OrSA.In\_chr\_06\_125382\_73 | 1 | 6.87e-09 |  | QVDSTTPGHSPSIGH |  |
| OrGL\_chr\_06\_44603\_73 | 1 | 6.87e-09 |  | QVDSTTPGHSPSIGH |  |
| OrBR\_chr\_6\_74540\_95 | 1 | 6.87e-09 |  | QVDSTTPGHSPSIGH |  |
| MeTR\_chr\_5\_33063\_62 | 1 | 8.25e-09 |  | AFRPNPPGHSPGGGH |  |
| CaCH\_scf\_179831\_16\_82 | 1 | 8.77e-09 |  | DIPPVTPGHSPGMGH |  |
| CaAN\_chr\_12\_556914\_82 | 1 | 8.77e-09 |  | DIPPVTPGHSPGMGH |  |
| FrEX\_scf\_7878\_96\_103 | 1 | 9.91e-09 |  | GPRTTAPGHSPGIGH |  |
| ZeMA\_chr\_5\_1636826\_68 | 1 | 1.05e-08 |  | QTESTTPGHSPSIGH |  |
| SoBI\_chr\_10\_225742\_72 | 1 | 1.05e-08 |  | QTESTTPGHSPSIGH |  |
| ElOL\_scf\_00904\_643\_119 | 1 | 1.05e-08 |  | DGGPTEPGHSPGAGH |  |
| NiTO\_scf\_31316\_86\_110 | 1 | 1.19e-08 |  | GFTPSGPGHSPGIGH |  |
| NiSY\_scf\_25424\_227\_110 | 1 | 1.19e-08 |  | GFTPSGPGHSPGIGH |  |
| NiBE\_scf\_465\_3928\_111 | 1 | 1.19e-08 |  | GFTPSGPGHSPGIGH |  |
| EuGR\_scf\_6\_1947\_119 | 1 | 1.26e-08 |  | DRWSTEPGHSPGAGH |  |
| EuGR\_scf\_566\_96\_119 | 1 | 1.26e-08 |  | DRWSTEPGHSPGAGH |  |
| EuCA\_ctg\_046052\_114\_119 | 1 | 1.26e-08 |  | DRWSTEPGHSPGAGH |  |
| EuCA\_ctg\_014037\_37\_119 | 1 | 1.26e-08 |  | DRWSTEPGHSPGAGH |  |
| EuCA\_ctg\_007856\_3\_119 | 1 | 1.26e-08 |  | DRWSTEPGHSPGAGH |  |
| PaHA\_scf\_31\_12006\_66 | 1 | 1.34e-08 |  | QADSTTPGHSPSIGH |  |
| EuCA\_ctg\_023562\_184\_133 | 1 | 1.34e-08 |  | DFHPTTPGSSPRVGH |  |
| DiCA\_scf\_234\_262\_244 | 1 | 1.34e-08 |  | DYRSTGPGQSPRAGH |  |
| AeTA\_scf\_55155\_109\_136 | 1 | 1.34e-08 |  | DGRGTGPGHSPGIGN |  |
| MeTR\_chr\_5\_33061\_94 | 1 | 1.70e-08 |  | AFRPTPPGHSPGGGT |  |
| PyCO\_scf\_33884\_6\_76 | 1 | 2.03e-08 |  | SHRSTTPGHSPGAGH |  |
| PyBR\_scf\_154.0\_460\_76 | 1 | 2.03e-08 |  | SHRSTTPGHSPGAGH |  |
| MaDO\_chr\_15\_8403\_76 | 1 | 2.03e-08 |  | SHRSTTPGHSPGAGH |  |
| SeIT\_chr\_4\_167625\_86 | 1 | 2.15e-08 |  | QTDSTTPGHSPSIGH |  |
| PaVI\_ctg\_16368\_16\_66 | 1 | 2.15e-08 |  | QTDSTTPGHSPSIGH |  |
| PhVU\_chr\_03\_128027\_147 | 1 | 2.56e-08 |  | DSKSEGPGHSPGVGH |  |
| ArLY\_scf\_8\_33647\_115 | 1 | 3.05e-08 |  | DFATTNPGDSSGIGH |  |
| SoTU\_chr\_12\_140322\_102 | 1 | 3.23e-08 |  | DIPYVTPGHSPGMGH |  |
| SoPI\_ctg\_6592119\_8\_84 | 1 | 3.23e-08 |  | DIPYVTPGHSPGMGH |  |
| SoLY\_chr\_12\_152273\_84 | 1 | 3.23e-08 |  | DIPYVTPGHSPGMGH |  |
| HoVU\_ctg\_1982316\_28\_72 | 1 | 3.23e-08 |  | DGRGTGQGHSPGIGN |  |
| GlMA\_chr\_17\_184482\_126 | 1 | 3.23e-08 |  | AFQPTTPRGSPSVGH |  |
| ArLY\_scf\_2\_10720\_115 | 1 | 3.43e-08 |  | DFATTNPGDSFGIGH |  |
| EuGR\_scf\_8\_418547\_117 | 1 | 4.31e-08 |  | SLGSTSPGHSPSIGH |  |
| EuGR\_scf\_6\_108863\_103 | 1 | 4.83e-08 |  | LPEATDPGHSPGVGH |  |
| EuCA\_ctg\_013901\_36\_103 | 1 | 4.83e-08 |  | LPEATDPGHSPGVGH |  |
| CaCH\_scf\_166197\_23\_67 | 1 | 4.83e-08 |  | VKDYTGPGHSPGMGH |  |
| CaAN\_chr\_07\_708661\_67 | 1 | 4.83e-08 |  | VKDYTGPGHSPGMGH |  |
| EuGR\_scf\_8\_418605\_108 | 1 | 5.41e-08 |  | SLGSTSPGHSPSVGH |  |
| EuCA\_ctg\_052267\_53\_108 | 1 | 5.41e-08 |  | SLGSTSPGHSPSVGH |  |
| SpOL\_scf\_21396\_72\_541 | 1 | 6.77e-08 |  | DFGPTSPGGSPGDGH |  |
| SoPI\_ctg\_4939319\_12\_87 | 1 | 1.05e-07 |  | GSEPSGPGHSPGVGH |  |
| SoLY\_chr\_02\_127699\_87 | 1 | 1.05e-07 |  | GSEPSGPGHSPGVGH |  |
| PhHE\_scf\_01000933\_232\_72 | 1 | 1.18e-07 |  | HVDSTTPGHSPSIGH |  |
| HoVU\_ctg\_858320\_43\_100 | 1 | 1.18e-07 |  | VDKETTPGHSPGPGH |  |
| GoRA\_chr\_09\_319933\_80 | 1 | 1.18e-07 |  | DVYPTPRGHSPGAGH |  |
| SoTU\_chr\_02\_104011\_72 | 1 | 1.72e-07 |  | SSDPSGPGHSPGVGH |  |
| PoTR\_chr\_07\_72858\_538 | 1 | 1.72e-07 |  | GFRPAVPIQGPGVGH |  |
| PoTR\_chr\_07\_72674\_462 | 1 | 1.72e-07 |  | GFRPAVPIQGPGVGH |  |
| NiBE\_scf\_126\_2333\_114 | 1 | 1.91e-07 |  | FDGPTGPGHSPGIGH |  |
| NiTO\_scf\_3364\_1326\_203 | 1 | 2.24e-07 |  | GFSRSGPGHSPGIGH |  |
| ZeMA\_chr\_4\_856145\_124 | 1 | 2.37e-07 |  | DSAPTTPGHSPSGGH |  |
| NiSY\_scf\_3607\_327\_74 | 1 | 2.37e-07 |  | DTPTVTPGHSPGMGH |  |
| NiBE\_scf\_1510\_2572\_77 | 1 | 2.37e-07 |  | DTPTVTPGHSPGMGH |  |
| NiTO\_scf\_22023\_508\_79 | 1 | 2.63e-07 |  | DSPTVTPGHSPGMGH |  |
| SoTU\_chr\_08\_156316\_75 | 1 | 2.77e-07 |  | VKDYSGPGHSPGMGH |  |
| SoTU\_chr\_02\_126880\_100 | 1 | 2.77e-07 |  | GFGTSGPGHSPGIGH |  |
| SoPI\_ctg\_5086539\_16\_67 | 1 | 2.77e-07 |  | VKDYSGPGHSPGMGH |  |
| SoLY\_chr\_08\_164998\_78 | 1 | 2.77e-07 |  | VKDYSGPGHSPGMGH |  |
| NiTO\_scf\_9645\_1400\_73 | 1 | 2.77e-07 |  | VKDYSGPGHSPGMGH |  |
| NiTO\_ctg\_18607\_232\_75 | 1 | 2.77e-07 |  | FGGPTGPGHSPGIGH |  |
| NiSY\_scf\_7379\_226\_84 | 1 | 2.77e-07 |  | FGGPTGPGHSPGIGH |  |
| NiSY\_scf\_28833\_905\_71 | 1 | 2.77e-07 |  | VKDYSGPGHSPGMGH |  |
| NiBE\_scf\_58\_1749\_65 | 1 | 2.77e-07 |  | VKDYSGPGHSPGMGH |  |
| NiBE\_scf\_5078\_118\_71 | 1 | 2.77e-07 |  | VKDYSGPGHSPGMGH |  |
| SoTU\_chr\_03\_20601\_107 | 1 | 3.24e-07 |  | GFSPYGRGHSPGIGH |  |
| SoPI\_ctg\_6742575\_3\_114 | 1 | 3.24e-07 |  | GFSPYGRGHSPGIGH |  |
| SoLY\_chr\_03\_22772\_114 | 1 | 3.24e-07 |  | GFSPYGRGHSPGIGH |  |
| PoTR\_chr\_07\_72858\_453 | 1 | 4.43e-07 |  | EHSVTTPGHSPAVGH |  |
| PoTR\_chr\_07\_72674\_377 | 1 | 4.43e-07 |  | EHSVTTPGHSPAVGH |  |
| SoBI\_chr\_07\_234975\_85 | 1 | 9.46e-07 |  | SFPPSTPGHSPSGGH |  |
| NiSY\_scf\_12155\_205\_137 | 1 | 1.55e-06 |  | NVSEGGPGHSPGVGH |  |
| NiBE\_scf\_769\_350\_118 | 1 | 1.55e-06 |  | NVSEGGPGHSPGVGH |  |
| PaVI\_ctg\_02119\_158\_71 | 1 | 1.62e-06 |  | DSATTSPGHSPSGGH |  |
| PaVI\_ctg\_280593\_10\_69 | 1 | 3.48e-06 |  | DSPTTSPGHSPSGGH |  |

Time 2619.4 secs.

Top

##### MEME version

4.9.1 (Release date: Fri Aug 23 16:49:42 2013 +1000)

##### Reference

Timothy L. Bailey and Charles Elkan,
"Fitting a mixture model by expectation maximization to discover motifs in biopolymers",
*Proceedings of the Second International Conference on Intelligent Systems
for Molecular Biology*, pp. 28-36, AAAI Press, Menlo Park, California, 1994.

show training set...

##### Training set:

Datafile = Embryophyta\_domains.fasta  
Alphabet = ACDEFGHIKLMNPQRSTVWY  

|  |  |  |  |  |  |  |  |  |  |  |  |  |  |  |  |  |  |  |  |  |  |  |  |  |  |  |  |  |  |  |  |  |  |  |  |  |  |  |  |  |  |  |  |  |  |  |  |  |  |  |  |  |  |  |  |  |  |  |  |  |  |  |  |  |  |  |  |  |  |  |  |  |  |  |  |  |  |  |  |  |  |  |  |  |  |  |  |  |  |  |  |  |  |  |  |  |  |  |  |  |  |  |  |  |  |  |  |  |  |  |  |  |  |  |  |  |  |  |  |  |  |  |  |  |  |  |  |  |  |  |  |  |  |  |  |  |  |  |  |  |  |  |  |  |  |  |  |  |  |  |  |  |  |  |  |  |  |  |  |  |  |  |  |  |  |  |  |  |  |  |  |  |  |  |  |  |  |  |  |  |  |  |  |  |  |  |  |  |  |  |  |  |  |  |  |  |  |  |  |  |  |  |  |  |  |  |  |  |  |  |  |  |  |  |  |  |  |  |  |  |  |  |  |  |  |  |  |  |  |  |  |  |  |  |  |  |  |  |  |  |  |  |  |  |  |  |  |  |  |  |  |  |  |  |  |  |  |  |  |  |  |  |  |  |  |  |  |  |  |  |  |  |  |  |  |  |  |  |  |  |  |  |  |  |  |  |  |  |  |  |  |  |  |  |  |  |  |  |  |  |  |  |  |  |  |  |  |  |  |  |  |  |  |  |  |  |  |  |  |  |  |  |  |  |  |  |  |  |  |  |  |  |  |  |  |  |  |  |  |  |  |  |  |  |  |  |  |  |  |  |  |  |  |  |  |  |  |  |  |  |  |  |  |  |  |  |  |  |  |  |  |  |  |  |  |  |  |  |  |  |  |  |  |  |  |  |  |  |  |  |  |  |  |  |  |  |  |  |  |  |  |  |  |  |  |  |  |  |  |  |  |  |  |  |  |  |  |  |  |  |  |  |  |  |  |  |  |  |  |  |  |  |  |  |  |  |  |  |  |  |  |  |  |  |  |  |  |  |  |  |  |  |  |  |  |  |  |  |  |  |  |  |  |  |  |  |  |  |  |  |  |  |  |  |  |  |  |  |  |  |  |  |  |  |  |  |  |  |  |  |  |  |  |  |  |  |  |  |  |  |  |  |  |  |  |  |  |  |  |  |  |  |  |  |  |  |  |  |  |  |  |  |  |  |  |  |  |  |  |  |  |  |  |  |  |  |  |  |  |  |  |  |  |  |  |  |  |  |  |  |  |  |  |  |  |  |  |  |  |  |  |  |  |  |  |  |  |  |  |  |  |  |  |  |  |  |  |  |  |  |  |  |  |  |  |  |  |  |  |  |  |  |  |  |  |  |  |  |  |  |  |  |  |  |  |  |  |  |  |  |  |  |  |  |  |  |  |  |  |  |  |  |  |  |  |  |  |  |  |  |  |  |  |  |  |  |  |  |  |  |  |  |  |  |  |  |  |  |  |  |  |  |  |  |  |  |  |  |  |  |  |  |  |  |  |  |  |  |  |  |  |  |  |  |  |  |  |  |  |  |  |  |  |  |  |  |  |  |  |  |  |  |  |  |  |  |  |  |  |  |  |  |  |  |  |  |  |  |  |  |  |  |  |  |  |  |  |  |  |  |  |  |  |  |  |  |  |  |  |  |  |  |  |  |  |  |  |  |  |  |  |  |  |  |  |  |  |  |  |  |  |  |  |  |  |  |  |  |  |  |  |  |  |  |  |  |  |  |  |  |  |  |  |  |  |  |  |  |  |  |  |  |  |  |  |  |  |  |  |  |  |  |  |  |  |  |  |  |  |  |  |  |  |  |  |  |  |  |  |  |  |  |  |  |  |  |  |  |  |  |  |  |  |  |  |  |  |  |  |  |  |  |  |  |  |  |  |  |  |  |  |  |  |  |  |  |  |  |  |  |  |  |  |  |  |  |  |  |  |  |  |  |  |  |  |  |  |  |  |  |  |  |  |  |  |  |  |  |  |  |  |  |  |  |  |  |  |  |  |  |  |  |  |  |  |  |  |  |  |  |  |  |  |  |  |  |  |  |  |  |  |  |  |  |  |  |  |  |  |  |  |  |  |  |  |  |  |  |  |  |  |  |  |  |  |  |  |  |  |  |  |  |  |  |  |  |  |  |  |  |  |  |  |  |  |  |  |  |  |  |  |  |  |  |  |  |  |  |  |  |  |  |  |  |  |  |  |  |  |  |  |  |  |  |  |  |  |  |  |  |  |  |  |  |  |  |  |  |  |  |  |  |  |  |  |  |  |  |  |  |  |  |  |  |  |  |  |  |  |  |  |  |  |  |  |  |  |  |  |  |  |  |  |  |  |  |  |  |  |  |  |  |  |  |  |  |  |  |  |  |  |  |  |  |  |  |  |  |  |  |  |  |  |  |  |  |  |  |  |  |  |  |  |  |  |  |  |  |  |  |  |  |  |  |  |  |  |  |  |  |  |  |  |  |  |  |  |  |  |  |  |  |  |  |  |  |  |  |  |  |  |  |  |  |  |  |  |  |  |  |  |  |  |  |  |  |  |  |  |  |  |  |  |  |  |  |  |  |  |  |  |  |  |  |  |  |  |  |  |  |  |  |  |  |  |  |  |  |  |  |  |  |  |  |  |  |  |  |  |  |  |  |  |  |  |  |  |  |  |  |  |  |  |  |  |  |  |  |  |  |  |  |  |  |  |  |  |  |  |  |  |  |  |  |  |  |  |  |  |  |  |  |  |  |  |  |  |  |  |  |  |  |  |  |  |  |  |  |  |  |  |  |  |  |  |  |  |  |  |  |  |  |  |  |  |  |  |  |  |  |  |  |  |  |  |  |  |  |  |  |  |  |  |  |  |  |  |  |  |  |  |  |  |  |  |  |  |  |  |  |  |  |  |  |  |  |  |  |  |  |  |  |  |  |  |  |  |  |  |  |  |  |  |  |  |  |  |  |  |  |  |  |  |  |  |  |  |  |  |  |  |  |  |  |  |  |  |  |  |  |  |  |  |  |  |  |  |  |  |  |  |  |  |  |  |  |  |  |  |  |  |  |  |  |  |  |  |  |  |  |  |  |  |  |  |  |  |  |  |  |  |  |  |  |  |  |  |  |  |  |  |  |  |  |  |  |  |  |  |  |  |  |  |  |  |  |  |  |  |  |  |  |  |  |  |  |  |  |  |  |  |  |  |  |  |  |  |  |  |  |  |  |  |  |  |  |  |  |  |  |  |  |  |  |  |  |  |  |  |  |  |  |  |  |  |  |  |  |  |  |  |  |  |  |  |  |  |  |  |  |  |  |  |  |  |  |  |  |  |  |  |  |  |  |  |  |  |  |  |  |  |  |  |  |  |  |  |  |  |  |  |  |  |  |  |  |  |  |  |  |  |  |  |  |  |  |  |  |  |  |  |  |  |  |  |  |  |  |  |  |  |  |  |  |  |  |  |  |  |  |  |  |  |  |  |  |  |  |  |  |  |  |  |  |  |  |  |  |  |  |  |  |  |  |  |  |  |  |  |  |  |  |  |  |  |  |  |  |  |  |  |  |  |  |  |  |  |  |  |  |  |  |  |  |  |  |  |  |  |  |  |  |  |  |  |  |  |  |  |  |  |  |  |  |  |  |  |  |  |  |  |  |  |  |  |  |  |  |  |  |  |  |  |  |  |  |  |  |  |  |  |  |  |  |  |  |  |  |  |  |  |  |  |  |  |  |  |  |  |  |  |  |  |  |  |  |  |  |  |  |  |  |  |  |  |  |  |  |  |  |  |  |  |  |  |  |  |  |  |  |  |  |  |  |  |  |  |  |  |  |  |  |  |  |  |  |  |  |  |  |  |  |  |  |  |  |  |  |  |  |  |  |  |  |  |  |  |  |  |  |  |  |  |  |  |  |  |  |  |  |  |  |  |  |  |  |  |  |  |  |  |  |  |  |  |  |  |  |  |  |  |  |  |  |  |  |  |  |  |  |  |  |  |  |  |  |  |  |  |  |  |  |  |  |  |  |  |  |  |  |  |  |  |  |  |  |  |  |  |  |  |  |  |  |  |  |  |  |  |  |  |  |  |  |  |  |  |  |  |  |  |  |  |  |  |  |  |  |  |  |  |  |  |  |  |  |  |  |  |  |  |  |  |  |  |  |  |  |  |  |  |  |  |  |  |  |  |  |  |  |  |  |  |  |  |  |  |  |  |  |  |  |  |  |  |  |  |  |  |  |  |  |  |  |  |  |  |  |  |  |  |  |  |  |  |  |  |  |  |  |  |  |  |  |  |  |  |  |  |  |  |  |  |  |  |  |  |  |  |  |  |  |  |  |  |  |  |  |  |  |  |  |  |  |  |  |  |  |  |  |  |  |  |  |  |  |  |  |  |  |  |  |  |  |  |  |  |  |  |  |  |  |  |  |  |  |  |  |  |  |  |  |  |  |  |  |  |  |  |  |  |  |  |  |  |  |  |  |  |  |  |  |  |  |  |  |  |  |  |  |  |  |  |  |  |  |  |  |  |  |  |  |  |  |  |  |  |  |  |  |  |  |  |  |  |  |  |  |  |  |  |  |  |  |  |  |  |  |  |  |  |  |  |  |  |  |  |  |  |  |  |  |  |  |  |  |  |  |  |  |  |  |  |  |  |  |  |  |  |  |  |  |  |  |  |  |  |  |  |  |  |  |  |  |  |  |  |  |  |  |  |  |  |  |  |  |  |  |  |  |  |  |  |  |  |  |  |  |  |  |  |  |  |  |  |  |  |  |  |  |  |  |  |  |  |  |  |  |  |  |  |  |  |  |  |  |  |  |  |  |  |  |  |  |  |  |  |  |  |  |  |  |  |  |  |  |  |  |  |  |  |  |  |  |  |  |  |  |  |  |  |  |  |  |  |  |  |  |  |  |  |  |  |  |  |  |  |  |  |  |  |  |  |  |  |  |  |  |  |  |  |  |  |  |  |  |  |  |  |  |  |  |  |  |  |  |  |  |  |  |  |  |  |  |  |  |  |  |  |  |  |  |  |  |  |  |  |  |  |  |  |  |  |  |  |  |  |  |  |  |  |  |  |  |  |  |  |  |  |  |  |  |  |  |  |  |  |  |  |  |  |  |  |  |  |  |  |  |  |  |  |  |  |  |  |  |  |  |  |  |  |  |  |  |  |  |  |  |  |  |  |  |  |  |  |  |  |  |  |  |  |  |  |  |  |  |  |  |  |  |  |  |  |  |  |  |  |  |  |  |  |  |  |  |  |  |  |  |  |  |  |  |  |  |  |  |  |  |  |  |  |  |  |  |  |  |  |  |  |  |  |  |  |  |  |  |  |  |  |  |  |  |  |  |  |  |  |  |  |  |  |  |  |  |  |  |  |  |  |  |  |  |  |  |  |  |  |  |  |  |  |  |  |  |  |  |  |  |  |  |  |  |  |  |  |  |  |  |  |  |  |  |  |  |  |  |  |  |  |  |  |  |  |  |  |  |  |  |  |  |  |  |  |  |  |  |  |  |  |  |  |  |  |  |  |  |  |  |  |  |  |  |  |  |  |  |  |  |  |  |  |  |  |  |  |  |  |  |  |  |  |  |  |  |  |  |  |  |  |  |  |  |  |  |  |  |  |  |  |  |  |  |  |  |  |  |  |  |  |  |  |  |  |  |  |  |  |  |  |  |  |  |  |  |  |  |  |  |  |  |  |  |  |  |  |  |  |  |  |  |  |  |  |  |  |  |  |  |  |  |  |  |  |  |  |  |  |  |  |  |  |  |  |  |  |  |  |  |  |  |  |  |  |  |  |  |  |  |  |  |  |  |  |  |  |  |  |  |  |  |  |  |  |  |  |  |  |  |  |  |  |  |  |  |  |  |  |  |  |  |  |  |  |  |  |  |  |  |  |  |  |  |  |  |  |  |  |  |  |  |  |  |  |  |  |  |  |  |  |  |  |  |  |  |  |  |  |  |  |  |  |  |  |  |  |  |  |  |  |  |  |  |  |  |  |  |  |  |  |  |  |  |  |  |  |  |  |  |  |  |  |  |  |  |  |  |  |  |  |  |  |  |  |  |  |  |  |  |  |  |  |  |  |  |  |  |  |  |  |  |  |  |  |  |  |  |  |  |  |  |  |  |  |  |  |  |  |  |  |  |  |  |  |  |  |  |  |  |  |  |  |  |  |  |  |  |  |  |  |  |  |  |  |  |  |  |  |  |  |  |  |  |  |  |  |  |  |  |  |  |  |  |  |  |  |  |  |  |  |  |  |  |  |  |  |  |  |  |  |  |  |  |  |  |  |  |  |  |  |  |  |  |  |  |  |  |  |  |  |  |  |  |  |  |  |  |  |  |  |  |  |  |  |  |  |  |  |  |  |  |  |  |  |  |  |  |  |  |  |  |  |  |  |  |  |  |  |  |  |  |  |  |  |  |  |  |  |  |  |  |  |  |  |  |  |  |  |  |  |  |  |  |  |  |  |  |  |  |  |  |  |  |  |  |  |  |  |  |  |  |  |  |  |  |  |  |  |  |  |  |  |  |  |  |  |  |  |  |  |  |  |  |  |  |  |  |  |  |  |  |  |  |  |  |  |  |  |  |  |  |  |  |  |  |  |  |  |  |  |  |  |  |  |  |  |  |  |  |  |  |  |  |  |  |  |  |  |  |  |  |  |  |  |  |  |  |  |  |  |  |  |  |  |  |  |  |  |  |  |  |  |  |  |  |  |  |  |  |  |  |  |  |  |  |  |  |  |  |  |  |  |  |  |  |  |  |  |  |  |  |  |  |  |  |  |  |  |  |  |  |  |  |  |  |  |  |  |  |  |  |  |  |  |  |  |  |  |  |  |  |  |  |  |  |  |  |  |  |  |  |  |  |  |  |  |  |  |  |  |  |  |  |  |  |  |  |  |  |  |  |  |  |  |  |  |  |  |  |  |  |  |  |  |  |  |  |  |  |  |  |  |  |  |  |  |  |  |  |  |  |  |  |  |  |  |  |  |  |  |  |  |  |  |  |  |  |  |  |  |  |  |  |  |  |  |  |  |  |  |  |  |  |  |  |  |  |  |  |  |  |  |  |  |  |  |  |  |  |  |  |  |  |  |  |  |  |  |  |  |  |  |  |  |  |  |  |  |  |  |  |  |  |  |  |  |  |  |  |  |  |  |  |  |  |  |  |  |  |  |  |  |  |  |  |  |  |  |  |  |  |  |  |  |  |  |  |  |  |  |  |  |  |  |  |  |  |  |  |  |  |  |  |  |  |  |  |  |  |  |  |  |  |  |  |  |  |  |  |  |  |  |  |  |  |  |  |  |  |  |  |  |  |  |  |  |  |  |  |  |  |  |  |  |  |  |  |  |  |  |  |  |  |  |  |  |  |  |  |  |  |  |  |  |  |  |  |  |  |  |  |  |  |  |  |  |  |  |  |  |  |  |  |  |  |  |  |  |  |  |  |  |  |  |  |  |  |  |  |  |  |  |  |  |  |  |  |  |  |  |  |  |  |  |  |  |  |  |  |  |  |  |  |  |  |  |  |  |  |  |  |  |  |  |  |  |  |  |  |  |  |  |  |  |  |  |  |  |  |  |  |  |  |  |  |  |  |  |  |  |  |  |  |  |  |  |  |  |  |  |  |  |  |  |  |  |  |  |  |  |  |  |  |  |  |  |  |  |  |  |  |  |  |  |  |  |  |  |  |  |  |  |  |  |  |  |  |  |  |  |  |  |  |  |  |  |  |  |  |  |  |  |  |  |  |  |  |  |  |  |  |  |  |  |  |  |  |  |  |  |  |  |  |  |  |  |  |  |  |  |  |  |  |  |  |  |  |  |  |  |  |  |  |  |  |  |  |  |  |  |  |  |  |  |  |  |  |  |  |  |  |  |  |  |  |  |  |  |  |  |  |  |  |  |  |  |  |  |  |  |  |  |  |  |  |  |  |  |  |  |  |  |  |  |  |  |  |  |  |  |  |  |  |  |  |  |  |  |  |  |  |  |  |  |  |  |  |  |  |  |  |  |  |  |  |  |  |  |  |  |  |  |  |  |  |  |  |  |  |  |  |  |  |  |  |  |  |  |  |
| --- | --- | --- | --- | --- | --- | --- | --- | --- | --- | --- | --- | --- | --- | --- | --- | --- | --- | --- | --- | --- | --- | --- | --- | --- | --- | --- | --- | --- | --- | --- | --- | --- | --- | --- | --- | --- | --- | --- | --- | --- | --- | --- | --- | --- | --- | --- | --- | --- | --- | --- | --- | --- | --- | --- | --- | --- | --- | --- | --- | --- | --- | --- | --- | --- | --- | --- | --- | --- | --- | --- | --- | --- | --- | --- | --- | --- | --- | --- | --- | --- | --- | --- | --- | --- | --- | --- | --- | --- | --- | --- | --- | --- | --- | --- | --- | --- | --- | --- | --- | --- | --- | --- | --- | --- | --- | --- | --- | --- | --- | --- | --- | --- | --- | --- | --- | --- | --- | --- | --- | --- | --- | --- | --- | --- | --- | --- | --- | --- | --- | --- | --- | --- | --- | --- | --- | --- | --- | --- | --- | --- | --- | --- | --- | --- | --- | --- | --- | --- | --- | --- | --- | --- | --- | --- | --- | --- | --- | --- | --- | --- | --- | --- | --- | --- | --- | --- | --- | --- | --- | --- | --- | --- | --- | --- | --- | --- | --- | --- | --- | --- | --- | --- | --- | --- | --- | --- | --- | --- | --- | --- | --- | --- | --- | --- | --- | --- | --- | --- | --- | --- | --- | --- | --- | --- | --- | --- | --- | --- | --- | --- | --- | --- | --- | --- | --- | --- | --- | --- | --- | --- | --- | --- | --- | --- | --- | --- | --- | --- | --- | --- | --- | --- | --- | --- | --- | --- | --- | --- | --- | --- | --- | --- | --- | --- | --- | --- | --- | --- | --- | --- | --- | --- | --- | --- | --- | --- | --- | --- | --- | --- | --- | --- | --- | --- | --- | --- | --- | --- | --- | --- | --- | --- | --- | --- | --- | --- | --- | --- | --- | --- | --- | --- | --- | --- | --- | --- | --- | --- | --- | --- | --- | --- | --- | --- | --- | --- | --- | --- | --- | --- | --- | --- | --- | --- | --- | --- | --- | --- | --- | --- | --- | --- | --- | --- | --- | --- | --- | --- | --- | --- | --- | --- | --- | --- | --- | --- | --- | --- | --- | --- | --- | --- | --- | --- | --- | --- | --- | --- | --- | --- | --- | --- | --- | --- | --- | --- | --- | --- | --- | --- | --- | --- | --- | --- | --- | --- | --- | --- | --- | --- | --- | --- | --- | --- | --- | --- | --- | --- | --- | --- | --- | --- | --- | --- | --- | --- | --- | --- | --- | --- | --- | --- | --- | --- | --- | --- | --- | --- | --- | --- | --- | --- | --- | --- | --- | --- | --- | --- | --- | --- | --- | --- | --- | --- | --- | --- | --- | --- | --- | --- | --- | --- | --- | --- | --- | --- | --- | --- | --- | --- | --- | --- | --- | --- | --- | --- | --- | --- | --- | --- | --- | --- | --- | --- | --- | --- | --- | --- | --- | --- | --- | --- | --- | --- | --- | --- | --- | --- | --- | --- | --- | --- | --- | --- | --- | --- | --- | --- | --- | --- | --- | --- | --- | --- | --- | --- | --- | --- | --- | --- | --- | --- | --- | --- | --- | --- | --- | --- | --- | --- | --- | --- | --- | --- | --- | --- | --- | --- | --- | --- | --- | --- | --- | --- | --- | --- | --- | --- | --- | --- | --- | --- | --- | --- | --- | --- | --- | --- | --- | --- | --- | --- | --- | --- | --- | --- | --- | --- | --- | --- | --- | --- | --- | --- | --- | --- | --- | --- | --- | --- | --- | --- | --- | --- | --- | --- | --- | --- | --- | --- | --- | --- | --- | --- | --- | --- | --- | --- | --- | --- | --- | --- | --- | --- | --- | --- | --- | --- | --- | --- | --- | --- | --- | --- | --- | --- | --- | --- | --- | --- | --- | --- | --- | --- | --- | --- | --- | --- | --- | --- | --- | --- | --- | --- | --- | --- | --- | --- | --- | --- | --- | --- | --- | --- | --- | --- | --- | --- | --- | --- | --- | --- | --- | --- | --- | --- | --- | --- | --- | --- | --- | --- | --- | --- | --- | --- | --- | --- | --- | --- | --- | --- | --- | --- | --- | --- | --- | --- | --- | --- | --- | --- | --- | --- | --- | --- | --- | --- | --- | --- | --- | --- | --- | --- | --- | --- | --- | --- | --- | --- | --- | --- | --- | --- | --- | --- | --- | --- | --- | --- | --- | --- | --- | --- | --- | --- | --- | --- | --- | --- | --- | --- | --- | --- | --- | --- | --- | --- | --- | --- | --- | --- | --- | --- | --- | --- | --- | --- | --- | --- | --- | --- | --- | --- | --- | --- | --- | --- | --- | --- | --- | --- | --- | --- | --- | --- | --- | --- | --- | --- | --- | --- | --- | --- | --- | --- | --- | --- | --- | --- | --- | --- | --- | --- | --- | --- | --- | --- | --- | --- | --- | --- | --- | --- | --- | --- | --- | --- | --- | --- | --- | --- | --- | --- | --- | --- | --- | --- | --- | --- | --- | --- | --- | --- | --- | --- | --- | --- | --- | --- | --- | --- | --- | --- | --- | --- | --- | --- | --- | --- | --- | --- | --- | --- | --- | --- | --- | --- | --- | --- | --- | --- | --- | --- | --- | --- | --- | --- | --- | --- | --- | --- | --- | --- | --- | --- | --- | --- | --- | --- | --- | --- | --- | --- | --- | --- | --- | --- | --- | --- | --- | --- | --- | --- | --- | --- | --- | --- | --- | --- | --- | --- | --- | --- | --- | --- | --- | --- | --- | --- | --- | --- | --- | --- | --- | --- | --- | --- | --- | --- | --- | --- | --- | --- | --- | --- | --- | --- | --- | --- | --- | --- | --- | --- | --- | --- | --- | --- | --- | --- | --- | --- | --- | --- | --- | --- | --- | --- | --- | --- | --- | --- | --- | --- | --- | --- | --- | --- | --- | --- | --- | --- | --- | --- | --- | --- | --- | --- | --- | --- | --- | --- | --- | --- | --- | --- | --- | --- | --- | --- | --- | --- | --- | --- | --- | --- | --- | --- | --- | --- | --- | --- | --- | --- | --- | --- | --- | --- | --- | --- | --- | --- | --- | --- | --- | --- | --- | --- | --- | --- | --- | --- | --- | --- | --- | --- | --- | --- | --- | --- | --- | --- | --- | --- | --- | --- | --- | --- | --- | --- | --- | --- | --- | --- | --- | --- | --- | --- | --- | --- | --- | --- | --- | --- | --- | --- | --- | --- | --- | --- | --- | --- | --- | --- | --- | --- | --- | --- | --- | --- | --- | --- | --- | --- | --- | --- | --- | --- | --- | --- | --- | --- | --- | --- | --- | --- | --- | --- | --- | --- | --- | --- | --- | --- | --- | --- | --- | --- | --- | --- | --- | --- | --- | --- | --- | --- | --- | --- | --- | --- | --- | --- | --- | --- | --- | --- | --- | --- | --- | --- | --- | --- | --- | --- | --- | --- | --- | --- | --- | --- | --- | --- | --- | --- | --- | --- | --- | --- | --- | --- | --- | --- | --- | --- | --- | --- | --- | --- | --- | --- | --- | --- | --- | --- | --- | --- | --- | --- | --- | --- | --- | --- | --- | --- | --- | --- | --- | --- | --- | --- | --- | --- | --- | --- | --- | --- | --- | --- | --- | --- | --- | --- | --- | --- | --- | --- | --- | --- | --- | --- | --- | --- | --- | --- | --- | --- | --- | --- | --- | --- | --- | --- | --- | --- | --- | --- | --- | --- | --- | --- | --- | --- | --- | --- | --- | --- | --- | --- | --- | --- | --- | --- | --- | --- | --- | --- | --- | --- | --- | --- | --- | --- | --- | --- | --- | --- | --- | --- | --- | --- | --- | --- | --- | --- | --- | --- | --- | --- | --- | --- | --- | --- | --- | --- | --- | --- | --- | --- | --- | --- | --- | --- | --- | --- | --- | --- | --- | --- | --- | --- | --- | --- | --- | --- | --- | --- | --- | --- | --- | --- | --- | --- | --- | --- | --- | --- | --- | --- | --- | --- | --- | --- | --- | --- | --- | --- | --- | --- | --- | --- | --- | --- | --- | --- | --- | --- | --- | --- | --- | --- | --- | --- | --- | --- | --- | --- | --- | --- | --- | --- | --- | --- | --- | --- | --- | --- | --- | --- | --- | --- | --- | --- | --- | --- | --- | --- | --- | --- | --- | --- | --- | --- | --- | --- | --- | --- | --- | --- | --- | --- | --- | --- | --- | --- | --- | --- | --- | --- | --- | --- | --- | --- | --- | --- | --- | --- | --- | --- | --- | --- | --- | --- | --- | --- | --- | --- | --- | --- | --- | --- | --- | --- | --- | --- | --- | --- | --- | --- | --- | --- | --- | --- | --- | --- | --- | --- | --- | --- | --- | --- | --- | --- | --- | --- | --- | --- | --- | --- | --- | --- | --- | --- | --- | --- | --- | --- | --- | --- | --- | --- | --- | --- | --- | --- | --- | --- | --- | --- | --- | --- | --- | --- | --- | --- | --- | --- | --- | --- | --- | --- | --- | --- | --- | --- | --- | --- | --- | --- | --- | --- | --- | --- | --- | --- | --- | --- | --- | --- | --- | --- | --- | --- | --- | --- | --- | --- | --- | --- | --- | --- | --- | --- | --- | --- | --- | --- | --- | --- | --- | --- | --- | --- | --- | --- | --- | --- | --- | --- | --- | --- | --- | --- | --- | --- | --- | --- | --- | --- | --- | --- | --- | --- | --- | --- | --- | --- | --- | --- | --- | --- | --- | --- | --- | --- | --- | --- | --- | --- | --- | --- | --- | --- | --- | --- | --- | --- | --- | --- | --- | --- | --- | --- | --- | --- | --- | --- | --- | --- | --- | --- | --- | --- | --- | --- | --- | --- | --- | --- | --- | --- | --- | --- | --- | --- | --- | --- | --- | --- | --- | --- | --- | --- | --- | --- | --- | --- | --- | --- | --- | --- | --- | --- | --- | --- | --- | --- | --- | --- | --- | --- | --- | --- | --- | --- | --- | --- | --- | --- | --- | --- | --- | --- | --- | --- | --- | --- | --- | --- | --- | --- | --- | --- | --- | --- | --- | --- | --- | --- | --- | --- | --- | --- | --- | --- | --- | --- | --- | --- | --- | --- | --- | --- | --- | --- | --- | --- | --- | --- | --- | --- | --- | --- | --- | --- | --- | --- | --- | --- | --- | --- | --- | --- | --- | --- | --- | --- | --- | --- | --- | --- | --- | --- | --- | --- | --- | --- | --- | --- | --- | --- | --- | --- | --- | --- | --- | --- | --- | --- | --- | --- | --- | --- | --- | --- | --- | --- | --- | --- | --- | --- | --- | --- | --- | --- | --- | --- | --- | --- | --- | --- | --- | --- | --- | --- | --- | --- | --- | --- | --- | --- | --- | --- | --- | --- | --- | --- | --- | --- | --- | --- | --- | --- | --- | --- | --- | --- | --- | --- | --- | --- | --- | --- | --- | --- | --- | --- | --- | --- | --- | --- | --- | --- | --- | --- | --- | --- | --- | --- | --- | --- | --- | --- | --- | --- | --- | --- | --- | --- | --- | --- | --- | --- | --- | --- | --- | --- | --- | --- | --- | --- | --- | --- | --- | --- | --- | --- | --- | --- | --- | --- | --- | --- | --- | --- | --- | --- | --- | --- | --- | --- | --- | --- | --- | --- | --- | --- | --- | --- | --- | --- | --- | --- | --- | --- | --- | --- | --- | --- | --- | --- | --- | --- | --- | --- | --- | --- | --- | --- | --- | --- | --- | --- | --- | --- | --- | --- | --- | --- | --- | --- | --- | --- | --- | --- | --- | --- | --- | --- | --- | --- | --- | --- | --- | --- | --- | --- | --- | --- | --- | --- | --- | --- | --- | --- | --- | --- | --- | --- | --- | --- | --- | --- | --- | --- | --- | --- | --- | --- | --- | --- | --- | --- | --- | --- | --- | --- | --- | --- | --- | --- | --- | --- | --- | --- | --- | --- | --- | --- | --- | --- | --- | --- | --- | --- | --- | --- | --- | --- | --- | --- | --- | --- | --- | --- | --- | --- | --- | --- | --- | --- | --- | --- | --- | --- | --- | --- | --- | --- | --- | --- | --- | --- | --- | --- | --- | --- | --- | --- | --- | --- | --- | --- | --- | --- | --- | --- | --- | --- | --- | --- | --- | --- | --- | --- | --- | --- | --- | --- | --- | --- | --- | --- | --- | --- | --- | --- | --- | --- | --- | --- | --- | --- | --- | --- | --- | --- | --- | --- | --- | --- | --- | --- | --- | --- | --- | --- | --- | --- | --- | --- | --- | --- | --- | --- | --- | --- | --- | --- | --- | --- | --- | --- | --- | --- | --- | --- | --- | --- | --- | --- | --- | --- | --- | --- | --- | --- | --- | --- | --- | --- | --- | --- | --- | --- | --- | --- | --- | --- | --- | --- | --- | --- | --- | --- | --- | --- | --- | --- | --- | --- | --- | --- | --- | --- | --- | --- | --- | --- | --- | --- | --- | --- | --- | --- | --- | --- | --- | --- | --- | --- | --- | --- | --- | --- | --- | --- | --- | --- | --- | --- | --- | --- | --- | --- | --- | --- | --- | --- | --- | --- | --- | --- | --- | --- | --- | --- | --- | --- | --- | --- | --- | --- | --- | --- | --- | --- | --- | --- | --- | --- | --- | --- | --- | --- | --- | --- | --- | --- | --- | --- | --- | --- | --- | --- | --- | --- | --- | --- | --- | --- | --- | --- | --- | --- | --- | --- | --- | --- | --- | --- | --- | --- | --- | --- | --- | --- | --- | --- | --- | --- | --- | --- | --- | --- | --- | --- | --- | --- | --- | --- | --- | --- | --- | --- | --- | --- | --- | --- | --- | --- | --- | --- | --- | --- | --- | --- | --- | --- | --- | --- | --- | --- | --- | --- | --- | --- | --- | --- | --- | --- | --- | --- | --- | --- | --- | --- | --- | --- | --- | --- | --- | --- | --- | --- | --- | --- | --- | --- | --- | --- | --- | --- | --- | --- | --- | --- | --- | --- | --- | --- | --- | --- | --- | --- | --- | --- | --- | --- | --- | --- | --- | --- | --- | --- | --- | --- | --- | --- | --- | --- | --- | --- | --- | --- | --- | --- | --- | --- | --- | --- | --- | --- | --- | --- | --- | --- | --- | --- | --- | --- | --- | --- | --- | --- | --- | --- | --- | --- | --- | --- | --- | --- | --- | --- | --- | --- | --- | --- | --- | --- | --- | --- | --- | --- | --- | --- | --- | --- | --- | --- | --- | --- | --- | --- | --- | --- | --- | --- | --- | --- | --- | --- | --- | --- | --- | --- | --- | --- | --- | --- | --- | --- | --- | --- | --- | --- | --- | --- | --- | --- | --- | --- | --- | --- | --- | --- | --- | --- | --- | --- | --- | --- | --- | --- | --- | --- | --- | --- | --- | --- | --- | --- | --- | --- | --- | --- | --- | --- | --- | --- | --- | --- | --- | --- | --- | --- | --- | --- | --- | --- | --- | --- | --- | --- | --- | --- | --- | --- | --- | --- | --- | --- | --- | --- | --- | --- | --- | --- | --- | --- | --- | --- | --- | --- | --- | --- | --- | --- | --- | --- | --- | --- | --- | --- | --- | --- | --- | --- | --- | --- | --- | --- | --- | --- | --- | --- | --- | --- | --- | --- | --- | --- | --- | --- | --- | --- | --- | --- | --- | --- | --- | --- | --- | --- | --- | --- | --- | --- | --- | --- | --- | --- | --- | --- | --- | --- | --- | --- | --- | --- | --- | --- | --- | --- | --- | --- | --- | --- | --- | --- | --- | --- | --- | --- | --- | --- | --- | --- | --- | --- | --- | --- | --- | --- | --- | --- | --- | --- | --- | --- | --- | --- | --- | --- | --- | --- | --- | --- | --- | --- | --- | --- | --- | --- | --- | --- | --- | --- | --- | --- | --- | --- | --- | --- | --- | --- | --- | --- | --- | --- | --- | --- | --- | --- | --- | --- | --- | --- | --- | --- | --- | --- | --- | --- | --- | --- | --- | --- | --- | --- | --- | --- | --- | --- | --- | --- | --- | --- | --- | --- | --- | --- | --- | --- | --- | --- | --- | --- | --- | --- | --- | --- | --- | --- | --- | --- | --- | --- | --- | --- | --- | --- | --- | --- | --- | --- | --- | --- | --- | --- | --- | --- | --- | --- | --- | --- | --- | --- | --- | --- | --- | --- | --- | --- | --- | --- | --- | --- | --- | --- | --- | --- | --- | --- | --- | --- | --- | --- | --- | --- | --- | --- | --- | --- | --- | --- | --- | --- | --- | --- | --- | --- | --- | --- | --- | --- | --- | --- | --- | --- | --- | --- | --- | --- | --- | --- | --- | --- | --- | --- | --- | --- | --- | --- | --- | --- | --- | --- | --- | --- | --- | --- | --- | --- | --- | --- | --- | --- | --- | --- | --- | --- | --- | --- | --- | --- | --- | --- | --- | --- | --- | --- | --- | --- | --- | --- | --- | --- | --- | --- | --- | --- | --- | --- | --- | --- | --- | --- | --- | --- | --- | --- | --- | --- | --- | --- | --- | --- | --- | --- | --- | --- | --- | --- | --- | --- | --- | --- | --- | --- | --- | --- | --- | --- | --- | --- | --- | --- | --- | --- | --- | --- | --- | --- | --- | --- | --- | --- | --- | --- | --- | --- | --- | --- | --- | --- | --- | --- | --- | --- | --- | --- | --- | --- | --- | --- | --- | --- | --- | --- | --- | --- | --- | --- | --- | --- | --- | --- | --- | --- | --- | --- | --- | --- | --- | --- | --- | --- | --- | --- | --- | --- | --- | --- | --- | --- | --- | --- | --- | --- | --- | --- | --- | --- | --- | --- | --- | --- | --- | --- | --- | --- | --- | --- | --- | --- | --- | --- | --- | --- | --- | --- | --- | --- | --- | --- | --- | --- | --- | --- | --- | --- | --- | --- | --- | --- | --- | --- | --- | --- | --- | --- | --- | --- | --- | --- | --- | --- | --- | --- | --- | --- | --- | --- | --- | --- | --- | --- | --- | --- | --- | --- | --- | --- | --- | --- | --- | --- | --- | --- | --- | --- | --- | --- | --- | --- | --- | --- | --- | --- | --- | --- | --- | --- | --- | --- | --- | --- | --- | --- | --- | --- | --- | --- | --- | --- | --- | --- | --- | --- | --- | --- | --- | --- | --- | --- | --- | --- | --- | --- | --- | --- | --- | --- | --- | --- | --- | --- | --- | --- | --- | --- | --- | --- | --- | --- | --- | --- | --- | --- | --- | --- | --- | --- | --- | --- | --- | --- | --- | --- | --- | --- | --- | --- | --- | --- | --- | --- | --- | --- | --- | --- | --- | --- | --- | --- | --- | --- | --- | --- | --- | --- | --- | --- | --- | --- | --- | --- | --- | --- | --- | --- | --- | --- | --- | --- | --- | --- | --- | --- | --- | --- | --- | --- | --- | --- | --- | --- | --- | --- | --- | --- | --- | --- | --- | --- | --- | --- | --- | --- | --- | --- | --- | --- | --- | --- | --- | --- | --- | --- | --- | --- | --- | --- | --- | --- | --- | --- | --- | --- | --- | --- | --- | --- | --- | --- | --- | --- | --- | --- | --- | --- | --- | --- | --- | --- | --- | --- | --- | --- | --- | --- | --- | --- | --- | --- | --- | --- | --- | --- | --- | --- | --- | --- | --- | --- | --- | --- | --- | --- | --- | --- | --- | --- | --- | --- | --- | --- | --- | --- | --- | --- | --- | --- | --- | --- | --- | --- | --- | --- | --- | --- | --- | --- | --- | --- | --- | --- | --- | --- | --- | --- | --- | --- | --- | --- | --- | --- | --- | --- | --- | --- | --- | --- | --- | --- | --- | --- | --- | --- | --- | --- | --- | --- | --- | --- | --- | --- | --- | --- | --- | --- | --- | --- | --- | --- | --- | --- | --- | --- | --- | --- | --- | --- | --- | --- | --- | --- | --- | --- | --- | --- | --- | --- | --- | --- | --- | --- | --- | --- | --- | --- | --- | --- | --- | --- | --- | --- | --- | --- | --- | --- | --- | --- | --- | --- | --- | --- | --- | --- | --- | --- | --- | --- | --- | --- | --- | --- | --- | --- | --- | --- | --- | --- | --- | --- | --- | --- | --- | --- | --- | --- | --- | --- | --- | --- | --- | --- | --- | --- | --- | --- | --- | --- | --- | --- | --- | --- | --- | --- | --- | --- | --- | --- | --- | --- | --- | --- | --- | --- | --- | --- | --- | --- | --- | --- | --- | --- | --- | --- | --- | --- | --- | --- | --- | --- | --- | --- | --- | --- | --- | --- | --- | --- | --- | --- | --- | --- | --- | --- | --- | --- | --- | --- | --- | --- | --- | --- | --- | --- | --- | --- | --- | --- | --- | --- | --- | --- | --- | --- | --- | --- | --- | --- | --- | --- | --- | --- | --- | --- | --- | --- | --- | --- | --- | --- | --- | --- | --- | --- | --- | --- | --- | --- | --- | --- | --- | --- | --- | --- | --- | --- | --- | --- | --- | --- | --- | --- | --- | --- | --- | --- | --- | --- | --- | --- | --- | --- | --- | --- | --- | --- | --- | --- | --- | --- | --- | --- | --- | --- | --- | --- | --- | --- | --- | --- | --- | --- | --- | --- | --- | --- | --- | --- | --- | --- | --- | --- | --- | --- | --- | --- | --- | --- | --- | --- | --- | --- | --- | --- | --- | --- | --- | --- | --- | --- | --- | --- | --- | --- | --- | --- | --- | --- | --- | --- | --- | --- | --- | --- | --- | --- | --- | --- | --- | --- | --- | --- | --- | --- | --- | --- | --- | --- | --- | --- | --- | --- | --- | --- | --- | --- | --- | --- | --- | --- | --- | --- | --- | --- | --- | --- | --- | --- | --- | --- | --- | --- | --- | --- | --- | --- | --- | --- | --- | --- | --- | --- | --- | --- | --- | --- | --- | --- | --- | --- | --- | --- | --- | --- | --- | --- | --- | --- | --- | --- | --- | --- | --- | --- | --- | --- | --- | --- | --- | --- | --- | --- | --- | --- | --- | --- | --- | --- | --- | --- | --- | --- | --- | --- | --- | --- | --- | --- | --- | --- | --- | --- | --- | --- | --- | --- | --- | --- | --- | --- | --- | --- | --- | --- | --- | --- | --- | --- | --- | --- | --- | --- | --- | --- | --- | --- | --- | --- | --- | --- | --- | --- | --- | --- | --- | --- | --- | --- | --- | --- | --- | --- | --- | --- | --- | --- | --- | --- | --- | --- | --- | --- | --- | --- | --- | --- | --- | --- | --- | --- | --- | --- | --- | --- | --- | --- | --- | --- | --- | --- | --- | --- | --- | --- | --- | --- | --- | --- | --- | --- | --- | --- | --- | --- | --- | --- | --- | --- | --- | --- | --- | --- | --- | --- | --- | --- | --- | --- | --- | --- | --- | --- | --- | --- | --- | --- | --- | --- | --- | --- | --- | --- | --- | --- | --- | --- | --- | --- | --- | --- | --- | --- | --- | --- | --- | --- | --- | --- | --- | --- | --- | --- | --- | --- | --- | --- | --- | --- | --- | --- | --- | --- | --- | --- | --- | --- | --- | --- | --- | --- | --- | --- | --- | --- | --- | --- | --- | --- | --- | --- | --- | --- | --- | --- | --- | --- | --- | --- | --- | --- | --- | --- | --- | --- | --- | --- | --- | --- | --- | --- | --- | --- | --- | --- | --- | --- | --- | --- | --- | --- | --- | --- | --- | --- | --- | --- | --- | --- | --- | --- | --- | --- | --- | --- | --- | --- | --- | --- | --- | --- | --- | --- | --- | --- | --- | --- | --- | --- | --- | --- | --- | --- | --- | --- | --- | --- | --- | --- | --- | --- | --- | --- | --- | --- | --- | --- | --- | --- | --- | --- | --- | --- | --- | --- | --- | --- | --- | --- | --- | --- | --- | --- | --- | --- | --- | --- | --- | --- | --- | --- | --- | --- |
| | Sequence name | Weighting | Length | | --- | --- | --- | | AbSI\_ctg\_3356250\_5\_137 | 0.750000 | 15 | | AeAR\_scf\_13\_713\_207 | 0.001100 | 15 | | AeAR\_scf\_22\_2\_103 | 0.004500 | 15 | | AeAR\_scf\_8\_9495\_67 | 0.004500 | 15 | | AeTA\_scf\_3881\_517\_91 | 0.025000 | 15 | | AmTR\_scf\_00011\_26680\_111 | 0.111100 | 15 | | AmTR\_scf\_00157\_302\_75 | 0.111100 | 15 | | AmTR\_scf\_00157\_6341\_123 | 0.111100 | 15 | | AmTR\_scf\_00240\_313\_63 | 0.027800 | 15 | | AmTR\_scf\_00240\_74\_63 | 0.037000 | 15 | | AmTR\_scf\_00270\_119\_90 | 0.055600 | 15 | | AmTR\_scf\_00270\_48\_202 | 0.022200 | 15 | | AmTR\_scf\_00676\_17\_63 | 0.055600 | 15 | | AqCO\_scf\_15\_2726\_82 | 0.125000 | 15 | | AqCO\_scf\_15\_2747\_95 | 0.125000 | 15 | | ArDU\_chr\_01\_262\_90 | 0.005300 | 15 | | ArDU\_chr\_05\_21732\_121 | 0.005300 | 15 | | ArDU\_chr\_08\_250174\_88 | 0.005300 | 15 | | ArIP\_chr\_05\_24674\_60 | 0.006000 | 15 | | ArIP\_chr\_06\_412349\_67 | 0.006000 | 15 | | ArLY\_scf\_2\_10720\_115 | 0.000100 | 15 | | ArLY\_scf\_2\_10720\_190 | 0.000100 | 15 | | ArLY\_scf\_2\_10720\_265 | 0.000100 | 15 | | ArLY\_scf\_2\_10720\_340 | 0.000100 | 15 | | ArLY\_scf\_2\_10720\_415 | 0.000100 | 15 | | ArLY\_scf\_4\_5700\_92 | 0.000900 | 15 | | ArLY\_scf\_5\_79011\_168 | 0.000200 | 15 | | ArLY\_scf\_5\_79011\_76 | 0.000200 | 15 | | ArLY\_scf\_8\_33647\_165 | 0.000100 | 15 | | ArLY\_scf\_8\_33647\_240 | 0.000100 | 15 | | ArLY\_scf\_8\_33647\_315 | 0.000100 | 15 | | ArLY\_scf\_8\_33647\_390 | 0.000100 | 15 | | ArLY\_scf\_8\_63492\_101 | 0.000900 | 15 | | ArLY\_scf\_8\_63506\_67 | 0.000900 | 15 | | ArTH\_chr\_1\_118662\_144 | 0.000400 | 15 | | ArTH\_chr\_1\_42237\_136 | 0.000300 | 15 | | ArTH\_chr\_2\_30548\_78 | 0.000800 | 15 | | ArTH\_chr\_3\_89083\_129 | 0.000200 | 15 | | ArTH\_chr\_3\_89083\_252 | 0.000200 | 15 | | ArTH\_chr\_5\_82449\_59 | 0.000400 | 15 | | ArTH\_chr\_5\_82456\_80 | 0.000800 | 15 | | AzIN\_scf\_17876\_1\_33\_125 | 0.003100 | 15 | | AzIN\_scf\_18451\_1\_5\_139 | 0.001600 | 15 | | AzIN\_scf\_2043\_2\_3\_98 | 0.003100 | 15 | | AzIN\_scf\_2207\_1\_23\_218 | 0.001000 | 15 | | AzIN\_scf\_2708\_1\_30\_115 | 0.003100 | 15 | | AzIN\_scf\_32239\_2\_2\_46 | 0.003100 | 15 | | AzIN\_scf\_4996\_1\_8\_83 | 0.003100 | 15 | | BeNA\_scf\_14434\_160\_88 | 0.012500 | 15 | | BeNA\_scf\_29328\_15\_140 | 0.012500 | 15 | | BeNA\_scf\_7799\_40\_74 | 0.012500 | 15 | | BeVU\_chr\_6.sca003\_7759\_142 | 0.006700 | 15 | | BeVU\_chr\_6.sca003\_7759\_301 | 0.006700 | 15 | | BrDI\_chr\_1\_269090\_87 | 0.035600 | 15 | | BrDI\_chr\_3\_355044\_70 | 0.035600 | 15 | | BrRA\_chr\_01\_110059\_155 | 0.000200 | 15 | | BrRA\_chr\_02\_76978\_71 | 0.000500 | 15 | | BrRA\_chr\_04\_64576\_78 | 0.000500 | 15 | | BrRA\_chr\_04\_76977\_93 | 0.000300 | 15 | | BrRA\_chr\_07\_115013\_55 | 0.000500 | 15 | | BrRA\_chr\_08\_110158\_109 | 0.000500 | 15 | | BrRA\_chr\_09\_146618\_104 | 0.000100 | 15 | | BrRA\_chr\_09\_146618\_227 | 0.000100 | 15 | | BrRA\_chr\_09\_208498\_68 | 0.000500 | 15 | | CaAN\_chr\_02\_42595\_79 | 0.020000 | 15 | | CaCA\_lg\_06\_45335\_70 | 0.002100 | 15 | | CaCA\_lg\_06\_45387\_73 | 0.002100 | 15 | | CaCA\_lg\_10\_34380\_144 | 0.001100 | 15 | | CaCA\_lg\_10\_34423\_105 | 0.002100 | 15 | | CaCA\_scf\_132748\_1420\_95 | 0.002100 | 15 | | CaCA\_scf\_133302\_184\_96 | 0.001100 | 15 | | CaCA\_scf\_133302\_238\_87 | 0.002100 | 15 | | CaCH\_scf\_166197\_23\_67 | 0.020000 | 15 | | CaPA\_ctg\_1124\_137\_76 | 0.011300 | 15 | | CaPA\_ctg\_55\_3048\_126 | 0.011300 | 15 | | CaRU\_scf\_1\_50368\_115 | 0.001300 | 15 | | CaRU\_scf\_4\_63114\_71 | 0.001300 | 15 | | CaRU\_scf\_5\_58226\_210 | 0.000300 | 15 | | CaRU\_scf\_8\_42165\_99 | 0.001300 | 15 | | CaRU\_scf\_8\_42181\_67 | 0.001300 | 15 | | CaSA.Fi\_ctg\_14449324\_3\_108 | 0.006000 | 15 | | CaSA.Fi\_ctg\_14483977\_3\_68 | 0.006000 | 15 | | CaSA.PK\_scf\_43257\_7\_97 | 0.008400 | 15 | | CaSA.PK\_scf\_79209\_8\_112 | 0.008400 | 15 | | CiAR.De\_lg\_7\_10832\_93 | 0.004500 | 15 | | CiAR.Ka\_chr\_7\_27960\_134 | 0.002000 | 15 | | CiAR.Ka\_chr\_7\_28005\_109 | 0.001000 | 15 | | CiAR.Ka\_chr\_7\_28076\_78 | 0.002000 | 15 | | CiAR.Ka\_chr\_8\_49897\_98 | 0.002000 | 15 | | CiCL\_scf\_7\_106244\_148 | 0.006000 | 15 | | CiCL\_scf\_7\_106282\_89 | 0.006000 | 15 | | CiLA\_chr\_11\_92040\_97 | 0.009400 | 15 | | CiLA\_chr\_1\_108400\_72 | 0.009400 | 15 | | CiLA\_chr\_5\_104585\_93 | 0.009400 | 15 | | CiSI\_scf\_00006\_4959\_142 | 0.003000 | 15 | | CiSI\_scf\_00006\_4984\_82 | 0.006000 | 15 | | CuME\_chr\_07\_17885\_102 | 0.009400 | 15 | | CuME\_chr\_08\_112743\_85 | 0.009400 | 15 | | CuME\_chr\_11\_2084\_73 | 0.004700 | 15 | | CuSA\_chr\_4\_27432\_88 | 0.009400 | 15 | | CuSA\_chr\_6\_94302\_79 | 0.009400 | 15 | | DiCA\_scf\_1661\_256\_73 | 0.014800 | 15 | | DiCA\_scf\_234\_262\_166 | 0.004900 | 15 | | DiCA\_scf\_234\_274\_102 | 0.014800 | 15 | | DiCA\_scf\_234\_290\_255 | 0.004900 | 15 | | DiCA\_scf\_6523\_40\_104 | 0.014800 | 15 | | ElGU\_chr\_11\_150068\_67 | 0.039600 | 15 | | ElGU\_chr\_15\_77587\_117 | 0.039600 | 15 | | ElOL\_scf\_00187\_4136\_91 | 0.059300 | 15 | | ElOL\_scf\_00904\_643\_119 | 0.059300 | 15 | | EnVE\_ctg\_22030\_4646726\_1\_11\_105 | 0.105500 | 15 | | EuCA\_ctg\_005169\_46\_147 | 0.002200 | 15 | | EuCA\_ctg\_007856\_3\_119 | 0.002200 | 15 | | EuCA\_ctg\_011137\_153\_190 | 0.001100 | 15 | | EuCA\_ctg\_011619\_37\_99 | 0.002200 | 15 | | EuCA\_ctg\_018397\_20\_173 | 0.000700 | 15 | | EuCA\_ctg\_018397\_212\_119 | 0.000600 | 15 | | EuCA\_ctg\_018397\_212\_97 | 0.000600 | 15 | | EuCA\_ctg\_023070\_58\_158 | 0.000200 | 15 | | EuCA\_ctg\_023070\_58\_224 | 0.000200 | 15 | | EuCA\_ctg\_023070\_58\_290 | 0.000200 | 15 | | EuCA\_ctg\_023293\_14\_95 | 0.001100 | 15 | | EuCA\_ctg\_023562\_269\_122 | 0.001100 | 15 | | EuCA\_ctg\_023562\_313\_178 | 0.001100 | 15 | | EuCA\_ctg\_031336\_177\_140 | 0.002200 | 15 | | EuCA\_ctg\_045219\_166\_101 | 0.002200 | 15 | | EuCA\_ctg\_047439\_35\_90 | 0.002200 | 15 | | EuCA\_ctg\_050958\_143\_116 | 0.001100 | 15 | | EuCA\_ctg\_051094\_155\_177 | 0.001100 | 15 | | EuCA\_ctg\_076399\_2\_90 | 0.002200 | 15 | | EuCA\_scf\_629621\_2\_218 | 0.000700 | 15 | | EuGR\_scf\_4\_135215\_83 | 0.001700 | 15 | | EuGR\_scf\_5\_238225\_119 | 0.000400 | 15 | | EuGR\_scf\_5\_238225\_97 | 0.000400 | 15 | | EuGR\_scf\_5\_238235\_163 | 0.000300 | 15 | | EuGR\_scf\_5\_238372\_114 | 0.000200 | 15 | | EuGR\_scf\_5\_238372\_180 | 0.000200 | 15 | | EuGR\_scf\_5\_238372\_246 | 0.000200 | 15 | | EuGR\_scf\_5\_238372\_312 | 0.000200 | 15 | | EuGR\_scf\_5\_333720\_174 | 0.000400 | 15 | | EuGR\_scf\_5\_333720\_240 | 0.000400 | 15 | | EuGR\_scf\_5\_333937\_158 | 0.000200 | 15 | | EuGR\_scf\_5\_333937\_224 | 0.000200 | 15 | | EuGR\_scf\_5\_333937\_92 | 0.000200 | 15 | | EuGR\_scf\_5\_333963\_158 | 0.000100 | 15 | | EuGR\_scf\_5\_333963\_224 | 0.000100 | 15 | | EuGR\_scf\_5\_333963\_290 | 0.000100 | 15 | | EuGR\_scf\_5\_333963\_356 | 0.000100 | 15 | | EuGR\_scf\_6\_122736\_90 | 0.001700 | 15 | | EuGR\_scf\_6\_192772\_87 | 0.001700 | 15 | | EuGR\_scf\_6\_193279\_158 | 0.001700 | 15 | | EuGR\_scf\_6\_193586\_146 | 0.001700 | 15 | | EuGR\_scf\_8\_143467\_172 | 0.000900 | 15 | | EuGR\_scf\_8\_143712\_234 | 0.000900 | 15 | | EuGR\_scf\_8\_418581\_165 | 0.000900 | 15 | | EuGR\_scf\_8\_418665\_177 | 0.000900 | 15 | | EuGR\_scf\_8\_418792\_134 | 0.000900 | 15 | | EuGR\_scf\_8\_418858\_175 | 0.000900 | 15 | | EuGR\_scf\_9\_63505\_188 | 0.001700 | 15 | | EuGR\_scf\_9\_63890\_152 | 0.001700 | 15 | | EuGR\_scf\_9\_64047\_93 | 0.001700 | 15 | | EuGR\_scf\_9\_64398\_111 | 0.001700 | 15 | | EuSA\_scf\_15\_6297\_98 | 0.001500 | 15 | | EuSA\_scf\_16\_7797\_218 | 0.000400 | 15 | | EuSA\_scf\_5\_91806\_71 | 0.001500 | 15 | | EuSA\_scf\_6\_1164\_58 | 0.000700 | 15 | | EuSA\_scf\_6\_1181\_107 | 0.001500 | 15 | | FrEX\_scf\_14360\_8\_221 | 0.004900 | 15 | | FrEX\_scf\_2186\_496\_114 | 0.004900 | 15 | | FrEX\_scf\_233\_540\_103 | 0.004900 | 15 | | FrEX\_scf\_2682\_616\_230 | 0.009900 | 15 | | FrEX\_scf\_3405\_191\_87 | 0.004900 | 15 | | FrEX\_scf\_5019\_23\_91 | 0.004900 | 15 | | FrEX\_scf\_7878\_74\_73 | 0.009900 | 15 | | FrEX\_scf\_8460\_10\_97 | 0.009900 | 15 | | FrII\_scf\_00065470.1\_27\_141 | 0.010600 | 15 | | FrNI\_ctg\_04327039.1\_4\_149 | 0.002400 | 15 | | FrNI\_scf\_00052972.1\_1\_86 | 0.004800 | 15 | | FrNU\_scf\_00078196.1\_20\_114 | 0.004800 | 15 | | FrNU\_scf\_00085996.1\_9\_87 | 0.004800 | 15 | | FrVE\_lg\_5\_134785\_87 | 0.005300 | 15 | | FrVE\_lg\_6\_109503\_147 | 0.002600 | 15 | | GlMA\_chr\_01\_142070\_103 | 0.000900 | 15 | | GlMA\_chr\_01\_174694\_98 | 0.000400 | 15 | | GlMA\_chr\_03\_19720\_68 | 0.000900 | 15 | | GlMA\_chr\_05\_55551\_80 | 0.000900 | 15 | | GlMA\_chr\_09\_118964\_74 | 0.000900 | 15 | | GlMA\_chr\_11\_11233\_165 | 0.000400 | 15 | | GlMA\_chr\_14\_273581\_86 | 0.000900 | 15 | | GlMA\_chr\_16\_157456\_68 | 0.000900 | 15 | | GlMA\_chr\_17\_184446\_168 | 0.000400 | 15 | | GlMA\_chr\_17\_184560\_164 | 0.000400 | 15 | | GlMA\_chr\_17\_184876\_169 | 0.000400 | 15 | | GnGN\_ctg\_139619\_2\_75 | 1.000000 | 15 | | GoRA\_chr\_03\_227090\_103 | 0.003400 | 15 | | GoRA\_chr\_03\_227128\_82 | 0.003400 | 15 | | GoRA\_chr\_03\_227254\_104 | 0.001700 | 15 | | GoRA\_chr\_03\_227308\_104 | 0.003400 | 15 | | GoRA\_chr\_05\_44934\_87 | 0.003400 | 15 | | GoRA\_chr\_07\_301853\_82 | 0.003400 | 15 | | GoRA\_chr\_09\_302341\_103 | 0.003400 | 15 | | HeBR\_scf\_114295\_32\_84 | 0.002400 | 15 | | HeBR\_scf\_155851\_143\_131 | 0.002400 | 15 | | HeBR\_scf\_155851\_157\_302 | 0.000600 | 15 | | HeBR\_scf\_243691\_91\_279 | 0.000800 | 15 | | HeBR\_scf\_362425\_115\_100 | 0.002400 | 15 | | HoVU\_ctg\_144873\_28\_180 | 0.026700 | 15 | | HoVU\_ctg\_858320\_43\_100 | 0.026700 | 15 | | JaCU\_scf\_02121\_40\_190 | 0.000800 | 15 | | JaCU\_scf\_02121\_40\_338 | 0.000800 | 15 | | JaCU\_scf\_07803\_17\_84 | 0.004500 | 15 | | JaCU\_scf\_14397\_13\_80 | 0.004500 | 15 | | KaFE\_scf\_1366140\_229\_67 | 0.063300 | 15 | | KaFE\_scf\_1373279\_129\_112 | 0.063300 | 15 | | LaSI\_ctg\_347625\_2\_102 | 0.014100 | 15 | | LeAL\_scf\_12\_1266\_85 | 0.001600 | 15 | | LeAL\_scf\_217\_537\_170 | 0.000500 | 15 | | LeAL\_scf\_30\_1510\_69 | 0.001600 | 15 | | LeAL\_scf\_72\_10060\_123 | 0.001600 | 15 | | LiUS\_scf\_1249\_1817\_84 | 0.004700 | 15 | | LiUS\_scf\_1249\_1842\_168 | 0.000600 | 15 | | LiUS\_scf\_1249\_1842\_277 | 0.000600 | 15 | | LiUS\_scf\_1249\_1864\_70 | 0.004700 | 15 | | LiUS\_scf\_2416\_588\_142 | 0.000700 | 15 | | LiUS\_scf\_2416\_588\_244 | 0.000700 | 15 | | LiUS\_scf\_2416\_608\_97 | 0.004700 | 15 | | LiUS\_scf\_488\_1504\_149 | 0.004700 | 15 | | LiUS\_scf\_74\_1464\_96 | 0.004700 | 15 | | LuAN\_scf\_13705\_12\_114 | 0.002200 | 15 | | LuAN\_scf\_14424\_14\_118 | 0.002200 | 15 | | LuAN\_scf\_35677\_71\_92 | 0.004400 | 15 | | LuAN\_scf\_68177\_1\_18\_75 | 0.004400 | 15 | | LuAN\_scf\_76000\_49\_104 | 0.002200 | 15 | | LuAN\_scf\_76092\_52\_84 | 0.004400 | 15 | | LuAN\_scf\_82446\_1\_15\_74 | 0.004400 | 15 | | MaDO\_chr\_15\_179591\_110 | 0.002600 | 15 | | MaDO\_chr\_15\_8414\_76 | 0.005300 | 15 | | MaDO\_chr\_2\_149047\_73 | 0.005300 | 15 | | MaES\_chr\_viii\_36047\_267 | 0.000500 | 15 | | MaES\_chr\_viii\_75422\_87 | 0.002000 | 15 | | MaES\_chr\_x\_67218\_142 | 0.001000 | 15 | | MaES\_chr\_xviii\_47367\_103 | 0.002000 | 15 | | MaES\_chr\_xviii\_47386\_96 | 0.002000 | 15 | | MeTR\_chr\_1\_15289\_85 | 0.001800 | 15 | | MeTR\_chr\_5\_33061\_42 | 0.000900 | 15 | | MeTR\_chr\_5\_33063\_62 | 0.000900 | 15 | | MeTR\_ctg\_59554\_7\_174 | 0.000900 | 15 | | MeTR\_scf\_ac233112.6\_159\_73 | 0.000900 | 15 | | MeTR\_scf\_ac233112.6\_181\_148 | 0.000900 | 15 | | MiGU\_scf\_11\_31507\_103 | 0.019100 | 15 | | MiGU\_scf\_8\_8694\_84 | 0.019100 | 15 | | MiGU\_scf\_8\_8710\_67 | 0.019100 | 15 | | MoNO\_scf\_123\_476\_102 | 0.009400 | 15 | | MoNO\_scf\_467\_1582\_128 | 0.009400 | 15 | | MuAC\_chr\_1\_53460\_83 | 0.033900 | 15 | | MuAC\_chr\_7\_138912\_167 | 0.033900 | 15 | | MuBA\_chr\_1\_126682\_67 | 0.033900 | 15 | | MuBA\_chr\_6\_22482\_94 | 0.033900 | 15 | | MuBA\_chr\_9\_55857\_168 | 0.017000 | 15 | | NeNU\_scf\_00003\_56181\_143 | 0.051100 | 15 | | NeNU\_scf\_00045\_24649\_129 | 0.051100 | 15 | | NeNU\_scf\_00099\_6923\_157 | 0.051100 | 15 | | NeNU\_scf\_00099\_7001\_151 | 0.025600 | 15 | | NiBE\_scf\_126\_1778\_83 | 0.009500 | 15 | | NiBE\_scf\_1697\_919\_107 | 0.009500 | 15 | | NiBE\_scf\_465\_4465\_96 | 0.009500 | 15 | | NiBE\_scf\_58\_1749\_65 | 0.009500 | 15 | | NiBE\_scf\_769\_350\_118 | 0.009500 | 15 | | NiSY\_scf\_161423\_75\_80 | 0.011100 | 15 | | NiSY\_scf\_28833\_905\_71 | 0.011100 | 15 | | NiSY\_scf\_7379\_226\_84 | 0.011100 | 15 | | NiTO\_scf\_15877\_27\_88 | 0.010000 | 15 | | NiTO\_scf\_28409\_188\_97 | 0.010000 | 15 | | NiTO\_scf\_3364\_1611\_90 | 0.010000 | 15 | | OrBR\_chr\_3\_48638\_82 | 0.039600 | 15 | | OrBR\_chr\_7\_105103\_82 | 0.039600 | 15 | | OrSA.In\_chr\_03\_245043\_103 | 0.005600 | 15 | | OrSA.In\_chr\_03\_245043\_77 | 0.005600 | 15 | | OrSA.In\_chr\_06\_125382\_73 | 0.022200 | 15 | | OrSA.Ja\_chr\_03\_226607\_103 | 0.005600 | 15 | | OrSA.Ja\_chr\_03\_226607\_77 | 0.005600 | 15 | | OrSA.Ja\_chr\_06\_127008\_73 | 0.022200 | 15 | | PaHA\_scf\_10\_16779\_92 | 0.029700 | 15 | | PaHA\_scf\_147\_2812\_80 | 0.029700 | 15 | | PaHA\_scf\_45\_6582\_63 | 0.029700 | 15 | | PaVI\_chr\_02b\_133813\_67 | 0.012700 | 15 | | PaVI\_chr\_09a\_177585\_81 | 0.006400 | 15 | | PaVI\_chr\_09b\_67384\_118 | 0.012700 | 15 | | PaVI\_ctg\_16368\_16\_66 | 0.012700 | 15 | | PaVI\_ctg\_356223\_8\_78 | 0.012700 | 15 | | PhDA\_scf\_000109\_280\_135 | 0.052700 | 15 | | PhDA\_scf\_001357\_165\_88 | 0.052700 | 15 | | PhHE\_scf\_01000933\_232\_72 | 0.029700 | 15 | | PhHE\_scf\_01003054\_1356\_67 | 0.029700 | 15 | | PhHE\_scf\_01007655\_65\_77 | 0.029700 | 15 | | PhVU\_chr\_02\_5513\_132 | 0.001500 | 15 | | PhVU\_chr\_03\_128027\_147 | 0.000700 | 15 | | PhVU\_chr\_03\_128057\_103 | 0.000700 | 15 | | PhVU\_chr\_03\_128143\_135 | 0.001500 | 15 | | PhVU\_chr\_09\_158890\_94 | 0.001500 | 15 | | PiAB\_ctg\_3289\_442\_136 | 0.070300 | 15 | | PiAB\_ctg\_9991140\_55\_135 | 0.070300 | 15 | | PiGL\_ctg\_163248640\_0\_5\_163 | 0.030100 | 15 | | PiGL\_ctg\_165564112\_0\_3\_147 | 0.030100 | 15 | | PiGL\_ctg\_167910995\_40\_6\_153 | 0.030100 | 15 | | PiGL\_ctg\_168012670\_1\_14\_153 | 0.030100 | 15 | | PiSY\_ctg\_10265915\_4\_149 | 0.210900 | 15 | | PiTA\_ctg\_30790360\_8\_155 | 0.020100 | 15 | | PiTA\_scf\_185885\_469\_129 | 0.020100 | 15 | | PiTA\_scf\_365587\_807\_171 | 0.020100 | 15 | | PiTA\_scf\_789033\_82\_129 | 0.020100 | 15 | | PiTA\_scf\_888115\_424\_135 | 0.020100 | 15 | | PiTA\_scf\_t3921\_3572\_129 | 0.020100 | 15 | | PiTA\_scf\_t7716\_19726\_147 | 0.020100 | 15 | | PoEU\_scf\_2.1\_3612\_75 | 0.006300 | 15 | | PoEU\_scf\_234.1\_203\_196 | 0.001300 | 15 | | PoEU\_scf\_234.1\_203\_353 | 0.001300 | 15 | | PoEU\_scf\_354.1\_775\_121 | 0.006300 | 15 | | PoEU\_scf\_354.1\_893\_263 | 0.002100 | 15 | | PoEU\_scf\_623.1\_696\_263 | 0.002100 | 15 | | PoTR\_chr\_05\_109193\_108 | 0.001000 | 15 | | PoTR\_chr\_05\_109193\_222 | 0.001000 | 15 | | PoTR\_chr\_07\_72674\_120 | 0.000600 | 15 | | PoTR\_chr\_07\_72674\_264 | 0.000600 | 15 | | PoTR\_chr\_07\_72674\_421 | 0.000600 | 15 | | PoTR\_chr\_07\_72858\_120 | 0.000600 | 15 | | PoTR\_chr\_07\_72858\_264 | 0.000600 | 15 | | PoTR\_chr\_07\_72858\_453 | 0.000600 | 15 | | PoTR\_chr\_07\_72919\_117 | 0.005100 | 15 | | PoTR\_scf\_376\_64\_80 | 0.005100 | 15 | | PrMU\_chr\_8\_31248\_131 | 0.003200 | 15 | | PrMU\_chr\_8\_31271\_121 | 0.006300 | 15 | | PrPE\_scf\_7\_52240\_107 | 0.005300 | 15 | | PrPE\_scf\_7\_52257\_80 | 0.005300 | 15 | | PyBR\_scf\_145.0\_80\_113 | 0.001600 | 15 | | PyBR\_scf\_154.0\_468\_76 | 0.001600 | 15 | | PyBR\_scf\_197.0\_2325\_105 | 0.001600 | 15 | | PyBR\_scf\_4.0\_9791\_132 | 0.000800 | 15 | | PyBR\_scf\_520.0\_2920\_105 | 0.001600 | 15 | | PyBR\_scf\_520.0\_2966\_132 | 0.001600 | 15 | | PyCO\_scf\_00016\_3652\_73 | 0.002600 | 15 | | PyCO\_scf\_00781\_604\_101 | 0.002600 | 15 | | PyCO\_scf\_03185\_22\_133 | 0.001300 | 15 | | RiCO\_ctg\_29676\_38\_114 | 0.007000 | 15 | | RiCO\_ctg\_29676\_57\_280 | 0.001800 | 15 | | RiCO\_ctg\_29676\_82\_137 | 0.007000 | 15 | | ScPA\_chr\_1-1\_34613\_59 | 0.001300 | 15 | | ScPA\_chr\_2-4\_32069\_67 | 0.000700 | 15 | | ScPA\_chr\_2-4\_32083\_81 | 0.001300 | 15 | | ScPA\_chr\_4-6\_41886\_75 | 0.001300 | 15 | | ScPA\_chr\_5-1\_14490\_221 | 0.000300 | 15 | | SeIN\_lg\_15\_9787\_116 | 0.005000 | 15 | | SeIN\_lg\_15\_9817\_70 | 0.010000 | 15 | | SeIN\_lg\_1\_108355\_72 | 0.010000 | 15 | | SeIN\_lg\_8\_120425\_83 | 0.010000 | 15 | | SeIT\_chr\_4\_167625\_86 | 0.039600 | 15 | | SeIT\_chr\_9\_300249\_77 | 0.039600 | 15 | | SiIR\_scf\_1042\_47125\_77 | 0.001000 | 15 | | SiIR\_scf\_20\_535\_212 | 0.000200 | 15 | | SiIR\_scf\_24\_20762\_72 | 0.001000 | 15 | | SiIR\_scf\_43\_14573\_148 | 0.001000 | 15 | | SiIR\_scf\_8\_11146\_88 | 0.000500 | 15 | | SoBI\_chr\_01\_202134\_105 | 0.022200 | 15 | | SoBI\_chr\_01\_340955\_81 | 0.022200 | 15 | | SoBI\_chr\_07\_234975\_85 | 0.022200 | 15 | | SoLY\_chr\_03\_22772\_114 | 0.015000 | 15 | | SoLY\_chr\_12\_152273\_84 | 0.015000 | 15 | | SoPI\_ctg\_6592119\_8\_84 | 0.015000 | 15 | | SoTU\_chr\_02\_104011\_72 | 0.016700 | 15 | | SoTU\_chr\_03\_273390\_149 | 0.016700 | 15 | | SpOL\_scf\_21396\_72\_110 | 0.001400 | 15 | | SpOL\_scf\_21396\_72\_206 | 0.001400 | 15 | | SpOL\_scf\_21396\_72\_317 | 0.001400 | 15 | | SpOL\_scf\_21396\_72\_445 | 0.001400 | 15 | | SpOL\_scf\_21396\_72\_541 | 0.001400 | 15 | | SpOL\_scf\_24951\_60\_82 | 0.019100 | 15 | | SpOL\_scf\_25714\_168\_284 | 0.003800 | 15 | | SpOL\_scf\_25714\_168\_412 | 0.003800 | 15 | | SpPO\_chr\_4\_56998\_137 | 0.375000 | 15 | | TaHA\_scf\_10\_1346\_150 | 0.002100 | 15 | | TaHA\_scf\_12\_14888\_70 | 0.004200 | 15 | | TaHA\_scf\_33\_10698\_96 | 0.004200 | 15 | | TaHA\_scf\_3\_40844\_115 | 0.004200 | 15 | | ThCA.Cr\_chr\_00\_28530\_156 | 0.003100 | 15 | | ThCA.Cr\_chr\_01\_161945\_213 | 0.006300 | 15 | | ThCA.Cr\_chr\_08\_1531\_109 | 0.006300 | 15 | | ThCA.Ma\_chr\_1\_202452\_84 | 0.006300 | 15 | | ThCA.Ma\_chr\_1\_202475\_156 | 0.003100 | 15 | | ThCA.Ma\_chr\_3\_92411\_77 | 0.006300 | 15 | | ThCA.Ma\_chr\_8\_1804\_121 | 0.006300 | 15 | | TrUR\_scf\_73978\_126\_81 | 0.033400 | 15 | | ViAN\_ctg\_11585\_0\_25\_93 | 0.002000 | 15 | | ViAN\_scf\_14509\_12\_115 | 0.001000 | 15 | | ViAN\_scf\_14509\_28\_150 | 0.001000 | 15 | | ViAN\_scf\_5923\_20\_108 | 0.002000 | 15 | | ZeMA\_chr\_2\_813531\_110 | 0.035600 | 15 | | ZeMA\_chr\_7\_545502\_109 | 0.035600 | 15 | | | Sequence name | Weighting | Length | | --- | --- | --- | | AeAR\_scf\_13\_713\_103 | 0.001100 | 15 | | AeAR\_scf\_13\_713\_51 | 0.001100 | 15 | | AeAR\_scf\_8\_9481\_99 | 0.004500 | 15 | | AeAR\_scf\_9\_3455\_78 | 0.004500 | 15 | | AeTA\_scf\_55155\_109\_136 | 0.025000 | 15 | | AmTR\_scf\_00157\_136\_112 | 0.111100 | 15 | | AmTR\_scf\_00157\_397\_87 | 0.111100 | 15 | | AmTR\_scf\_00240\_313\_117 | 0.027800 | 15 | | AmTR\_scf\_00240\_313\_90 | 0.027800 | 15 | | AmTR\_scf\_00240\_74\_90 | 0.037000 | 15 | | AmTR\_scf\_00270\_48\_148 | 0.022200 | 15 | | AmTR\_scf\_00270\_48\_229 | 0.022200 | 15 | | AmTR\_scf\_00676\_17\_90 | 0.055600 | 15 | | AqCO\_scf\_15\_2734\_84 | 0.125000 | 15 | | AqCO\_scf\_63\_6175\_77 | 0.125000 | 15 | | ArDU\_chr\_05\_21700\_71 | 0.005300 | 15 | | ArDU\_chr\_05\_606935\_76 | 0.005300 | 15 | | ArIP\_chr\_01\_784210\_91 | 0.006000 | 15 | | ArIP\_chr\_05\_24681\_129 | 0.006000 | 15 | | ArIP\_chr\_07\_426831\_88 | 0.006000 | 15 | | ArLY\_scf\_2\_10720\_140 | 0.000100 | 15 | | ArLY\_scf\_2\_10720\_215 | 0.000100 | 15 | | ArLY\_scf\_2\_10720\_290 | 0.000100 | 15 | | ArLY\_scf\_2\_10720\_365 | 0.000100 | 15 | | ArLY\_scf\_2\_10720\_440 | 0.000100 | 15 | | ArLY\_scf\_4\_85025\_74 | 0.000900 | 15 | | ArLY\_scf\_5\_79011\_220 | 0.000200 | 15 | | ArLY\_scf\_8\_33647\_115 | 0.000100 | 15 | | ArLY\_scf\_8\_33647\_190 | 0.000100 | 15 | | ArLY\_scf\_8\_33647\_265 | 0.000100 | 15 | | ArLY\_scf\_8\_33647\_340 | 0.000100 | 15 | | ArLY\_scf\_8\_33647\_415 | 0.000100 | 15 | | ArLY\_scf\_8\_63500\_61 | 0.000500 | 15 | | ArLY\_scf\_8\_63513\_83 | 0.000900 | 15 | | ArTH\_chr\_1\_36231\_73 | 0.000800 | 15 | | ArTH\_chr\_1\_42237\_161 | 0.000300 | 15 | | ArTH\_chr\_2\_30559\_119 | 0.000800 | 15 | | ArTH\_chr\_3\_89083\_181 | 0.000200 | 15 | | ArTH\_chr\_3\_89083\_77 | 0.000200 | 15 | | ArTH\_chr\_5\_82449\_88 | 0.000400 | 15 | | AzIN\_scf\_17876\_1\_26\_121 | 0.001600 | 15 | | AzIN\_scf\_17876\_1\_40\_121 | 0.003100 | 15 | | AzIN\_scf\_2043\_2\_19\_119 | 0.003100 | 15 | | AzIN\_scf\_2207\_1\_23\_105 | 0.001000 | 15 | | AzIN\_scf\_23221\_3\_16\_83 | 0.003100 | 15 | | AzIN\_scf\_2708\_2\_45\_98 | 0.003100 | 15 | | AzIN\_scf\_42817\_1\_18\_125 | 0.003100 | 15 | | AzIN\_scf\_8125\_2\_25\_123 | 0.003100 | 15 | | BeNA\_scf\_16996\_34\_116 | 0.012500 | 15 | | BeNA\_scf\_47625\_4\_80 | 0.012500 | 15 | | BeVU\_chr\_5.sca002\_29411\_110 | 0.026700 | 15 | | BeVU\_chr\_6.sca003\_7759\_186 | 0.006700 | 15 | | BeVU\_chr\_6.sca003\_7802\_97 | 0.026700 | 15 | | BrDI\_chr\_1\_402044\_63 | 0.035600 | 15 | | BrDI\_chr\_4\_169807\_80 | 0.035600 | 15 | | BrRA\_chr\_01\_110059\_174 | 0.000200 | 15 | | BrRA\_chr\_03\_161871\_75 | 0.000500 | 15 | | BrRA\_chr\_04\_76968\_93 | 0.000500 | 15 | | BrRA\_chr\_05\_14020\_76 | 0.000500 | 15 | | BrRA\_chr\_07\_115031\_67 | 0.000500 | 15 | | BrRA\_chr\_08\_95395\_224 | 0.000500 | 15 | | BrRA\_chr\_09\_146618\_156 | 0.000100 | 15 | | BrRA\_chr\_09\_146618\_52 | 0.000100 | 15 | | CaAN\_chr\_02\_414002\_77 | 0.020000 | 15 | | CaAN\_chr\_07\_708661\_67 | 0.020000 | 15 | | CaCA\_lg\_06\_45369\_132 | 0.001100 | 15 | | CaCA\_lg\_06\_45397\_123 | 0.002100 | 15 | | CaCA\_lg\_10\_34380\_98 | 0.001100 | 15 | | CaCA\_lg\_11\_231024\_76 | 0.002100 | 15 | | CaCA\_scf\_132880\_33\_64 | 0.002100 | 15 | | CaCA\_scf\_133302\_222\_135 | 0.001100 | 15 | | CaCH\_scf\_102917\_258\_77 | 0.020000 | 15 | | CaCH\_scf\_179831\_16\_82 | 0.020000 | 15 | | CaPA\_ctg\_55\_3031\_51 | 0.011300 | 15 | | CaPA\_ctg\_64\_5934\_76 | 0.011300 | 15 | | CaRU\_scf\_4\_3227\_98 | 0.001300 | 15 | | CaRU\_scf\_5\_58226\_106 | 0.000300 | 15 | | CaRU\_scf\_5\_58226\_229 | 0.000300 | 15 | | CaRU\_scf\_8\_42174\_72 | 0.001300 | 15 | | CaSA.Fi\_ctg\_10459586\_3\_126 | 0.006000 | 15 | | CaSA.Fi\_ctg\_14469617\_5\_97 | 0.006000 | 15 | | CaSA.Fi\_ctg\_5361018\_3\_115 | 0.006000 | 15 | | CaSA.PK\_scf\_47871\_7\_92 | 0.008400 | 15 | | CiAR.De\_lg\_3\_37933\_61 | 0.004500 | 15 | | CiAR.De\_scf\_04274\_79\_78 | 0.004500 | 15 | | CiAR.Ka\_chr\_7\_27979\_119 | 0.001000 | 15 | | CiAR.Ka\_chr\_7\_28005\_62 | 0.001000 | 15 | | CiAR.Ka\_chr\_7\_28093\_139 | 0.002000 | 15 | | CiCL\_scf\_2\_23313\_84 | 0.006000 | 15 | | CiCL\_scf\_7\_106263\_142 | 0.003000 | 15 | | CiCL\_scf\_7\_106293\_82 | 0.006000 | 15 | | CiLA\_chr\_1\_108332\_117 | 0.009400 | 15 | | CiLA\_chr\_2\_56957\_121 | 0.004700 | 15 | | CiSI\_scf\_00002\_6043\_93 | 0.006000 | 15 | | CiSI\_scf\_00006\_4959\_95 | 0.003000 | 15 | | CiSI\_scf\_00028\_3838\_84 | 0.006000 | 15 | | CuME\_chr\_07\_6955\_127 | 0.009400 | 15 | | CuME\_chr\_08\_112760\_85 | 0.009400 | 15 | | CuSA\_chr\_2\_34874\_112 | 0.004700 | 15 | | CuSA\_chr\_4\_70129\_127 | 0.009400 | 15 | | CuSA\_chr\_6\_94315\_123 | 0.009400 | 15 | | DiCA\_scf\_204\_432\_65 | 0.014800 | 15 | | DiCA\_scf\_234\_262\_202 | 0.004900 | 15 | | DiCA\_scf\_234\_290\_177 | 0.004900 | 15 | | DiCA\_scf\_234\_310\_104 | 0.014800 | 15 | | DiCA\_scf\_6587\_48\_100 | 0.014800 | 15 | | ElGU\_chr\_15\_38349\_1292 | 0.039600 | 15 | | ElGU\_chr\_3\_71946\_129 | 0.039600 | 15 | | ElOL\_scf\_00542\_907\_90 | 0.059300 | 15 | | EnVE\_ctg\_22030\_27790\_1\_28\_156 | 0.052700 | 15 | | EnVE\_scf\_22030\_78542\_116\_48 | 0.105500 | 15 | | EuCA\_ctg\_007672\_9\_131 | 0.001100 | 15 | | EuCA\_ctg\_010936\_1\_114 | 0.001100 | 15 | | EuCA\_ctg\_011137\_153\_234 | 0.001100 | 15 | | EuCA\_ctg\_013901\_36\_103 | 0.002200 | 15 | | EuCA\_ctg\_018397\_20\_195 | 0.000700 | 15 | | EuCA\_ctg\_018397\_212\_141 | 0.000600 | 15 | | EuCA\_ctg\_023070\_58\_114 | 0.000200 | 15 | | EuCA\_ctg\_023070\_58\_180 | 0.000200 | 15 | | EuCA\_ctg\_023070\_58\_246 | 0.000200 | 15 | | EuCA\_ctg\_023070\_58\_92 | 0.000200 | 15 | | EuCA\_ctg\_023562\_184\_133 | 0.001100 | 15 | | EuCA\_ctg\_023562\_269\_166 | 0.001100 | 15 | | EuCA\_ctg\_025488\_37\_129 | 0.002200 | 15 | | EuCA\_ctg\_034442\_93\_105 | 0.001100 | 15 | | EuCA\_ctg\_045219\_187\_111 | 0.002200 | 15 | | EuCA\_ctg\_049989\_43\_158 | 0.002200 | 15 | | EuCA\_ctg\_050958\_143\_95 | 0.001100 | 15 | | EuCA\_ctg\_052267\_53\_108 | 0.002200 | 15 | | EuCA\_scf\_629621\_2\_174 | 0.000700 | 15 | | EuCA\_scf\_751148\_11\_90 | 0.002200 | 15 | | EuGR\_scf\_566\_96\_119 | 0.001700 | 15 | | EuGR\_scf\_5\_238225\_141 | 0.000400 | 15 | | EuGR\_scf\_5\_238235\_119 | 0.000300 | 15 | | EuGR\_scf\_5\_238235\_75 | 0.000300 | 15 | | EuGR\_scf\_5\_238372\_136 | 0.000200 | 15 | | EuGR\_scf\_5\_238372\_202 | 0.000200 | 15 | | EuGR\_scf\_5\_238372\_268 | 0.000200 | 15 | | EuGR\_scf\_5\_238372\_92 | 0.000200 | 15 | | EuGR\_scf\_5\_333720\_196 | 0.000400 | 15 | | EuGR\_scf\_5\_333937\_114 | 0.000200 | 15 | | EuGR\_scf\_5\_333937\_180 | 0.000200 | 15 | | EuGR\_scf\_5\_333937\_246 | 0.000200 | 15 | | EuGR\_scf\_5\_333963\_114 | 0.000100 | 15 | | EuGR\_scf\_5\_333963\_180 | 0.000100 | 15 | | EuGR\_scf\_5\_333963\_246 | 0.000100 | 15 | | EuGR\_scf\_5\_333963\_312 | 0.000100 | 15 | | EuGR\_scf\_5\_333963\_92 | 0.000100 | 15 | | EuGR\_scf\_6\_1772\_116 | 0.000900 | 15 | | EuGR\_scf\_6\_193234\_113 | 0.001700 | 15 | | EuGR\_scf\_6\_193319\_146 | 0.001700 | 15 | | EuGR\_scf\_6\_1947\_119 | 0.001700 | 15 | | EuGR\_scf\_8\_143467\_216 | 0.000900 | 15 | | EuGR\_scf\_8\_418547\_117 | 0.001700 | 15 | | EuGR\_scf\_8\_418605\_108 | 0.001700 | 15 | | EuGR\_scf\_8\_418753\_122 | 0.000900 | 15 | | EuGR\_scf\_8\_418792\_178 | 0.000900 | 15 | | EuGR\_scf\_8\_418948\_174 | 0.000900 | 15 | | EuGR\_scf\_9\_63568\_136 | 0.001700 | 15 | | EuGR\_scf\_9\_63899\_121 | 0.001700 | 15 | | EuGR\_scf\_9\_64228\_109 | 0.001700 | 15 | | EuSA\_scf\_10\_49408\_67 | 0.001500 | 15 | | EuSA\_scf\_16\_7797\_147 | 0.000400 | 15 | | EuSA\_scf\_16\_7797\_95 | 0.000400 | 15 | | EuSA\_scf\_5\_91852\_71 | 0.001500 | 15 | | EuSA\_scf\_6\_1164\_87 | 0.000700 | 15 | | EuSA\_scf\_7\_63503\_123 | 0.001500 | 15 | | FrEX\_scf\_1891\_617\_90 | 0.009900 | 15 | | FrEX\_scf\_2186\_496\_76 | 0.004900 | 15 | | FrEX\_scf\_233\_540\_141 | 0.004900 | 15 | | FrEX\_scf\_3403\_77\_99 | 0.009900 | 15 | | FrEX\_scf\_3900\_232\_91 | 0.009900 | 15 | | FrEX\_scf\_5423\_116\_91 | 0.009900 | 15 | | FrEX\_scf\_7878\_96\_103 | 0.009900 | 15 | | FrII\_scf\_00001632.1\_18\_107 | 0.010600 | 15 | | FrII\_scf\_00066163.1\_12\_118 | 0.010600 | 15 | | FrNI\_ctg\_04412679.1\_6\_87 | 0.004800 | 15 | | FrNI\_scf\_00104625.1\_5\_114 | 0.004800 | 15 | | FrNU\_scf\_00078196.1\_34\_107 | 0.002400 | 15 | | FrNU\_scf\_00093857.1\_6\_101 | 0.004800 | 15 | | FrVE\_lg\_6\_109489\_114 | 0.005300 | 15 | | FrVE\_lg\_6\_109514\_101 | 0.005300 | 15 | | GlMA\_chr\_01\_174682\_117 | 0.000900 | 15 | | GlMA\_chr\_01\_174715\_86 | 0.000900 | 15 | | GlMA\_chr\_05\_53925\_133 | 0.000400 | 15 | | GlMA\_chr\_05\_55563\_127 | 0.000900 | 15 | | GlMA\_chr\_11\_11219\_73 | 0.000900 | 15 | | GlMA\_chr\_11\_11247\_72 | 0.000900 | 15 | | GlMA\_chr\_16\_156497\_78 | 0.000900 | 15 | | GlMA\_chr\_17\_113157\_91 | 0.000900 | 15 | | GlMA\_chr\_17\_184482\_126 | 0.000900 | 15 | | GlMA\_chr\_17\_184765\_74 | 0.000900 | 15 | | GlMA\_chr\_17\_185198\_110 | 0.000900 | 15 | | GoRA\_chr\_03\_227048\_78 | 0.003400 | 15 | | GoRA\_chr\_03\_227119\_76 | 0.003400 | 15 | | GoRA\_chr\_03\_227185\_102 | 0.003400 | 15 | | GoRA\_chr\_03\_227254\_151 | 0.001700 | 15 | | GoRA\_chr\_03\_4174\_104 | 0.003400 | 15 | | GoRA\_chr\_07\_252127\_82 | 0.003400 | 15 | | GoRA\_chr\_07\_301859\_85 | 0.003400 | 15 | | GoRA\_chr\_09\_319933\_80 | 0.003400 | 15 | | HeBR\_scf\_119199\_95\_60 | 0.002400 | 15 | | HeBR\_scf\_155851\_157\_109 | 0.000600 | 15 | | HeBR\_scf\_155851\_157\_336 | 0.000600 | 15 | | HeBR\_scf\_243691\_91\_313 | 0.000800 | 15 | | HeBR\_scf\_413830\_5\_75 | 0.002400 | 15 | | HoVU\_ctg\_1982316\_28\_72 | 0.026700 | 15 | | HoVU\_ctg\_96118\_25\_175 | 0.026700 | 15 | | JaCU\_scf\_02121\_40\_249 | 0.000800 | 15 | | JaCU\_scf\_02121\_40\_73 | 0.000800 | 15 | | JaCU\_scf\_08269\_85\_70 | 0.004500 | 15 | | JaCU\_scf\_19785\_4\_77 | 0.004500 | 15 | | KaFE\_scf\_1368498\_122\_83 | 0.063300 | 15 | | LaSI\_ctg\_301883\_1\_91 | 0.014100 | 15 | | LaSI\_scf\_25684\_14\_96 | 0.014100 | 15 | | LeAL\_scf\_17\_1975\_95 | 0.001600 | 15 | | LeAL\_scf\_217\_537\_99 | 0.000500 | 15 | | LeAL\_scf\_30\_1513\_76 | 0.001600 | 15 | | LeAL\_scf\_72\_10083\_80 | 0.001600 | 15 | | LiUS\_scf\_1249\_1842\_121 | 0.000600 | 15 | | LiUS\_scf\_1249\_1842\_201 | 0.000600 | 15 | | LiUS\_scf\_1249\_1842\_311 | 0.000600 | 15 | | LiUS\_scf\_2416\_569\_84 | 0.004700 | 15 | | LiUS\_scf\_2416\_588\_171 | 0.000700 | 15 | | LiUS\_scf\_2416\_588\_279 | 0.000700 | 15 | | LiUS\_scf\_464\_9234\_106 | 0.004700 | 15 | | LiUS\_scf\_650\_806\_167 | 0.004700 | 15 | | LoJA\_chr\_2\_54742\_107 | 0.015800 | 15 | | LuAN\_scf\_13705\_12\_160 | 0.002200 | 15 | | LuAN\_scf\_14424\_14\_163 | 0.002200 | 15 | | LuAN\_scf\_51909\_121\_70 | 0.004400 | 15 | | LuAN\_scf\_72448\_2\_5\_85 | 0.004400 | 15 | | LuAN\_scf\_76000\_49\_148 | 0.002200 | 15 | | LuAN\_scf\_79499\_72\_77 | 0.004400 | 15 | | LuAN\_scf\_92387\_1\_2\_84 | 0.004400 | 15 | | MaDO\_chr\_15\_179591\_143 | 0.002600 | 15 | | MaDO\_chr\_2\_149034\_127 | 0.002600 | 15 | | MaES\_chr\_viii\_35996\_108 | 0.002000 | 15 | | MaES\_chr\_viii\_36047\_301 | 0.000500 | 15 | | MaES\_chr\_viii\_75440\_84 | 0.002000 | 15 | | MaES\_chr\_x\_67227\_73 | 0.002000 | 15 | | MaES\_chr\_xviii\_47380\_625 | 0.001000 | 15 | | MaES\_chr\_xviii\_47391\_89 | 0.002000 | 15 | | MeTR\_chr\_5\_16827\_84 | 0.001800 | 15 | | MeTR\_chr\_5\_33061\_94 | 0.000900 | 15 | | MeTR\_chr\_8\_74208\_73 | 0.001800 | 15 | | MeTR\_ctg\_74316\_18\_95 | 0.001800 | 15 | | MeTR\_scf\_ac233112.6\_165\_83 | 0.001800 | 15 | | MeTR\_scf\_ac233112.6\_194\_63 | 0.001800 | 15 | | MiGU\_scf\_14\_91504\_107 | 0.019100 | 15 | | MiGU\_scf\_8\_8701\_110 | 0.009500 | 15 | | MiGU\_scf\_8\_8739\_79 | 0.019100 | 15 | | MoNO\_scf\_123\_489\_89 | 0.009400 | 15 | | MoNO\_scf\_7113\_6\_143 | 0.009400 | 15 | | MuAC\_chr\_3\_185713\_108 | 0.033900 | 15 | | MuAC\_chr\_9\_62965\_148 | 0.033900 | 15 | | MuBA\_chr\_1\_49638\_81 | 0.033900 | 15 | | MuBA\_chr\_7\_123907\_170 | 0.033900 | 15 | | MuBA\_chr\_un\_random\_228501\_97 | 0.033900 | 15 | | NeNU\_scf\_00003\_56217\_113 | 0.051100 | 15 | | NeNU\_scf\_00096\_5431\_74 | 0.051100 | 15 | | NeNU\_scf\_00099\_6989\_89 | 0.051100 | 15 | | NeNU\_scf\_00099\_7938\_71 | 0.051100 | 15 | | NiBE\_scf\_126\_2333\_114 | 0.009500 | 15 | | NiBE\_scf\_2639\_1487\_70 | 0.009500 | 15 | | NiBE\_scf\_465\_4629\_89 | 0.009500 | 15 | | NiBE\_scf\_606\_2401\_80 | 0.009500 | 15 | | NiSY\_scf\_12155\_205\_137 | 0.011100 | 15 | | NiSY\_scf\_25424\_227\_110 | 0.011100 | 15 | | NiSY\_scf\_35720\_248\_113 | 0.011100 | 15 | | NiTO\_ctg\_18607\_232\_75 | 0.010000 | 15 | | NiTO\_scf\_16588\_177\_82 | 0.010000 | 15 | | NiTO\_scf\_31316\_86\_110 | 0.010000 | 15 | | NiTO\_scf\_9645\_1400\_73 | 0.010000 | 15 | | OrBR\_chr\_3\_48668\_173 | 0.039600 | 15 | | OrBR\_chr\_9\_30468\_70 | 0.039600 | 15 | | OrSA.In\_chr\_03\_245043\_129 | 0.005600 | 15 | | OrSA.In\_chr\_03\_67955\_85 | 0.022200 | 15 | | OrSA.In\_chr\_08\_97569\_88 | 0.022200 | 15 | | OrSA.Ja\_chr\_03\_226607\_129 | 0.005600 | 15 | | OrSA.Ja\_chr\_03\_64050\_85 | 0.022200 | 15 | | OrSA.Ja\_chr\_08\_91083\_88 | 0.022200 | 15 | | PaHA\_scf\_147\_2797\_121 | 0.014800 | 15 | | PaHA\_scf\_31\_12006\_66 | 0.029700 | 15 | | PaVI\_chr\_02a\_355897\_71 | 0.012700 | 15 | | PaVI\_chr\_02b\_133822\_68 | 0.012700 | 15 | | PaVI\_chr\_09b\_67367\_149 | 0.006400 | 15 | | PaVI\_ctg\_02119\_158\_71 | 0.012700 | 15 | | PaVI\_ctg\_173587\_8\_80 | 0.012700 | 15 | | PaVI\_ctg\_85087\_29\_82 | 0.012700 | 15 | | PhDA\_scf\_000134\_4732\_559 | 0.052700 | 15 | | PhDA\_scf\_001357\_177\_113 | 0.052700 | 15 | | PhHE\_scf\_01001027\_375\_77 | 0.029700 | 15 | | PhHE\_scf\_01003396\_771\_81 | 0.029700 | 15 | | PhVU\_chr\_01\_290172\_90 | 0.001500 | 15 | | PhVU\_chr\_02\_5535\_105 | 0.001500 | 15 | | PhVU\_chr\_03\_128036\_114 | 0.000700 | 15 | | PhVU\_chr\_03\_128057\_149 | 0.000700 | 15 | | PhVU\_chr\_03\_136183\_78 | 0.001500 | 15 | | PiAB\_ctg\_18434\_18\_153 | 0.070300 | 15 | | PiAB\_ctg\_433845\_9\_153 | 0.070300 | 15 | | PiGL\_ctg\_161449300\_0\_17\_163 | 0.030100 | 15 | | PiGL\_ctg\_164078179\_0\_4\_137 | 0.030100 | 15 | | PiGL\_ctg\_167670474\_0\_12\_135 | 0.030100 | 15 | | PiGL\_ctg\_167936109\_6\_41\_156 | 0.030100 | 15 | | PiGL\_ctg\_168152959\_3\_5\_153 | 0.030100 | 15 | | PiSY\_ctg\_6288925\_3\_164 | 0.210900 | 15 | | PiTA\_ctg\_32464818\_318\_171 | 0.020100 | 15 | | PiTA\_scf\_274065\_340\_153 | 0.020100 | 15 | | PiTA\_scf\_44548\_262\_148 | 0.020100 | 15 | | PiTA\_scf\_880179.2\_309\_110 | 0.020100 | 15 | | PiTA\_scf\_901296\_275\_360 | 0.020100 | 15 | | PiTA\_scf\_t3921\_878\_153 | 0.020100 | 15 | | PiTA\_scf\_t8959\_80\_129 | 0.020100 | 15 | | PoEU\_scf\_234.1\_139\_128 | 0.006300 | 15 | | PoEU\_scf\_234.1\_203\_228 | 0.001300 | 15 | | PoEU\_scf\_234.1\_307\_97 | 0.006300 | 15 | | PoEU\_scf\_354.1\_893\_139 | 0.002100 | 15 | | PoEU\_scf\_623.1\_696\_139 | 0.002100 | 15 | | PoTR\_chr\_04\_65116\_80 | 0.005100 | 15 | | PoTR\_chr\_05\_109193\_142 | 0.001000 | 15 | | PoTR\_chr\_05\_109193\_304 | 0.001000 | 15 | | PoTR\_chr\_07\_72674\_146 | 0.000600 | 15 | | PoTR\_chr\_07\_72674\_340 | 0.000600 | 15 | | PoTR\_chr\_07\_72674\_462 | 0.000600 | 15 | | PoTR\_chr\_07\_72858\_146 | 0.000600 | 15 | | PoTR\_chr\_07\_72858\_340 | 0.000600 | 15 | | PoTR\_chr\_07\_72858\_497 | 0.000600 | 15 | | PoTR\_chr\_07\_72949\_105 | 0.005100 | 15 | | PrMU\_chr\_2\_241459\_151 | 0.006300 | 15 | | PrMU\_chr\_8\_31248\_99 | 0.003200 | 15 | | PrPE\_scf\_1\_122064\_74 | 0.005300 | 15 | | PrPE\_scf\_7\_52252\_138 | 0.002600 | 15 | | PrPE\_scf\_7\_52280\_197 | 0.005300 | 15 | | PyBR\_scf\_145.0\_93\_102 | 0.001600 | 15 | | PyBR\_scf\_190.0\_3108\_129 | 0.001600 | 15 | | PyBR\_scf\_197.0\_2336\_118 | 0.001600 | 15 | | PyBR\_scf\_4.0\_9791\_99 | 0.000800 | 15 | | PyBR\_scf\_520.0\_2943\_100 | 0.000800 | 15 | | PyCO\_scf\_00016\_3634\_132 | 0.001300 | 15 | | PyCO\_scf\_00043\_728\_76 | 0.002600 | 15 | | PyCO\_scf\_00781\_665\_83 | 0.002600 | 15 | | PyCO\_scf\_19577\_8\_112 | 0.002600 | 15 | | RiCO\_ctg\_29676\_57\_165 | 0.001800 | 15 | | RiCO\_ctg\_29676\_57\_89 | 0.001800 | 15 | | RiCO\_ctg\_29827\_2918\_83 | 0.007000 | 15 | | ScPA\_chr\_1-2\_6237\_71 | 0.001300 | 15 | | ScPA\_chr\_2-4\_32069\_96 | 0.000700 | 15 | | ScPA\_chr\_4-2\_312\_148 | 0.001300 | 15 | | ScPA\_chr\_5-1\_14490\_150 | 0.000300 | 15 | | ScPA\_chr\_5-1\_14490\_98 | 0.000300 | 15 | | SeIN\_lg\_15\_9787\_77 | 0.005000 | 15 | | SeIN\_lg\_15\_9821\_107 | 0.010000 | 15 | | SeIN\_lg\_3\_62711\_91 | 0.010000 | 15 | | SeIT\_chr\_2\_133410\_71 | 0.039600 | 15 | | SeIT\_chr\_6\_126145\_188 | 0.039600 | 15 | | SeIT\_chr\_9\_300280\_83 | 0.039600 | 15 | | SiIR\_scf\_20\_535\_108 | 0.000200 | 15 | | SiIR\_scf\_20\_535\_232 | 0.000200 | 15 | | SiIR\_scf\_29\_6036\_65 | 0.001000 | 15 | | SiIR\_scf\_8\_11134\_77 | 0.001000 | 15 | | SiIR\_scf\_8\_11159\_61 | 0.001000 | 15 | | SoBI\_chr\_01\_340810\_85 | 0.022200 | 15 | | SoBI\_chr\_02\_233223\_75 | 0.022200 | 15 | | SoBI\_chr\_10\_225742\_72 | 0.022200 | 15 | | SoLY\_chr\_03\_288882\_103 | 0.015000 | 15 | | SoPI\_ctg\_4939319\_12\_87 | 0.015000 | 15 | | SoPI\_ctg\_6742575\_3\_114 | 0.015000 | 15 | | SoTU\_chr\_02\_126880\_100 | 0.016700 | 15 | | SoTU\_chr\_08\_156316\_75 | 0.016700 | 15 | | SpOL\_scf\_21396\_72\_142 | 0.001400 | 15 | | SpOL\_scf\_21396\_72\_238 | 0.001400 | 15 | | SpOL\_scf\_21396\_72\_349 | 0.001400 | 15 | | SpOL\_scf\_21396\_72\_477 | 0.001400 | 15 | | SpOL\_scf\_21396\_72\_78 | 0.001400 | 15 | | SpOL\_scf\_25714\_134\_121 | 0.019100 | 15 | | SpOL\_scf\_25714\_168\_336 | 0.003800 | 15 | | SpOL\_scf\_33414\_32\_87 | 0.019100 | 15 | | SpPO\_chr\_4\_56998\_171 | 0.375000 | 15 | | TaHA\_scf\_10\_4\_230 | 0.004200 | 15 | | TaHA\_scf\_21\_4981\_72 | 0.004200 | 15 | | TaHA\_scf\_33\_10708\_113 | 0.002100 | 15 | | TaHA\_scf\_3\_40860\_86 | 0.004200 | 15 | | ThCA.Cr\_chr\_01\_161913\_84 | 0.006300 | 15 | | ThCA.Cr\_chr\_01\_161958\_88 | 0.006300 | 15 | | ThCA.Cr\_chr\_08\_1541\_121 | 0.006300 | 15 | | ThCA.Ma\_chr\_1\_202461\_153 | 0.006300 | 15 | | ThCA.Ma\_chr\_1\_202485\_213 | 0.006300 | 15 | | ThCA.Ma\_chr\_8\_113480\_99 | 0.006300 | 15 | | TrUR\_scf\_38417\_104\_81 | 0.033400 | 15 | | UtGI\_scf\_00238\_177\_76 | 0.075100 | 15 | | ViAN\_ctg\_11585\_0\_37\_111 | 0.002000 | 15 | | ViAN\_scf\_14509\_12\_162 | 0.001000 | 15 | | ViAN\_scf\_2025\_123\_85 | 0.002000 | 15 | | ViVI\_chr\_2\_50414\_78 | 0.316400 | 15 | | ZeMA\_chr\_4\_856145\_124 | 0.035600 | 15 | | | Sequence name | Weighting | Length | | --- | --- | --- | | AeAR\_scf\_13\_713\_155 | 0.001100 | 15 | | AeAR\_scf\_2092\_3\_67 | 0.004500 | 15 | | AeAR\_scf\_8\_9492\_72 | 0.004500 | 15 | | AeTA\_scf\_31116\_316\_81 | 0.025000 | 15 | | AeTA\_scf\_7016\_496\_65 | 0.025000 | 15 | | AmTR\_scf\_00157\_241\_75 | 0.111100 | 15 | | AmTR\_scf\_00157\_6321\_111 | 0.111100 | 15 | | AmTR\_scf\_00240\_313\_185 | 0.027800 | 15 | | AmTR\_scf\_00240\_74\_117 | 0.037000 | 15 | | AmTR\_scf\_00270\_119\_63 | 0.055600 | 15 | | AmTR\_scf\_00270\_48\_175 | 0.022200 | 15 | | AmTR\_scf\_00270\_48\_270 | 0.022200 | 15 | | AqCO\_scf\_15\_2715\_140 | 0.125000 | 15 | | AqCO\_scf\_15\_2745\_75 | 0.125000 | 15 | | ArDU\_chr\_01\_22525\_80 | 0.005300 | 15 | | ArDU\_chr\_05\_21712\_77 | 0.005300 | 15 | | ArDU\_chr\_06\_314502\_129 | 0.005300 | 15 | | ArIP\_chr\_05\_24658\_71 | 0.006000 | 15 | | ArIP\_chr\_05\_897879\_80 | 0.006000 | 15 | | ArLY\_scf\_1\_64585\_88 | 0.000900 | 15 | | ArLY\_scf\_2\_10720\_165 | 0.000100 | 15 | | ArLY\_scf\_2\_10720\_240 | 0.000100 | 15 | | ArLY\_scf\_2\_10720\_315 | 0.000100 | 15 | | ArLY\_scf\_2\_10720\_390 | 0.000100 | 15 | | ArLY\_scf\_4\_5687\_63 | 0.000900 | 15 | | ArLY\_scf\_5\_79011\_128 | 0.000200 | 15 | | ArLY\_scf\_5\_79011\_239 | 0.000200 | 15 | | ArLY\_scf\_8\_33647\_140 | 0.000100 | 15 | | ArLY\_scf\_8\_33647\_215 | 0.000100 | 15 | | ArLY\_scf\_8\_33647\_290 | 0.000100 | 15 | | ArLY\_scf\_8\_33647\_365 | 0.000100 | 15 | | ArLY\_scf\_8\_33647\_440 | 0.000100 | 15 | | ArLY\_scf\_8\_63500\_90 | 0.000500 | 15 | | ArTH\_chr\_1\_118662\_119 | 0.000400 | 15 | | ArTH\_chr\_1\_42237\_111 | 0.000300 | 15 | | ArTH\_chr\_1\_53133\_134 | 0.000800 | 15 | | ArTH\_chr\_2\_76174\_76 | 0.000800 | 15 | | ArTH\_chr\_3\_89083\_233 | 0.000200 | 15 | | ArTH\_chr\_5\_82442\_124 | 0.000800 | 15 | | ArTH\_chr\_5\_82453\_67 | 0.000800 | 15 | | AzIN\_scf\_17876\_1\_26\_75 | 0.001600 | 15 | | AzIN\_scf\_18451\_1\_5\_104 | 0.001600 | 15 | | AzIN\_scf\_2043\_2\_22\_46 | 0.003100 | 15 | | AzIN\_scf\_2207\_1\_23\_156 | 0.001000 | 15 | | AzIN\_scf\_2708\_1\_18\_131 | 0.003100 | 15 | | AzIN\_scf\_2708\_2\_71\_111 | 0.003100 | 15 | | AzIN\_scf\_42817\_1\_25\_121 | 0.003100 | 15 | | BeNA\_scf\_14434\_133\_110 | 0.012500 | 15 | | BeNA\_scf\_16996\_46\_81 | 0.012500 | 15 | | BeNA\_scf\_7080\_88\_107 | 0.012500 | 15 | | BeVU\_chr\_6.sca003\_7722\_111 | 0.026700 | 15 | | BeVU\_chr\_6.sca003\_7759\_239 | 0.006700 | 15 | | BeVU\_chr\_6.sca003\_7843\_117 | 0.026700 | 15 | | BrDI\_chr\_1\_402157\_80 | 0.035600 | 15 | | BrRA\_chr\_01\_110059\_103 | 0.000200 | 15 | | BrRA\_chr\_02\_76900\_109 | 0.000500 | 15 | | BrRA\_chr\_04\_29832\_96 | 0.000500 | 15 | | BrRA\_chr\_04\_76977\_174 | 0.000300 | 15 | | BrRA\_chr\_07\_114992\_118 | 0.000500 | 15 | | BrRA\_chr\_07\_115039\_106 | 0.000500 | 15 | | BrRA\_chr\_09\_128063\_65 | 0.000500 | 15 | | BrRA\_chr\_09\_146618\_208 | 0.000100 | 15 | | BrRA\_chr\_09\_208495\_60 | 0.000500 | 15 | | CaAN\_chr\_02\_414050\_125 | 0.020000 | 15 | | CaAN\_chr\_12\_556914\_82 | 0.020000 | 15 | | CaCA\_lg\_06\_45369\_90 | 0.001100 | 15 | | CaCA\_lg\_07\_34851\_94 | 0.002100 | 15 | | CaCA\_lg\_10\_34412\_108 | 0.002100 | 15 | | CaCA\_scf\_127737\_1130\_86 | 0.002100 | 15 | | CaCA\_scf\_133302\_184\_142 | 0.001100 | 15 | | CaCA\_scf\_133302\_222\_88 | 0.001100 | 15 | | CaCH\_scf\_110772\_34\_125 | 0.020000 | 15 | | CaCH\_scf\_44597\_8\_79 | 0.020000 | 15 | | CaPA\_ctg\_55\_3035\_58 | 0.011300 | 15 | | CaRU\_scf\_1\_36405\_85 | 0.001300 | 15 | | CaRU\_scf\_4\_3263\_107 | 0.001300 | 15 | | CaRU\_scf\_5\_58226\_158 | 0.000300 | 15 | | CaRU\_scf\_5\_58226\_54 | 0.000300 | 15 | | CaRU\_scf\_8\_42175\_60 | 0.001300 | 15 | | CaSA.Fi\_ctg\_14326822\_1\_100 | 0.006000 | 15 | | CaSA.Fi\_ctg\_14480433\_2\_119 | 0.006000 | 15 | | CaSA.PK\_scf\_3971\_17\_150 | 0.008400 | 15 | | CaSA.PK\_scf\_63384\_15\_68 | 0.008400 | 15 | | CiAR.De\_lg\_3\_44114\_98 | 0.004500 | 15 | | CiAR.Ka\_chr\_7\_183045\_93 | 0.002000 | 15 | | CiAR.Ka\_chr\_7\_27979\_74 | 0.001000 | 15 | | CiAR.Ka\_chr\_7\_28012\_70 | 0.002000 | 15 | | CiAR.Ka\_chr\_8\_10022\_61 | 0.002000 | 15 | | CiCL\_scf\_5\_107501\_93 | 0.006000 | 15 | | CiCL\_scf\_7\_106263\_95 | 0.003000 | 15 | | CiCL\_scf\_8\_84116\_94 | 0.006000 | 15 | | CiLA\_chr\_1\_108387\_184 | 0.009400 | 15 | | CiLA\_chr\_2\_56957\_84 | 0.004700 | 15 | | CiSI\_scf\_00006\_4933\_148 | 0.006000 | 15 | | CiSI\_scf\_00006\_4973\_85 | 0.006000 | 15 | | CiSI\_scf\_00082\_585\_94 | 0.006000 | 15 | | CuME\_chr\_08\_112716\_95 | 0.009400 | 15 | | CuME\_chr\_11\_2084\_115 | 0.004700 | 15 | | CuSA\_chr\_2\_34874\_70 | 0.004700 | 15 | | CuSA\_chr\_6\_94274\_95 | 0.009400 | 15 | | DiCA\_scf\_1239\_8\_73 | 0.014800 | 15 | | DiCA\_scf\_234\_252\_101 | 0.014800 | 15 | | DiCA\_scf\_234\_262\_244 | 0.004900 | 15 | | DiCA\_scf\_234\_290\_213 | 0.004900 | 15 | | DiCA\_scf\_477\_135\_66 | 0.014800 | 15 | | DiCA\_scf\_82\_1375\_144 | 0.014800 | 15 | | ElGU\_chr\_15\_38527\_418 | 0.039600 | 15 | | ElGU\_chr\_7\_48393\_96 | 0.039600 | 15 | | ElOL\_scf\_00733\_1061\_823 | 0.059300 | 15 | | EnVE\_ctg\_22030\_27790\_1\_28\_176 | 0.052700 | 15 | | EuCA\_ctg\_004830\_158\_147 | 0.002200 | 15 | | EuCA\_ctg\_007672\_9\_159 | 0.001100 | 15 | | EuCA\_ctg\_010936\_1\_92 | 0.001100 | 15 | | EuCA\_ctg\_011241\_14\_93 | 0.002200 | 15 | | EuCA\_ctg\_014037\_37\_119 | 0.002200 | 15 | | EuCA\_ctg\_018397\_20\_217 | 0.000700 | 15 | | EuCA\_ctg\_018397\_212\_75 | 0.000600 | 15 | | EuCA\_ctg\_023070\_58\_136 | 0.000200 | 15 | | EuCA\_ctg\_023070\_58\_202 | 0.000200 | 15 | | EuCA\_ctg\_023070\_58\_268 | 0.000200 | 15 | | EuCA\_ctg\_023293\_14\_116 | 0.001100 | 15 | | EuCA\_ctg\_023562\_184\_177 | 0.001100 | 15 | | EuCA\_ctg\_023562\_313\_134 | 0.001100 | 15 | | EuCA\_ctg\_025488\_47\_131 | 0.002200 | 15 | | EuCA\_ctg\_034442\_93\_149 | 0.001100 | 15 | | EuCA\_ctg\_046052\_114\_119 | 0.002200 | 15 | | EuCA\_ctg\_050450\_46\_93 | 0.002200 | 15 | | EuCA\_ctg\_051094\_155\_133 | 0.001100 | 15 | | EuCA\_ctg\_055172\_400\_184 | 0.002200 | 15 | | EuCA\_scf\_629621\_2\_196 | 0.000700 | 15 | | EuGR\_scf\_1325\_3\_158 | 0.001700 | 15 | | EuGR\_scf\_5\_144942\_159 | 0.001700 | 15 | | EuGR\_scf\_5\_238225\_75 | 0.000400 | 15 | | EuGR\_scf\_5\_238235\_141 | 0.000300 | 15 | | EuGR\_scf\_5\_238235\_97 | 0.000300 | 15 | | EuGR\_scf\_5\_238372\_158 | 0.000200 | 15 | | EuGR\_scf\_5\_238372\_224 | 0.000200 | 15 | | EuGR\_scf\_5\_238372\_290 | 0.000200 | 15 | | EuGR\_scf\_5\_240944\_159 | 0.001700 | 15 | | EuGR\_scf\_5\_333720\_218 | 0.000400 | 15 | | EuGR\_scf\_5\_333937\_136 | 0.000200 | 15 | | EuGR\_scf\_5\_333937\_202 | 0.000200 | 15 | | EuGR\_scf\_5\_333937\_268 | 0.000200 | 15 | | EuGR\_scf\_5\_333963\_136 | 0.000100 | 15 | | EuGR\_scf\_5\_333963\_202 | 0.000100 | 15 | | EuGR\_scf\_5\_333963\_268 | 0.000100 | 15 | | EuGR\_scf\_5\_333963\_334 | 0.000100 | 15 | | EuGR\_scf\_6\_108863\_103 | 0.001700 | 15 | | EuGR\_scf\_6\_1772\_95 | 0.000900 | 15 | | EuGR\_scf\_6\_193261\_122 | 0.001700 | 15 | | EuGR\_scf\_6\_193456\_107 | 0.001700 | 15 | | EuGR\_scf\_6\_70588\_99 | 0.001700 | 15 | | EuGR\_scf\_8\_143712\_190 | 0.000900 | 15 | | EuGR\_scf\_8\_418581\_121 | 0.000900 | 15 | | EuGR\_scf\_8\_418665\_133 | 0.000900 | 15 | | EuGR\_scf\_8\_418753\_166 | 0.000900 | 15 | | EuGR\_scf\_8\_418858\_131 | 0.000900 | 15 | | EuGR\_scf\_8\_418948\_218 | 0.000900 | 15 | | EuGR\_scf\_9\_63713\_93 | 0.001700 | 15 | | EuGR\_scf\_9\_63945\_151 | 0.001700 | 15 | | EuGR\_scf\_9\_64376\_141 | 0.001700 | 15 | | EuSA\_scf\_15\_6258\_73 | 0.001500 | 15 | | EuSA\_scf\_16\_7797\_199 | 0.000400 | 15 | | EuSA\_scf\_5\_91709\_95 | 0.001500 | 15 | | EuSA\_scf\_6\_1153\_108 | 0.001500 | 15 | | EuSA\_scf\_6\_1171\_90 | 0.001500 | 15 | | FrEX\_scf\_14360\_8\_123 | 0.004900 | 15 | | FrEX\_scf\_20018\_3\_122 | 0.009900 | 15 | | FrEX\_scf\_2186\_525\_73 | 0.009900 | 15 | | FrEX\_scf\_2486\_721\_108 | 0.009900 | 15 | | FrEX\_scf\_3405\_191\_116 | 0.004900 | 15 | | FrEX\_scf\_5019\_23\_120 | 0.004900 | 15 | | FrEX\_scf\_5423\_91\_92 | 0.009900 | 15 | | FrEX\_scf\_8251\_14\_104 | 0.009900 | 15 | | FrII\_scf\_00054285.1\_8\_87 | 0.010600 | 15 | | FrNI\_ctg\_04327039.1\_4\_109 | 0.002400 | 15 | | FrNI\_scf\_00040543.1\_3\_141 | 0.004800 | 15 | | FrNU\_scf\_00014410.1\_5\_84 | 0.004800 | 15 | | FrNU\_scf\_00078196.1\_34\_147 | 0.002400 | 15 | | FrVE\_lg\_2\_88445\_87 | 0.005300 | 15 | | FrVE\_lg\_6\_109503\_107 | 0.002600 | 15 | | FrVE\_lg\_6\_109525\_119 | 0.005300 | 15 | | GlMA\_chr\_01\_174694\_145 | 0.000400 | 15 | | GlMA\_chr\_01\_174721\_105 | 0.000900 | 15 | | GlMA\_chr\_05\_53925\_180 | 0.000400 | 15 | | GlMA\_chr\_06\_276641\_130 | 0.000900 | 15 | | GlMA\_chr\_11\_11233\_116 | 0.000400 | 15 | | GlMA\_chr\_11\_11263\_104 | 0.000900 | 15 | | GlMA\_chr\_16\_157295\_74 | 0.000900 | 15 | | GlMA\_chr\_17\_184446\_120 | 0.000400 | 15 | | GlMA\_chr\_17\_184560\_114 | 0.000400 | 15 | | GlMA\_chr\_17\_184876\_120 | 0.000400 | 15 | | GlMA\_chr\_17\_185246\_118 | 0.000900 | 15 | | GoRA\_chr\_03\_227050\_71 | 0.003400 | 15 | | GoRA\_chr\_03\_227124\_82 | 0.003400 | 15 | | GoRA\_chr\_03\_227227\_82 | 0.003400 | 15 | | GoRA\_chr\_03\_227267\_75 | 0.003400 | 15 | | GoRA\_chr\_04\_120962\_88 | 0.003400 | 15 | | GoRA\_chr\_07\_252200\_102 | 0.003400 | 15 | | GoRA\_chr\_08\_258111\_85 | 0.003400 | 15 | | GoRA\_chr\_10\_309123\_56 | 0.003400 | 15 | | HeBR\_scf\_155851\_130\_120 | 0.002400 | 15 | | HeBR\_scf\_155851\_157\_203 | 0.000600 | 15 | | HeBR\_scf\_155851\_99\_81 | 0.002400 | 15 | | HeBR\_scf\_243691\_91\_88 | 0.000800 | 15 | | HeBR\_scf\_420305\_30\_75 | 0.002400 | 15 | | HoVU\_ctg\_853286\_22\_86 | 0.026700 | 15 | | JaCU\_scf\_02121\_40\_129 | 0.000800 | 15 | | JaCU\_scf\_02121\_40\_308 | 0.000800 | 15 | | JaCU\_scf\_04617\_69\_70 | 0.004500 | 15 | | JaCU\_scf\_12633\_34\_103 | 0.004500 | 15 | | KaFE\_scf\_1360502\_67\_85 | 0.063300 | 15 | | KaFE\_scf\_1368498\_168\_107 | 0.063300 | 15 | | LaSI\_ctg\_303226\_5\_88 | 0.014100 | 15 | | LeAL\_scf\_12\_1242\_72 | 0.001600 | 15 | | LeAL\_scf\_217\_537\_151 | 0.000500 | 15 | | LeAL\_scf\_26\_8556\_79 | 0.001600 | 15 | | LeAL\_scf\_38\_774\_88 | 0.001600 | 15 | | LeAL\_scf\_72\_10087\_66 | 0.001600 | 15 | | LiUS\_scf\_1249\_1842\_139 | 0.000600 | 15 | | LiUS\_scf\_1249\_1842\_242 | 0.000600 | 15 | | LiUS\_scf\_1249\_1842\_352 | 0.000600 | 15 | | LiUS\_scf\_2416\_588\_124 | 0.000700 | 15 | | LiUS\_scf\_2416\_588\_203 | 0.000700 | 15 | | LiUS\_scf\_2416\_588\_318 | 0.000700 | 15 | | LiUS\_scf\_464\_9241\_88 | 0.004700 | 15 | | LiUS\_scf\_66\_415\_106 | 0.004700 | 15 | | LoJA\_chr\_5\_164713\_89 | 0.015800 | 15 | | LuAN\_scf\_13935\_13\_67 | 0.004400 | 15 | | LuAN\_scf\_18977\_68\_69 | 0.004400 | 15 | | LuAN\_scf\_65780\_36\_67 | 0.004400 | 15 | | LuAN\_scf\_76000\_38\_68 | 0.004400 | 15 | | LuAN\_scf\_76092\_44\_98 | 0.004400 | 15 | | LuAN\_scf\_80590\_1\_7\_98 | 0.004400 | 15 | | MaDO\_chr\_15\_179568\_115 | 0.005300 | 15 | | MaDO\_chr\_15\_8403\_76 | 0.005300 | 15 | | MaDO\_chr\_2\_149034\_94 | 0.002600 | 15 | | MaES\_chr\_viii\_36047\_169 | 0.000500 | 15 | | MaES\_chr\_viii\_36047\_75 | 0.000500 | 15 | | MaES\_chr\_x\_67218\_114 | 0.001000 | 15 | | MaES\_chr\_xii\_50581\_89 | 0.002000 | 15 | | MaES\_chr\_xviii\_47380\_659 | 0.001000 | 15 | | MaES\_scf\_01179\_505\_71 | 0.002000 | 15 | | MeTR\_chr\_5\_197996\_73 | 0.001800 | 15 | | MeTR\_chr\_5\_33063\_115 | 0.000900 | 15 | | MeTR\_ctg\_59554\_7\_127 | 0.000900 | 15 | | MeTR\_scf\_ac233112.6\_159\_120 | 0.000900 | 15 | | MeTR\_scf\_ac233112.6\_181\_104 | 0.000900 | 15 | | MeTR\_scf\_ac233112.6\_695\_68 | 0.001800 | 15 | | MiGU\_scf\_5\_94256\_71 | 0.019100 | 15 | | MiGU\_scf\_8\_8701\_66 | 0.009500 | 15 | | MoNO\_scf\_123\_420\_102 | 0.009400 | 15 | | MoNO\_scf\_256\_2508\_89 | 0.009400 | 15 | | MuAC\_chr\_1\_141422\_67 | 0.033900 | 15 | | MuAC\_chr\_6\_24028\_94 | 0.033900 | 15 | | MuAC\_chr\_un\_random\_176676\_97 | 0.033900 | 15 | | MuBA\_chr\_3\_168239\_103 | 0.033900 | 15 | | MuBA\_chr\_9\_55857\_148 | 0.017000 | 15 | | NeNU\_scf\_00003\_13710\_87 | 0.051100 | 15 | | NeNU\_scf\_00003\_56239\_98 | 0.051100 | 15 | | NeNU\_scf\_00099\_6847\_125 | 0.051100 | 15 | | NeNU\_scf\_00099\_7001\_128 | 0.025600 | 15 | | NiBE\_scf\_118\_3224\_96 | 0.009500 | 15 | | NiBE\_scf\_1510\_2572\_77 | 0.009500 | 15 | | NiBE\_scf\_465\_3928\_111 | 0.009500 | 15 | | NiBE\_scf\_5078\_118\_71 | 0.009500 | 15 | | NiBE\_scf\_7636\_341\_80 | 0.009500 | 15 | | NiSY\_scf\_1546\_797\_82 | 0.011100 | 15 | | NiSY\_scf\_26250\_274\_88 | 0.011100 | 15 | | NiSY\_scf\_3607\_327\_74 | 0.011100 | 15 | | NiTO\_scf\_10718\_593\_73 | 0.010000 | 15 | | NiTO\_scf\_22023\_508\_79 | 0.010000 | 15 | | NiTO\_scf\_3364\_1326\_203 | 0.010000 | 15 | | OrBR\_chr\_3\_153092\_92 | 0.039600 | 15 | | OrBR\_chr\_6\_74540\_95 | 0.039600 | 15 | | OrGL\_chr\_06\_44603\_73 | 0.178000 | 15 | | OrSA.In\_chr\_03\_245043\_155 | 0.005600 | 15 | | OrSA.In\_chr\_03\_68043\_104 | 0.022200 | 15 | | OrSA.In\_chr\_09\_64645\_75 | 0.022200 | 15 | | OrSA.Ja\_chr\_03\_226607\_155 | 0.005600 | 15 | | OrSA.Ja\_chr\_03\_64155\_114 | 0.022200 | 15 | | OrSA.Ja\_chr\_09\_68574\_75 | 0.022200 | 15 | | PaHA\_scf\_147\_2797\_61 | 0.014800 | 15 | | PaHA\_scf\_45\_6570\_68 | 0.029700 | 15 | | PaVI\_chr\_02a\_355916\_87 | 0.012700 | 15 | | PaVI\_chr\_09a\_177585\_140 | 0.006400 | 15 | | PaVI\_chr\_09b\_67367\_89 | 0.006400 | 15 | | PaVI\_ctg\_05592\_97\_86 | 0.012700 | 15 | | PaVI\_ctg\_280593\_10\_69 | 0.012700 | 15 | | PhDA\_scf\_000017\_10633\_135 | 0.052700 | 15 | | PhDA\_scf\_000143\_2896\_73 | 0.052700 | 15 | | PhHE\_scf\_01000559\_4478\_77 | 0.029700 | 15 | | PhHE\_scf\_01002760\_234\_79 | 0.029700 | 15 | | PhHE\_scf\_01004822\_313\_89 | 0.029700 | 15 | | PhVU\_chr\_02\_189930\_69 | 0.001500 | 15 | | PhVU\_chr\_03\_128027\_115 | 0.000700 | 15 | | PhVU\_chr\_03\_128036\_160 | 0.000700 | 15 | | PhVU\_chr\_03\_128129\_94 | 0.001500 | 15 | | PhVU\_chr\_03\_153596\_84 | 0.001500 | 15 | | PiAB\_ctg\_18434\_210\_178 | 0.070300 | 15 | | PiAB\_ctg\_490634\_20\_149 | 0.070300 | 15 | | PiGL\_ctg\_162771921\_0\_12\_163 | 0.030100 | 15 | | PiGL\_ctg\_165043132\_0\_3\_153 | 0.030100 | 15 | | PiGL\_ctg\_167815528\_5\_4\_183 | 0.030100 | 15 | | PiGL\_ctg\_168009378\_8\_28\_159 | 0.030100 | 15 | | PiGL\_ctg\_30784271\_0\_25\_136 | 0.030100 | 15 | | PiTA\_ctg\_28724920\_2\_129 | 0.020100 | 15 | | PiTA\_scf\_184314\_493\_135 | 0.020100 | 15 | | PiTA\_scf\_306645\_59\_149 | 0.020100 | 15 | | PiTA\_scf\_585603\_36\_135 | 0.020100 | 15 | | PiTA\_scf\_881528\_51\_149 | 0.020100 | 15 | | PiTA\_scf\_t2391\_529\_171 | 0.020100 | 15 | | PiTA\_scf\_t5839\_2326\_171 | 0.020100 | 15 | | PoEU\_scf\_14.1\_5334\_104 | 0.006300 | 15 | | PoEU\_scf\_234.1\_203\_154 | 0.001300 | 15 | | PoEU\_scf\_234.1\_203\_272 | 0.001300 | 15 | | PoEU\_scf\_234.1\_363\_104 | 0.006300 | 15 | | PoEU\_scf\_354.1\_893\_181 | 0.002100 | 15 | | PoEU\_scf\_623.1\_696\_181 | 0.002100 | 15 | | PoTR\_chr\_05\_109127\_118 | 0.005100 | 15 | | PoTR\_chr\_05\_109193\_182 | 0.001000 | 15 | | PoTR\_chr\_07\_72626\_128 | 0.005100 | 15 | | PoTR\_chr\_07\_72674\_188 | 0.000600 | 15 | | PoTR\_chr\_07\_72674\_377 | 0.000600 | 15 | | PoTR\_chr\_07\_72718\_128 | 0.005100 | 15 | | PoTR\_chr\_07\_72858\_188 | 0.000600 | 15 | | PoTR\_chr\_07\_72858\_416 | 0.000600 | 15 | | PoTR\_chr\_07\_72858\_538 | 0.000600 | 15 | | PoTR\_chr\_14\_7933\_75 | 0.005100 | 15 | | PrMU\_chr\_7\_107295\_69 | 0.006300 | 15 | | PrMU\_chr\_8\_31255\_80 | 0.006300 | 15 | | PrPE\_scf\_5\_119216\_70 | 0.005300 | 15 | | PrPE\_scf\_7\_52252\_171 | 0.002600 | 15 | | PyBR\_scf\_136.0\_4568\_83 | 0.001600 | 15 | | PyBR\_scf\_154.0\_460\_76 | 0.001600 | 15 | | PyBR\_scf\_190.0\_3174\_83 | 0.001600 | 15 | | PyBR\_scf\_4.0\_9723\_112 | 0.001600 | 15 | | PyBR\_scf\_4.0\_9812\_73 | 0.001600 | 15 | | PyBR\_scf\_520.0\_2943\_133 | 0.000800 | 15 | | PyCO\_scf\_00016\_3634\_99 | 0.001300 | 15 | | PyCO\_scf\_00343\_1191\_86 | 0.002600 | 15 | | PyCO\_scf\_03185\_22\_100 | 0.001300 | 15 | | PyCO\_scf\_33884\_6\_76 | 0.002600 | 15 | | RiCO\_ctg\_29676\_57\_246 | 0.001800 | 15 | | RiCO\_ctg\_29676\_74\_79 | 0.007000 | 15 | | RiCO\_ctg\_30170\_4822\_82 | 0.007000 | 15 | | ScPA\_chr\_2-4\_32057\_77 | 0.001300 | 15 | | ScPA\_chr\_2-4\_32077\_91 | 0.001300 | 15 | | ScPA\_chr\_4-2\_333\_105 | 0.001300 | 15 | | ScPA\_chr\_5-1\_14490\_202 | 0.000300 | 15 | | SeIN\_lg\_15\_9726\_84 | 0.010000 | 15 | | SeIN\_lg\_15\_9806\_116 | 0.010000 | 15 | | SeIN\_lg\_1\_108320\_81 | 0.010000 | 15 | | SeIN\_lg\_8\_120417\_68 | 0.010000 | 15 | | SeIT\_chr\_2\_133426\_66 | 0.039600 | 15 | | SeIT\_chr\_9\_170607\_82 | 0.039600 | 15 | | SeIT\_chr\_9\_300311\_79 | 0.039600 | 15 | | SiIR\_scf\_20\_535\_160 | 0.000200 | 15 | | SiIR\_scf\_20\_535\_56 | 0.000200 | 15 | | SiIR\_scf\_29\_6054\_87 | 0.001000 | 15 | | SiIR\_scf\_8\_11146\_59 | 0.000500 | 15 | | SiIR\_scf\_8\_11167\_81 | 0.001000 | 15 | | SoBI\_chr\_01\_340892\_101 | 0.022200 | 15 | | SoBI\_chr\_07\_206797\_80 | 0.022200 | 15 | | SoLY\_chr\_02\_127699\_87 | 0.015000 | 15 | | SoLY\_chr\_08\_164998\_78 | 0.015000 | 15 | | SoPI\_ctg\_5086539\_16\_67 | 0.015000 | 15 | | SoPI\_ctg\_870016\_5\_103 | 0.015000 | 15 | | SoTU\_chr\_03\_20601\_107 | 0.016700 | 15 | | SoTU\_chr\_12\_140322\_102 | 0.016700 | 15 | | SpOL\_scf\_21396\_72\_174 | 0.001400 | 15 | | SpOL\_scf\_21396\_72\_285 | 0.001400 | 15 | | SpOL\_scf\_21396\_72\_413 | 0.001400 | 15 | | SpOL\_scf\_21396\_72\_509 | 0.001400 | 15 | | SpOL\_scf\_24951\_21\_107 | 0.019100 | 15 | | SpOL\_scf\_25714\_168\_240 | 0.003800 | 15 | | SpOL\_scf\_25714\_168\_372 | 0.003800 | 15 | | SpOL\_scf\_33414\_69\_78 | 0.019100 | 15 | | TaHA\_scf\_10\_1346\_102 | 0.002100 | 15 | | TaHA\_scf\_10\_9\_81 | 0.004200 | 15 | | TaHA\_scf\_2\_25572\_95 | 0.004200 | 15 | | TaHA\_scf\_33\_10708\_72 | 0.002100 | 15 | | ThCA.Cr\_chr\_00\_28530\_111 | 0.003100 | 15 | | ThCA.Cr\_chr\_01\_161924\_153 | 0.006300 | 15 | | ThCA.Cr\_chr\_03\_68864\_77 | 0.006300 | 15 | | ThCA.Cr\_chr\_08\_64055\_99 | 0.006300 | 15 | | ThCA.Ma\_chr\_1\_202475\_111 | 0.003100 | 15 | | ThCA.Ma\_chr\_1\_202497\_88 | 0.006300 | 15 | | ThCA.Ma\_chr\_8\_1795\_109 | 0.006300 | 15 | | TrUR\_scf\_69099\_94\_65 | 0.033400 | 15 | | ViAN\_ctg\_11418\_0\_91\_73 | 0.002000 | 15 | | ViAN\_scf\_11539\_37\_63 | 0.002000 | 15 | | ViAN\_scf\_14509\_28\_104 | 0.001000 | 15 | | ViAN\_scf\_2025\_131\_125 | 0.002000 | 15 | | ZeMA\_chr\_1\_871253\_86 | 0.035600 | 15 | | ZeMA\_chr\_5\_1636826\_68 | 0.035600 | 15 | |

hide training set...

##### Command line summary

meme Embryophyta\_domains.fasta -oc Embryophyta -mod oops -w 15   
Letter frequencies in dataset  

A: 0.052   C: 0.000   D: 0.033   E: 0.005   F: 0.033   G: 0.208   H: 0.120   I: 0.027   K: 0.002   L: 0.010   M: 0.004   N: 0.019   P: 0.176   Q: 0.006   R: 0.060   S: 0.133   T: 0.072   V: 0.033   W: 0.001   Y: 0.004

  
Background letter frequencies (from dataset with add-one prior applied):  

A: 0.052   C: 0.004   D: 0.035   E: 0.008   F: 0.035   G: 0.195   H: 0.114   I: 0.029   K: 0.006   L: 0.013   M: 0.008   N: 0.021   P: 0.166   Q: 0.010   R: 0.060   S: 0.127   T: 0.070   V: 0.035   W: 0.005   Y: 0.008

##### Stopping Reason

Stopped because nmotifs = 1 reached. Program ran on *prometheus*.

show model parameters...

##### Model parameters

host = prometheus
type = oops
nmotifs = 1
evalue\_threshold = inf
object\_function = E-value of product of p-values
min\_width = 15
max\_width = 15
minic = 0.00
wg = 11
ws = 1
endgaps = yes
minsites = 1189
maxsites = 1189
wnsites = 0.8
prob = 1
spmap = pam
spfuzz = 120
prior = dmix
beta = 0
maxiter = 50
distance = 1e-05
num\_sequences = 1189
num\_positions = 17835
seed = 0
seqfrac = 1
strands = none
priors\_file = prior30.plib
reason\_for\_stopping = Stopped because nmotifs = 1 reached.

hide model parameters...

|  |  |
| --- | --- |
| Explanation of MEME Results | Top |

#### The MEME results consist of

- Information on each of the **motifs** MEME discovered, including:
  1. A **summary table** showing the width, number of contributing sites, log likelihood ratio,
     statistical significance, information content and relative entropy of the motif.
  2. A **sequence LOGO**.
  3. A **regular expression** describing the motif.
  4. A list of **data formats** describing the motif.
  5. The **contributing sites** of the motif sorted by *p*-value and aligned with each other.
- The **version** of MEME and the date it was released.
- The **reference** to cite if you use MEME in your research.
- A description of the **sequences** you submitted (the "training set") showing the name,
  "weight" and length of each sequence.
- The **command line summary** detailing the parameters with which you ran MEME.
- The reason why MEME **stopped** and the name of the CPU on which it ran.
- This **explanation** of how to interpret MEME results.

#### Motifs

For each motif that it discovers in the training set,
MEME prints the following information:

##### Summary Table

This summary table gives the main attributes of the motif.

*E*-value
:   The statistical significance of the motif. MEME usually finds the most statistically significant (low *E*-value) motifs first.
    The *E*-value of a motif is based on its log likelihood ratio, width, sites, the background letter frequencies (given in the
    command line summary), and the size of the training set. The *E*-value is an estimate of the expected
    number of motifs with the given log likelihood ratio (or higher), and with the same width and site count, that one would find in
    a similarly sized set of random sequences. (In random sequences each position is independent with letters chosen according to
    the background letter frequencies.)

Width
:   The width of the motif. Each motif describes a pattern of a fixed with as no gaps are allowed in MEME motifs.

Sites
:   The number of sites contributing to the construction of the motif.

Log Likelihood Ratio
:   The log likelihood ratio of the motif.The log likelihood ratio is the logarithm of the ratio of the probability of the occurrences
    of the motif given the motif model (likelihood given the motif) versus their probability given the background model
    (likelihood given the null model). (Normally the background model is a 0-order Markov model using the background letter
    frequencies, but higher order Markov models may be specified via the **-bfile** option to MEME.)

Information Content
:   The information content of the motif in bits. It is equal to the sum of the **uncorrected** information content, R(),
    in the columns of the LOGO. This is equal relative entropy of the motif relative to a uniform background frequency
    model.

Relative Entropy
:   The relative entropy of the motif, computed in bits and relative to the background letter frequencies given in the
    command line summary. It is equal to the log-likelihood ratio (llr) divided by the
    number of contributing sites of the motif times 1/ln(2),  
      
    re = llr / (sites \* ln(2)).

##### Sequence LOGO

MEME motifs are represented by position-specific probability matrices
that specify the probability of each possible letter appearing at each
possible position in an occurrence of the motif. These are displayed
as "sequence LOGOS", containing stacks of letters at each position
in the motif. The total height of the stack is the "information
content" of that position in the motif in bits. The height of the
individual letters in a stack is the probability of the letter at that
position multiplied by the total information content of the stack.

Modern web browsers supporting the canvas element and it's text manipulation functions as described in the
html 5 standard, can render the sequence LOGOs without needing the images. The browsers which work with this
feature are:

- Firefox 3.5 and above
- Safari 4 and above
- Google Chrome 4 and above

Unfortunately Internet Explorer 8 does not support any html 5 features.

The information content of each motif position is computed as described in the paper by Schneider and Stephens,
"Sequence Logos: A New Way to Display Consensus Sequences" but
**the small-sample correction, e(n), is set to zero for the LOGO displayed in the MEME output.**
The corrected information content of position i is given by

```
            R(i) for amino acids   = log2(20) - (H(i) + e(n))   (1a) 
            R(i) for nucleic acids =    2    - (H(i) + e(n))    (1b)
```

where H(i) is the entropy of position i,

```
            H(l) = - (Sum f(a,i) * log2[ f(a,i) ]).             (2)
```

Here, f(a,i) is the frequency of base or amino acid a at position i, and e(n) is the small-sample correction
for an alignment of n letters. The height of letter a in column i is given by

```
            height = f(a,i) * R(i)                              (3)
```

The approximation for the small-sample correction, e(n), is given by:

```
            e(n) = (s-1) / (2 * ln(2) * n),                     (4)
```

where s is 4 for nucleotides, 20 for amino acids, and n is the number of sequences in the alignment.

The letters in the logos are colored as follows.  
For DNA sequences, the letter categories contain one letter each.

| NUCLEIC ACIDS | COLOR |
| --- | --- |
| A | RED |
| C | BLUE |
| G | ORANGE |
| T | GREEN |

For proteins, the categories are based on the biochemical properties of the various amino acids.

| AMINO ACIDS | COLOR | PROPERTIES |
| --- | --- | --- |
| A, C, F, I, L, V, W and M | BLUE | Most hydrophobic[Kyte and Doolittle, 1982] |
| NQST | GREEN | Polar, non-charged, non-aliphatic residues |
| DE | MAGENTA | Acidic |
| KR | RED | Positively charged |
| H | PINK |  |
| G | ORANGE |  |
| P | YELLOW |  |
| Y | TURQUOISE |  |

J. Kyte and R. Doolittle, 1982. "A Simple Method for Displaying the Hydropathic Character of a Protein",
J. Mol Biol. 157, 105-132.

**Note:** the "text" output format of MEME preserves the historical MEME format where LOGOS are replaced by a simplified probability
matrix, a relative entropy plot, and a multi-level consensus sequence.

##### Regular Expression

This is a regular expression (RE) describing the motif. In each column, all letters with observed frequencies greater than 0.2 are shown;
less-frequent letters are not included in the RE. MEME regular expressions are interpreted as follows: single letters match that letter;
groups of letters in square brackets match any of the letters in the group. Regular expressions can be used for searching for the motif
in sequences (using, for example, PatMatch) but the search
accuracy will usually be better with the PSSM (using, for example MAST.)

##### Data Formats

The extracted data is avaliable in the following formats.

PSPM Format
:   The motif itself is a position-specific probability matrix giving, for each position in the pattern, the observed frequency ("probability")
    of each possible letter. The probability matrix is printed "sideways"--columns correspond to the letters in the alphabet (in the same order
    as shown in the simplified motif) and rows corresponding to the positions of the motif, position one first. The motif is preceded by a line
    starting with "letter-probability matrix:" and containing the length of the alphabet, width of the motif, number of occurrences of the motif,
    and the *E*-value of the motif.  
    **Note:** Earlier versions of MEME gave the posterior probabilities--the probability after applying a prior on letter frequencies--rather
    than the observed frequencies. These versions of MEME also gave the number of *possible* positions for the motif rather than the actual
    number of occurrences. The output from these earlier versions of MEME can be distinguished by "n=" rather than "nsites=" in the line
    preceding the matrix.

PSSM Format
:   The position-specific scoring matrix corresponding to the motif is printed for use by database search programs such as MAST.
    This matrix is a log-odds matrix calculated by taking 100 times the log (base 2) of the ratio *p/f* at each position in
    the motif where *p* is the probability of a particular letter at that position in the motif, and *f* is the background
    frequency of the letter (given in the command line summary section.) This is the same matrix that is used
    above in computing the *p*-values of the occurrences of the motif in the Sites section. The scoring matrix is printed "sideways"--columns
    correspond to the letters in the alphabet (in the same order as shown in the simplified motif) and rows corresponding to the
    positions of the motif, position one first. The scoring matrix is preceded by a line starting with "log-odds matrix:" and
    containing the length of the alphabet, width of the motif, number of characters in the training set, the scoring threshold
    (obsolete) and the motif *E*-value.  
    **Note:** The probability *p* used to compute the PSSM is *not* exactly the same as the corresponding value in the
    Position Specific Probability Matrix (PSPM). The values of *p* used to compute the PSSM take into account the motif prior,
    whereas the values in the PSPM are just the *observed* frequencies of letters in the motif sites.

BLOCKS Format
:   For use with BLOCKS tools.

FASTA Format
:   The FASTA format.

Raw Format
:   Just the sites of the sequences that contributed to the motif. One site per line.

##### Sites

MEME displays the occurrences (sites) of the motif in the training set. The sites are shown aligned with each other, and the ten sequence
positions preceding and following each site are also shown. Each site is identified by the name of the sequence where it occurs,
the strand (if both strands of DNA sequences are being used), and the position in the sequence where the site begins. When the DNA strand
is specified, '+' means the sequence in the training set, and '-' means the reverse complement of the training set sequence.
(For '-' strands, the 'start' position is actually the position on the **positive** strand where the site ends.) The sites are **listed
in order of increasing statistical significance** (*p*-value). The *p*-value of a site is computed from the the match score of
the site with the position specific scoring matrix for the motif. The *p*-value gives the probability of a
random string (generated from the background letter frequencies) having the same match score or higher. (This is referred to as the
**position *p*-value** by the MAST algorithm.)
