# Supplementary figures and images for: Diversification of the C-TERMINALLY ENCODED PEPTIDE (CEP) gene family in angiosperms, and evolution of plant-family specific CEP genes
Source: BMC Genomics. 2014 Oct 6;15(1):870. doi: 10.1186/1471-2164-15-870 (PMC4197245; doi:10.1186/1471-2164-15-870)

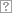

Supplement: Supplementary file 2 — Additional file 2: MEME output for the canonical CEP domain. Includes the CEP domain sequence logo and position-specific probability matrix (PSPM), iteratively generated from previously identified CEP domain sequences. (ZIP 108 KB) [file 12864_2014_6540_MOESM2_ESM.zip › help.gif]

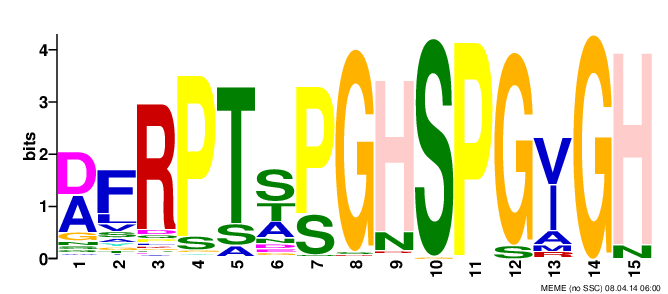

Supplement: Supplementary file 2 — Additional file 2: MEME output for the canonical CEP domain. Includes the CEP domain sequence logo and position-specific probability matrix (PSPM), iteratively generated from previously identified CEP domain sequences. (ZIP 108 KB) [file 12864_2014_6540_MOESM2_ESM.zip › logo1.png]
